# Implementation strategies to increase smoking cessation treatment provision in primary care: a systematic review of observational studies

**DOI:** 10.1186/s12875-023-01981-2

**Published:** 2023-01-25

**Authors:** Bernadett E. Tildy, Ann McNeill, Parvati R. Perman-Howe, Leonie S. Brose

**Affiliations:** 1 Addictions Department, King’s College London, Addiction Sciences Building, Denmark Hill Campus, 4 Windsor Walk, London, SE5 8BB UK; 2 SPECTRUM Consortium, London, UK

**Keywords:** Systematic literature review, Smoking, Smoking cessation, Tobacco, Substance use, Primary care, General practice, Implementation strategy, 5As, Very brief advice

## Abstract

**Background:**

Internationally, there is an ‘evidence-practice gap’ in the rate healthcare professionals assess tobacco use and offer cessation support in clinical practice, including primary care. Evidence is needed for implementation strategies enacted in the ‘real-world’. Aim: To identify implementation strategies aiming to increase smoking cessation treatment provision in primary care, their effectiveness, cost-effectiveness and any perceived facilitators and barriers for effectiveness.

**Methods:**

‘Embase’, ‘Medline’, ‘PsycINFO’, ‘CINAHL’, ‘Global Health’, ‘Social Policy & Practice’, ‘ASSIA Applied Social Sciences Index and Abstracts’ databases, and grey literature sources were searched from inception to April 2021. Studies were included if they evaluated an implementation strategy implemented on a nation-/state-wide scale, targeting any type of healthcare professional within the primary care setting, aiming to increase smoking cessation treatment provision. Primary outcome measures: implementation strategy identification, and effectiveness (practitioner-/patient-level). Secondary outcome measures: perceived facilitators and barriers to effectiveness, and cost-effectiveness. Studies were assessed using the Risk Of Bias In Non-randomized Studies of Interventions (ROBINS-I) tool. A narrative synthesis was conducted using the Expert Recommendations for Implementing Change (ERIC) compilation and the Consolidated Framework for Implementation Research (CFIR).

**Results:**

Of 49 included papers, half were of moderate/low risk of bias. The implementation strategy domains identified involved utilizing financial strategies, changing infrastructure, training and educating stakeholders, and engaging consumers. The first three increased practitioner-level smoking status recording and cessation advice provision. Interventions in the utilizing financial strategies domain also appeared to increase smoking cessation (patient-level). Key facilitator: external policies/incentives (tobacco control measures and funding for public health and cessation clinics). Key barriers: time and financial constraints, lack of free cessation medications and follow-up, deprioritisation and unclear targets in primary care, lack of knowledge of healthcare professionals, and unclear messaging to patients about available cessation support options. No studies assessed cost-effectiveness.

**Conclusions:**

Some implementation strategy categories increased the rate of smoking status recording and cessation advice provision in primary care. We found some evidence for interventions utilizing financial strategies having a beneficial impact on cessation. Identified barriers to effectiveness should be reduced. More pragmatic approaches are recommended, such as hybrid effectiveness-implementation designs and utilising Multiphase Optimization Strategy methodology.

**Protocol registration:**

PROSPERO:CRD42021246683

**Supplementary Information:**

The online version contains supplementary material available at 10.1186/s12875-023-01981-2.

## Background

Smoking remains one of the leading preventable causes of illness and premature death in the UK [1] and worldwide [2]. Most adult smokers want to quit smoking [3–5] but quit attempts have a low success rate because fewer than a third use evidence-based treatment [3, 5]. For example, the current stop-smoking interventions recommended in the UK are: behavioural support, nicotine replacement therapy (NRT), bupropion, varenicline, and nicotine-containing electronic cigarettes [6]. Healthcare practitioners can trigger and aid quit attempts, increasing cessation likelihood by up to three times [7]. Primary care is suitable for addressing cessation because smokers frequently attend, and it is an opportunistic and trustworthy setting [8]. The World Health Organization (WHO) recommends that “cessation support and treatment is provided in all health care settings and by all health care providers” [9], especially in primary health care systems as this infrastructure already exists in most countries and has a high population coverage. Clinical guidelines recommend addressing patients’ tobacco use by giving “brief advice” to all patients [10]. The first model for this was the ‘5As’ [11, 12] and in some countries now, the ‘3As’ or ‘Very Brief Advice’ (VBA) is recommended [13] (Appendix 1).

Cancer Research UK recently modelled that if GPs intervened at least once a year with all smokers who attended an appointment (referring smokers to a stop smoking service (SSS) and prescribing a cessation medication), national smoking prevalence in 2030 in England would be 2.5% lower than if current rates of brief advice were continued [11]. Despite evidence-based recommendations and guidelines, internationally there is an ‘evidence-practice gap’ in the rates at which healthcare professionals assess tobacco use and offer support in clinical practice in the real-world [14]. A systematic review of primary care physicians in 17 countries found the following average rates: “65% for ‘Ask’, 63% for ‘Advise’, 36% for ‘Assess’, 44% for ‘Assist’, and 22% for ‘Arrange’ [14].

Integrating smoking cessation treatment into routine clinical care and infrastructures is difficult [5]. Implementation science argues that focused efforts are required to facilitate the movement of evidence-based practices (EBP) (e.g., 5As/VBA) into clinical practice because the contexts that the EBP aims to enter are complex and variable [15]. ‘Implementation strategies’ are “methods or techniques used to enhance the adoption, implementation, and sustainability of a clinical program or practice” [16, 17], e.g., “remind clinicians”, “fund and contract for the clinical innovation” [18]. The Expert Recommendations for Implementing Change (ERIC) programme defined 73 distinct ‘implementation strategy’ categories organised into nine implementation strategy domains [18, 19] (Appendix 2).

A recent Cochrane review [20] evaluated randomised and cluster-randomised controlled trials of implementation strategies designed to increase the rate and quality of delivery of the 5As/VBA to adult primary healthcare patients, when delivered in addition to ‘standard’ cessation support or ‘usual care’. Their primary outcome measure was smoking abstinence at long‐term follow‐up (at least 6 months) and their secondary outcomes were practitioner performance in the 5As and quit attempts. They found moderate-certainty evidence for adjunctive counselling (counselling delivered by a health professional other than the primary care physician), free stop-smoking medications, and tailored print materials increasing quit rates. They found no clear evidence for biomedical feedback, provider training, or provider incentives increasing quit rates. For secondary outcomes, they found some evidence that adjunctive counselling increased cessation medication provision, quit attempts, and arranging patient follow‐up by physicians; free stop-smoking medications increased quit attempts; and mixed results for tailored print-materials regarding quit attempts. They found evidence that provider training increased smoking status recording, cessation advice provision, cessation counselling, and providing self-help materials, but mixed results for participants setting a quit date, cessation medication provision, quit attempts, and arranging patient follow‐up. For multi-component interventions, adjunctive counselling combined with free stop-smoking medications, and adjunctive counselling combined with provider smoking cessation training increased quit rates. Combining provider training with flow sheets to aid physician decision-making also increased quit rates; but the results for secondary outcomes for smoking status recording, cessation medication provision, and physicians arranging patient follow‐up were mixed. Lastly, combining provider training with outreach facilitation had no effect on quit rate, recording smoking status, providing cessation medication, or quit attempts; but had some beneficial effect on participants setting a quit date, providing self-help materials, and arranging patient follow-up.

The Cochrane review did not include observational studies; hence the current review focuses on studies which evaluated the impact of implementation strategies enacted without researcher input, in the ‘real-world’, as a national or state-wide policy or change to clinical guidelines. This review complements the Cochrane review and sought to identify differences in the findings which may be due to barriers to implementation in the real-world, to help explain the evidence-practice gap.

### Review questions

The aim of this systematic review was to identify implementation, effectiveness and cost-effectiveness of implementation strategies aiming to increase smoking cessation treatment provision in the primary care setting. Given the evidence-practice-gap, it is important to identify potential facilitators and barriers to implementation. As a secondary outcome, we therefore extracted the proposed facilitators and barriers to effectiveness (qualitative outcomes) from the studies which assessed the effectiveness of implementation strategies on quantitative outcomes.

- RQ1 (Primary): What implementation strategies aiming to increase smoking cessation treatment provision in the primary care setting have been implemented on a national or state-wide scale?
- RQ2 (Primary): Which implementation strategies were effective (practitioner-level and patient-level outcomes) in increasing smoking cessation treatment provision in the primary care setting?
- RQ3 (Secondary): What explanations (perceived facilitators and barriers) have been proposed to explain why certain implementation strategies to increase the provision of smoking cessation treatment in primary care settings were/were not effective?
- RQ4 (Secondary): What is the cost-effectiveness of effective implementation strategies to increase the provision of smoking cessation treatment in primary care settings?

## Methods

### Protocol and registration

The systematic review protocol was registered on PROSPERO on 1 April 2021  (ID: CRD42021246683), it follows the PRISMA statement [21] (Appendix 3).

Amendments made after protocol registration were: interventions were only included if they involved an implementation strategy enacted on a national/state-wide scale; PhD theses were excluded; key contacts and organisations were not contacted to identify publications not retrieved by the search strategy.

### Search strategy

The searches were carried out on 7 April 2021. ‘Smoking’, ‘smoking cessation’ and ‘primary care’ subject headings and key words were used. MEDLINE, EMBASE, CINAHL, PsycINFO, ASSIA Applied Social Sciences Index and Abstracts, Global Health, and Social Policy & Practice were searched for published journal articles. OpenGrey, Social Care Online, and Healthcare Management Information Consortium (HMIC) Database were searched for grey literature. The King’s College London library service and the authors of the related Cochrane review [20] were consulted in developing the search strategy (Full search strategy: Appendix 4).

Forward and backward direct citation tracking was conducted using Web of Science [22]: studies published before 7 April 2021 which cited the included studies, and studies referenced by the included studies, were screened against the inclusion/exclusion criteria.

### Article selection

#### Inclusion/exclusion criteria

##### Participants/population

The target of the intervention(s) was any type of healthcare professional within the primary care setting. ‘Primary care setting’ was defined as family medicine or general medical practice [20]. Excluded are public health interventions delivered outside primary care practices and interventions delivered in dental settings or pharmacies. Studies including the whole practice patient population were included, as well as those which included specific sub-populations in primary care settings (e.g., people with chronic obstructive pulmonary disease (COPD), diabetes, adolescents, pregnant women). Studies were excluded if outcome data could not be extracted exclusively for the primary care setting.

##### Intervention/exposure

Articles were included if they evaluated an ‘implementation strategy’ [16–18] aiming to increase smoking cessation treatment provision in the primary care setting which was implemented on a national or state-wide scale. The focus of this review was specifically on implementation strategies which were implemented nation-wide or state-wide because we were interested in the scalability of implementation strategies. Articles which assessed local-scale (i.e.: ‘non-national’ or ‘non-state-wide’) implementation strategies were excluded.

##### Control

Control could be usual care, any other intervention, or before and after designs. Cross-sectional studies without a comparison/control were excluded.

##### Outcome measures

Articles that assessed any of the primary outcome measures were included in the review.

**Primary outcome measures:**

1. Implementation strategy identification: Description of the implementation strategy was extracted from the article.
2. Implementation strategy effectiveness:

- 2.a. Practitioner-level:
- Practitioner performance in 5As/VBA – definitions used by the original studies were accepted:
**• **Ask (ask patients about smoking at every visit)
• Advise (advise all tobacco users to quit)
• Assess (assess smokers’ willingness to try to quit)
• Assist (assist smokers’ efforts with treatment and referrals, e.g.: ‘discuss medications’, ‘prescribe medications’, ‘set a quit date’, ‘provide counselling’)
• Arrange (arrange follow-up contacts to support cessation efforts)
- 2.b. Patient-level:
• Smokers entering into cessation programmes, facilitated by healthcare professionals in primary care (e.g.: attending smoking cessation clinic or behavioural support appointments; filling prescriptions; calling quit telephone helpline)
• Smokers setting a quit date or quit attempts
• Smoking cessation

**Secondary outcome measures:**

1. Facilitators and barriers to effectiveness: Explanations (perceived facilitators and barriers) offered by the original study authors to explain why certain implementation strategies aiming to increase the provision of smoking cessation treatment in primary care settings were/were not effective.
2. Implementation strategy cost-effectiveness: Any measures relating to cost-effectiveness or economic indicators of the intervention in the study (which may have involved one or multiple implementation strategy categories).

##### Date

Date restrictions regarding publication were not applied.

##### Study design

Non-randomised designs, including comparative observational study designs such as cohort (prospective and retrospective) studies, case–control studies, interrupted time series studies.

##### Language

English.

##### Publication type

Published studies and reports were included. PhD theses, conference abstracts, protocols, reviews, systematic reviews, letters, editorials, commentaries, and studies with only qualitative outcomes were excluded.

### Screening process

The search results were imported into www.cadima.info and duplicates were removed. Articles were screened by BT at two stages (title/abstract, and full text). Reasons for exclusion were documented at the full text level. The PICO checklist used during screening was piloted between BT and a second screener (PP-H). The second screener (PP-H) screened 200 records at the title/abstract screening stage, and 30 records at the full text screening stage. Inconsistencies were discussed and the inclusion and exclusion criteria were clarified by the two screeners and a third reviewer (LB).

### Data extraction

Data from included studies were extracted into a pre-piloted form (Appendix 5). BT performed the data extraction for the 49 included studies, where there were uncertainties about the outcomes, BT consulted LB. Data extraction was performed by BT with oversight from LB. Authors were contacted to provide missing data. Where these data were not provided, they are reported as “missing”.

### Risk of bias assessment

The ROBINS-I (Risk Of Bias In Non-randomized Studies of Interventions) tool was used to evaluate the risk of bias in non-randomised observational studies [23–25]. BT performed the risk of bias assessments. After the first five studies were assessed, LB also assessed these, and BT and LB compared ratings. The risk of bias assessment ratings and justifications are included in Appendix 6.

The tool assesses risk of bias in seven domains [23]:

- Pre-intervention: (1) confounding, (2) selection of participants into the study.
- At intervention: (3) classification of interventions.
- Post-intervention: (4) deviations from intended interventions, (5) missing data, (6) measurement of outcomes, (7) selection of the reported result.

Then an overall risk of bias rating is decided for each study: low, moderate, serious, or critical risk of bias, or no information available [23].

### Synthesis methods

Due to heterogeneity in study populations and outcome measures, a narrative synthesis was used.

Based on the descriptions provided in the included studies, the key aspects of the interventions under investigation were coded to the nine implementation strategy domains and 73 categories developed by the ERIC program [18, 19] (Appendix 2).

Perceived facilitators and barriers, extracted from the studies, were mapped to the determinants in the Consolidated Framework for Implementation Research (CFIR) [26] (Appendix 7).

## Results

### Study selection

**Fig. 1:**
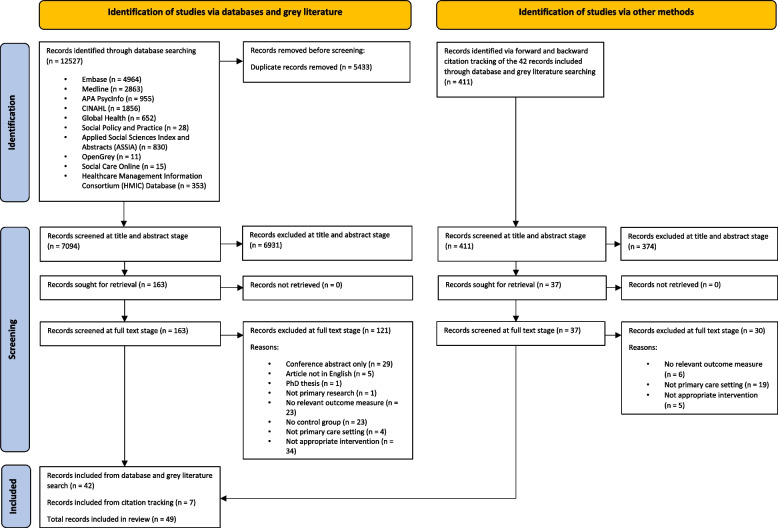
PRISMA flow diagram. PRISMA flow chart showing the number of papers identified through the search strategy and the study selection process

The database search strategy yielded 12,527 records. After de-duplication and screening, 42 studies met the inclusion criteria. Forward and backward direct citation tracking identified an additional seven papers, resulting in 49 papers being included in this review (Fig. 1).

### Study characteristics

Table 1 shows the characteristics of the 49 included studies. Studies were set in the UK (*n* = 23) [27–49], USA (*n* = 13) [50–62], Ireland (*n* = 4) [63–66], the Netherlands (*n* = 3) [67–69], Australia (*n* = 2) [70, 71], Turkey (*n* = 1) [72], Poland (*n* = 1) [73], Finland (*n* = 1) [74], and one [75] compared different policies in the Germany and the UK.

**Table 1:** Study characteristics

First author, year	Location	Implementation strategy category	Study design	Patient population (within primary care setting)	Data source and outcome measure definition
**Domain 5. Train and educate stakeholders**					
** Mullins, 1999** [70]	Victoria, Australia	40. Distribute educational materials	Repeated cross-sectional study Analytical	Aged 16 years and over, smokers 1990: *n* = 624 1992: *n* = 596 1994: *n* = 609 1996: *n* = 563	Data source: Population-based survey of adults in Victoria Outcome measure: Practitioner-level: Each year, smokers were shown a card, and asked, “Which of any of those things has your GP ever said to you?”. Mutually exclusive categories were created by developing a hierarchy of response, and each respondent was coded according to the most appropriate advice he or she had ever been given
** Vasankari, 2011** [74]	Finland	42. Conduct educational meetings	Repeated cross-sectional study Analytical	Aged 16 years and over, respiratory symptoms 1997: *n* = 1,072 patients 2002: *n* = 1,645 patients	Data source: Electronic patient record system of one "medium-sized primary healthcare center in south-west Finland with computerized patient records" Outcome measure: Practitioner-level: "history of smoking", "data on smoking status available"
**Domain 7. Engage consumers**					
** Szatkowski, 2011** [33]	England	54. Prepare patients/consumers to be active participants	Repeated cross-sectional study Interrupted time series analysis (no control)	Aged 16 years and over, smokers 2000 to 2009: *n* = missing	Data source: UK-representative primary care electronic healthcare records, THIN Outcome measure: Practitioner-level: British National Formulary drug codes were used to identify smokers with one or more prescriptions for NRT, bupropion or varenicline recorded in their notes each month. Not enough data to model trends in prescribing of varenicline
** Langley, 2012** [46]	England (and Wales)	56. Use mass media	Repeated cross-sectional study Interrupted time series analysis (no control)	Unspecified January 2002 to June 2009: *n* = missing "Some of the outcome data cover England only, but due to the make-up of the United Kingdom's TV regions, TVRs for Wales cannot be separated from those for England."	Data source: UK-representative primary care electronic healthcare records, THIN Outcome measure: Practitioner-level: "prescribing of NRT"
**Domain 8. Utilize financial strategies**					
** Alageel, 2019** [31]	England	57. Fund and contract for the clinical innovation	Cohort study Interrupted time series analysis (with control)	Aged between 40–74 years Intervention group: had a health check recorded between 1 April 2010 and 31 December 2013 (Read medical codes indicating that a health check or CVD risk assessment was completed) (*n* = 127,891 participants, from 431 general practices in England) "Consistent with the eligibility criteria for the NHS Heath Check, health check participants were excluded if they had diagnoses of ischaemic heart disease, stroke or diabetes, or were treated with antihypertensive drugs or statins before the date of the health check." Matched cohort: matched for age, sex, and general practice, participants who did not receive the check with follow-up data available up to the latest date of 31 March 2017 (*n* = 322,910)	Data source: UK-representative primary care electronic healthcare records, CPRD Outcome measure: Practitioner-level and patient-level: "Read codes relating to smoking and smoking advice." "Product codes indicating prescription of smoking cessation therapy." "Smoking cessation interventions were divided into two categories: referrals to a smoking-cessation advisor or stop smoking clinic and medication (nicotine replacement therapy)."
** Bennett, 2008** [65]	Ireland	57. Fund and contract for the clinical innovation	Cohort study	Patients with diagnosis of coronary heart disease ("patients attending primary care from February 2003 after an acute myocardial infarction (AMI) or coronary intervention, such as percutaneous coronary intervention or coronary artery bypass grafting, which may have been recent or some time ago." 2004, 1-year follow-up cohort: *n* = 7,099 patients, 84.4% had four or five visits over the year 2005, 2-year follow-up cohort: *n* = 4,011 patients, 60.5% had at least eight or nine visits over 2 years	Data source: Primary care electronic medical records. 470 (20%) of all Irish GPs were selected to participate in the programme Outcome measure: Patient-level: "the percentage smoking prevalence was calculated based on an individual having at least one of the following recorded: smoker of one or more cigarettes per day, cigar or pipe smoker." "Absolute change in risk factors between baseline and the 1-year or 2-year follow-up visit was calculated."
** Fitzpatrick, 2011** [66]	Ireland	57. Fund and contract for the clinical innovation	Cohort study	Patients with diagnosis of coronary heart disease (significant proven coronary heart disease (CHD); a history of myocardial infarction (MI), percutaneous coronary intervention or coronary artery bypass graft surgery) 2004, 1-year cohort: *n* = 8,309 patients 2005, 2-year cohort: *n* = 5,431 patients 2006, 3-year cohort: *n* = 3,470 patients 2007, 3.5-year cohort: *n* = 2,078 patients	Data source: Primary care electronic medical records. The programme involved 480 (20%) of general practices Outcome measure: Patient-level: "The percentage smoking was calculated based on an individual having one or more of the following recorded — smoker of one or more cigarettes per day, cigar or pipe." "Absolute changes in risk factors between baseline and follow-up were calculated." "medication prescription" **raw figures not available for smoking cessation medication prescription
** Forster, 2016** [48]	England	57. Fund and contract for the clinical innovation	Cohort study	Aged between 40–74 years Intervention group: had a health check recorded between 1 April 2010 and 31 March 2013, never treated with antihypertensive drugs or statins, and not diagnosed with diabetes, stroke or coronary heart disease before the check (*n* = 91,618 patients) Control group: (n = 182,245 patients), matched controls were identified for 75,123 (82%) of the intervention group	Data source: UK-representative primary care electronic healthcare records, CPRD Outcome measure: Practitioner-level: "The date of each risk factor record was evaluated with reference to the date of the Health Check (the date of the check was the reference date for the cases and their matched controls were also assigned this date of the check). We evaluated risk factor detection, including … the proportion with current smoking recorded…"
** Frijling, 2003** [69]	Netherlands	57. Fund and contract for the clinical innovation	Controlled before-and-after trial	Aged 60 years and over, with high cardiovascular risk (diagnosed with diabetes, hypertension, hypercholesterolaemia, have cardiovascular disease history or family history of coronary heart disease) Intervention group: 420 practices randomly invited from the 800 practices participating in the nationwide project. Response to baseline (October 1998) and post-intervention (September 2000) questionnaire: 316 GPs (84.0%)—returned the shortened version of the post-intervention questionnaires: 37 GPs (11.7%) Control group: 600 practices randomly invited from the 4000 practices which did not participate in the nationwide project. Response to baseline (October 1998) and post-intervention (September 2000) questionnaire: 301 GPs (77.2%)—returned the shortened version of the post-intervention questionnaires: 74 GPs (24.6%)	Data source: GP postal questionnaire Outcome measure: Practitioner-level: "The information was provided by one GP per practice and the same GP for both measurement points." "Assessment of … the following risk factors: … smoking habits." "the GPs were asked whether the minimal contact intervention (MCI) for smoking cessation was used in their practices"
** Pajak, 2010** [73]	Poland	57. Fund and contract for the clinical innovation	Cohort study	Aged between 35–55 years. "Free of cardiovascular disease and with medical documentation going back to at least 1 January 2005". The final examination was conducted in 2007 Active clinics: 33 clinics, *n* = 3,940 patients. *n* = 1,244 patients (31.6%) participated in the PCVDP. Participated in final examination: n = 2,314 patients (58.7%) Control clinics: 33 clinics, *n* = 3,162 patients. Participated in final examination: *n* = 2,107 patients (66.6%)	Data source: Patient healthcare records, patient questionnaire Outcome measure: Practitioner-level: "Information on risk factors (smoking)" in patient healthcare records Patients were interviewed by a trainer interviewer, current smoker patients were asked whether they received "verbal advice or leaflets" regarding tobacco cessation, whether they were "referred to a specialist clinic", whether they received "pharmacotherapy", and whether they discussed "other methods" regarding tobacco cessation Patient-level: Patients reaching "prevention targets" ("not smoking"), but this seems to be 'non-smoking prevalence at final examination'
** Wright, 2018** [71]	Australia	57. Fund and contract for the clinical innovation	Cohort study	Aboriginal and Torres Strait Islander people, aged 15 years and over, "has attended the health service [at least] three times in the past 2 years", 2014–2016 65% of all services that provide national key performance indicator (nKPI) data (152/233) were included: 44 TIS currently funded services and 108 non-TIS-funded services ‘Exposure’ was defined as an organisation that was funded (*n* = 44/152) either directly or indirectly (via consortium arrangements) by the Australian Government’s TIS program 2016: *n* = 81,187 clients accessed TIS-funded services, *n* = 85,098 clients accessed non-TIS-funded services	Data source: Aggregate service-level patient electronic health records; national key performance indicator (nKPI) data Outcome measure: "(1) the number (and proportion) of clients with a smoking status recorded in the health service records; and (2) the number (and proportion) of clients with smoking status recorded as current, ex- and non-smoker." **Timeline of intervention is unclear. TIS program started in 2016. This study uses 2014 as the pre-intervention timepoint, and 6-months into 2016 as the post-intervention/during intervention timepoint
** Bailey, 2016** [54]	Oregon, USA	59. Place innovation on fee for service lists/formularies	Cohort study	Aged between 19–64 years, smokers. "Low-income adults". Pregnant women excluded Intervention group: *n* = 5,935 patients gained Oregon Medicaid coverage between 2008 and 2011 after being uninsured for ≥ 6 months and who maintained this insurance for ≥ 6 months Control group: *n* = 9,371 patients who did not gain Medicaid, patients who were continuously uninsured throughout the 24-month follow-up period and met the current smoker criteria Final study sample included 4,140 matched pairs (*n* = 8,280 patients)	Data source: Primary care electronic healthcare records (Oregon Community Health Information Network, “OCHIN, Inc.”) Outcome measure: Patient-level: "…discrete data field for smoking status, and the OCHIN workflow requires review of tobacco use status at each primary care encounter." "smoking status (i.e., current every day, current some day, former, or never smoker) can be confirmed or modified, and the reviewed or changed date is saved in the EHR. Tobacco cessation medications were abstracted from EHR medication order data." "Our primary outcome was ‘quit’ smoking status after the baseline assessment, coded as a binary yes/no variable. A person was identified as ‘quit’ if baseline smoking status was ‘current every day’ or ‘some day’ and status changed to ‘former smoker’ at a subsequent visit.” Practitioner-level: “We also assessed prevalence of having a smoking cessation medication ordered (yes/no), and analyzed quit smoking status stratified by whether medication was ordered. Medications included bupropion, varenicline, and all nicotine replacement products."
** Bailey, 2020** [60]	United States (multi-state)	59. Place innovation on fee for service lists/formularies	Cohort study	Aged between 19–64 years. Tobacco user. Pregnant women excluded Intervention group: Medicaid-expansion states from 1 January 2014 (California, Hawaii, Maryland, Minnesota, New Mexico, Ohio, Oregon, Rhode Island, Washington, and Wisconsin), *n* = 219 primary care community health centres (CHCs). *n* = 62,164 patients Control group: non-Medicaid-expansion states (Florida, Kansas, Missouri, North Carolina, Texas, and Montana). n = 108 primary care CHCs. n = 31,881 patients States had electronic health records from 1 January 2013. Outcomes assessed 24-months post-expansion: 31 December 2015 Propensity score matched comparison sample: expansion states (*n* = 27,670 patients), non-expansion states (*n* = 27,670 patients)	Data source: Electronic medical records (from primary care community health centres (CHCs)… This study used CHC data from the OCHIN network and the Health Choice Network (HCN)." Outcome measure: Patient-level: "The EHR presents a discrete data field for tobacco use status at each primary care encounter, which can be confirmed, updated, or not reviewed. If confirmed or updated, the date is saved. Our primary outcome was tobacco cessation (“quit”) during the post-period, coded as a binary yes/no variable. … a person was identified as “quit” if the last recorded tobacco-use status during the pre-period indicated that the patient was a current user, and if there was at least one subsequent measurement documented in the post-period that indicated the patient’s status was a “nonuser” (eg, former user, not a current user)." Practitioner-level: "tobacco cessation medications from EHR medication orders: bupropion, varenicline, and all nicotine replacement products"
** Li, 2018** [61]	United States (multi-state)	59. Place innovation on fee for service lists/formularies	Repeated cross-sectional study Descriptive	Aged between 55–80 years. No evidence of lung cancer. Had at least one office visit to a Family Medicine or Internal Medicine provider between 1st January 2010 and 31st December 2016 2010 to 2016: *n* = 1,572,538 patient years	Data source: "Electronic health records (EHR) data from patients in a large community healthcare system located in northern California" Outcome measure: Practitioner-level: "Annual rate of documentation of smoking history is the proportion of patients who had documented smoking history among those with at least one visit in the year."
** Marino, 2016** [62]	Oregon, USA	59. Place innovation on fee for service lists/formularies	Cohort study	Aged between 19–64 years "From a “reservation list” of > 100,000 entries, approximately 30,000 people were randomly selected to apply, and approximately 10,000 gained health insurance (Medicaid) coverage in 2008." In the study, the authors attempted to identify people who gained coverage and patients who were on the reservation list but were not selected to gain coverage. Outcomes assessed 36-months after the selection date (~ 2011) Intervention group: Randomly selected to apply for health insurance coverage: *n *= 4,049 people. Gained health insurance coverage: n = 1,718 people (44% of n = 4,049 actually gained coverage) Control group: Not selected to apply for health insurance coverage: *n* = 6,594 people	Data source: Primary care electronic healthcare record (EHR) data from 49 community health centres (CHCs), OCHIN community health information network (OCHIN, Inc.), in Oregon state Outcome measure: Practitioner-level: "The primary outcomes were whether or not the patient received preventive care services in the post-period: … smoking. Codes were used based on EHR Meaningful Use Stage 1 measures." "Screening for smoking", "assessment of smoking status"
** Miraldo, 2018** [57]	Massachusetts, USA	59. Place innovation on fee for service lists/formularies	Repeated cross-sectional study with control Difference-in-differences (DD) and triple differences (DDD) design	Aged between 18–64 years. Had low income (income below 300% of the federal poverty level) Intervention group: Massachusetts Control group: other New England States (ONES) (Connecticut, New Hampshire, Rhode Island, Maine and Vermont), and higher income groups in Massachusetts who were unaffected by the reform Differences-in-differences (DD) method: Massachusetts vs ONES. "The total sample used for the difference-in-differences (DD) analysis consisted of 131,002 individuals; 39,745 from Massachusetts and 91,257 from ONES." Triple differences (DDD) method: low income and high income patients in Massachusetts vs low income and high income patients in ONES "Massachusetts had the lowest response rate from 2001 to 2007 and for 2010, ranging from 34.6% to 47.7%." In 2008 and 2009, Connecticut had the lowest response rate at 39.8% and 44.23% respectively "The highest response rate was for Vermont in 2001 and from 2003 to 2010, ranging from 52.1% to 60.5%." "In 2002 Maine had the highest response rate at 59.4%."	Data source: Population-based survey of adults in multiple states within the USA. (Behavioural Risk Factor Surveillance System (BRFSS)). "The BRFSS is a state-based survey … involves random-digit dialling (between 2001 and 2010 only landline numbers were included) and a random selection of one adult within that household to participate in a telephone survey." Outcome measure: Patient-level: self-reported: "Current smokers that tried to quit smoking in the past year"
** Parnes, 2002** [58]	Colorado, USA	59. Place innovation on fee for service lists/formularies	Cross-sectional study (with control group) Analytical	Aged between 13–65 years "Colorado Research Network (CaReNet) is a state-wide primary care, practice-based research network founded in 1997 with a particular focus on disadvantaged populations, including rural people, minorities, and the urban poor." *n* = 7 primacy care practices in CaReNet in 1998 and 1999. (*n* = 4 family medicine residency sites, *n* = 2 federally-funded community health centers, n = 1 was clinic for the medically indigent.) CaReNet providers completed NAMCS forms on 2,773 patient encounters of 2,800 eligible visits (99% completion rate) in 1998–1999. n = 1,443 patient visit records remained after excludions. "351 patients in the study sample (24%) were identified as smokers."	Data source: Physician survey (modified version of the 1994 National Ambulatory Medical Care Survey (NAMCS)). "The NAMCS instrument is a physician survey that collects information about an ambulatory visit." "Each CaReNet practice collected data on a total of 400 patient visits in 1-week cycles (100 patients per cycle), quarterly, for 1 year. We used the typical NAMCS protocol of collecting data on every second patient presenting for medical care during the study period." Outcome measure: "the key modification was the addition of “uninsured” in the Expected Source of Payment category. This category included patients who were in 1 of several programs that discount charges on the basis of income, thus covering some of the costs of care." "To identify patients with private insurance, the options “Private/commercial” and “HMO/other prepaid” were combined (“Private/HMO”)." Practitioner-level: "we examined the impact of patient insurance on 2 primary outcomes: (1) patient smoking status, and (2) whether smokers received smoking cessation counseling. Each provider coded smoking status as “Yes,” “No,” or “Unknown.” Only patients with a known smoking status (90% of sample) were included in the present analysis. For those patients coded as smokers, we determined whether providers checked the “Smoking Cessation” box."
** Tilson, 2004** [63]	Ireland	59. Place innovation on fee for service lists/formularies	Repeated cross-sectional study Descriptive	Medical cardholders in Ireland, who are entitled to free prescriptions of certain medicines via the General Medical Services (GMS) scheme In 2002: 29.84% of the population, *n* = 1,168,745 patients	Data source: National prescription database, General Medical Services (GMS) Payments Board prescription database Outcome measure: Practitioner-level: "Using the GMS Payments Board prescription database we conducted a detailed analysis of NRT prescribing (ATC code N07BA)" "the number of monthly prescriptions for each NRT preparation (ATC code N07BA01) and bupropion (ATC code N07BA02)" "Mean dosage, duration of therapy and age/gender distribution of NRT treatment was also obtained." "NRT therapy formulations include gum, patches and inhaled medication."
** Williams, 2004** [64]	Ireland	59. Place innovation on fee for service lists/formularies	Repeated cross-sectional study Descriptive	Medical cardholders in Ireland, who are entitled to free prescriptions of certain medicines via the General Medical Services (GMS) scheme, aged 16 years and over January to December 2001: 31% of the Irish population, *n* = 919,326 patients *n* = 8,166 patients were prescribed Buproprion, *n* = 18,450 patients were prescribed NRT	Data source: National prescription database, General Medical Services (GMS) Payments Board prescription database. the GMS population "cannot be regarded as representative of the general population as socially disadvantaged persons, children and the elderly are over represented, however, they receive about 70% of all medicines prescribed in Irish general practice." Outcome measure: Practitioner-level: "identified those patients who were prescribed Buproprion or NRT"
** Coleman, 2007** [32]	UK	60. Alter incentive/allowance structures	Repeated cross-sectional study Analytical	Aged between 15–75 years. 1990 to 2005 1990: *n* = 776,302 patients 2000: *n* = 1,569,177 patients 2004: *n* = 1,607,782 patients	Data source: UK-representative primary care electronic healthcare records, THIN Outcome measure: Practitioner-level: "smoking status, recorded advice given to stop smoking and prescriptions for nicotine replacement therapy (NRT) or bupropion."
** Dhalwani, 2013** [38]	UK	60. Alter incentive/allowance structures	Repeated cross-sectional study Descriptive	Pregnant women January 2000 to December 2009: *n* = 277,552 pregnancies, *n* = 215,703 women with pregnancies resulting in live births or stillbirths	Data source: UK-representative primary care electronic healthcare records, THIN Outcome measure: Practitioner-level: "Records of maternal smoking status during pregnancy were identified using Read codes. These included codes for current, never, and ex-smoking, codes indicating the type or number of cigarettes smoked, and codes indicating smoking cessation interventions delivered to patients. Women were also considered to be smokers if they had a prescription for a smoking cessation drug (nicotine replacement therapy, bupropion or varenicline) in their medical records during pregnancy." "The prevalence of smoking status recording during pregnancy was calculated for each year from 2000 to 2009 as the number of pregnancies with at least one recording of smoking status during the gestational period divided by the total number of pregnancies delivered in that year." "Since April 2006 the QOF has not required GPs to record the smoking status of patients after the age of 25 years if they have been a never smoker until that age. After 2008, if a patient who once smoked has been recorded as an ex-smoker for three years, GPs need no longer check and update the patient's smoking status records. Therefore, we recalculated the proportion of pregnancies with missing gestational smoking status data to take these rules into account. For women who only had records of being a never smoker up to age 25 and who did not have a record of smoking during a subsequent pregnancy we imputed a never smoking record during gestation. Similarly, for women who had no smoking status records during gestation but who were recorded as ex-smokers for three consecutive years before the conception we imputed an ex-smoking record during gestation. We then recalculated the annual proportion of pregnancies with a recording of smoking status during the gestational period."
** Farley, 2017** [40]	UK	60. Alter incentive/allowance structures	Cohort study	Intervention group: Patients diagnosed with lung, bladder, or upper aerodigestive tract cancer between 1999–2013, had a record of smoking at diagnosis or within 3 years of diagnosis. *n *= 42,112 patients, *n *= 13,449 (32.0%) smoked at diagnosis, *n* = 3,092 (7.3%) had stopped smoking within 3 years of diagnosis Control group: Matched patients with incident CHD diagnosed during the same period as control cases based on year of diagnosis, general practice, and smoking status. *n* = 159,182 patients, *n* = 28,987 (18.2%) smoked at diagnosis, *n* = 6,301 (4.0%) had stopped smoking within 3 years of diagnosis Of these groups, *n* = 12,393 cancer patients were matched to *n* = 12,393 CHD control patients. (*n* = 9,347 patients with lung cancer (86% current smokers), n = 2,050 patients with bladder cancer (90% current smokers), *n* = 996 patients with upper aerodigestive tract cancers (91% current smokers).)	Data source: UK-representative primary care electronic healthcare records, CPRD Outcome measure: Practitioner-level and patient-level: "the proportion of current smokers and recent ex-smokers for whom their general practitioners updated smoking status, advised patients to stop or provided advice on how to do so, and prescribed cessation medication, as well as of patients who quit smoking during the year after diagnosis." "We defined smoking at diagnosis as smoking on the last occasion smoking status was recorded in the 3 years before diagnosis. A recent ex-smoker was defined as someone recorded as smoking within 3 years of diagnosis and subsequently recorded as not smoking on the last occasion before diagnosis."
** Fichera, 2016** [45]	England	60. Alter incentive/allowance structures	Repeated cross-sectional study Regression discontinuity design (with control)	"Sample of individuals reporting at least one condition incentivised by the QOF." 1997 to 2009 "The health conditions recorded in the HSE related to the seven disease areas targeted by the QOF are: cancer, diabetes, other endocrine problems, mental health, stroke, heart attack/angina, hypertension/high blood pressure, bronchitis, asthma, and other respiratory problems." *n* = missing	Data source: Population-based survey of adults in England, "Health Survey for England (HSE) (1997–2009)." "The HSE comprises annual cross-sectional surveys beginning in 1991…. nationally representative of the English adult population with regard to age, gender, geographic area and socio-demographic circumstances. … use 12 years of data from 1997, after which income information was collected." Outcome measure: Practitioner-level: "Smokers are asked whether they have been given smoking cessation advice by a medical practitioner and if so, whether such advice was delivered within the past 12 months. As the smoking cessation variable was not recorded in 2000, 2001 and 2002, we use a multiple imputation procedure to account for missing observations." "The HSE contains information on the medicines that individuals have been prescribed." Patient-level: "In each wave of the survey, respondents are asked whether they smoke and, if so, the average number of cigarettes smoked in a day. Non-smokers are coded as having zero consumption of cigarettes per day."
** Hardy, 2014** [39]	UK	60. Alter incentive/allowance structures	Repeated cross-sectional study Descriptive	Pregnant women, aged 15–49 years at time of giving birth, smokers during pregnancy 2000 to 2009: *n* = 45,296 pregnancies, *n* = 39,781 women (classified as smokers during pregnancy) with pregnancies resulting in live births or stillbirths *n* = 4,826 patients had NRT prescribed during pregnancy for smoking cessation	Data source: UK-representative primary care electronic healthcare records, THIN Outcome measure: Practitioner-level: "Women were defined as smokers if they had a Read code indicating smoking recorded in their medical records or a drug code for nicotine replacement therapy (NRT) during their pregnancy, or, in the absence of recording during pregnancy, if their last recorded Read code in the 27 months prior to pregnancy indicated smoking as defined in more detail previously." "Across the whole study period, annual proportions of pregnant smokers with records of smoking cessation advice were calculated as the number of pregnancies among smokers with recorded smoking cessation advice divided by the total number of pregnancies among smokers who gave birth in that year."
** McGovern, 2008** [43]	Scotland	60. Alter incentive/allowance structures	Repeated cross-sectional study Analytical	Patients with diagnosis of coronary heart disease (CHD), aged 16 years and over Pre-contract (31 March 2004): *n* = 58,406 patients over 16 years had a computer record of CHD, 3.7% of the 1,578,902 registered individuals. n = 48 patients had a computer record of an exception code Post-contract (31 March 2005): *n *= 75,495 of patients over 16 years had a computer record of CHD, 4.9% of the 1,533,802 registered individuals. n = 3,083 patients had a computer record of an exception code	Data source: Primary care electronic healthcare records: "Anonymized retrospective data from all 310 of the 850 Scottish practices who use the general practice administrative software system (GPASS) and who participate in Scottish Programme for Improving Clinical Effectiveness (SPICE) were obtained in November 2005." "previously been shown to be representative of the Scottish population." Outcome measure: Practitioner-level: "recording of smoking status and (where appropriate) provision of smoking cessation advice"
** Millett, 2007** [44]	UK	60. Alter incentive/allowance structures	Cohort study	Patients with diagnosis of Type 1 or Type 2 diabetes (had Read codes for diagnoses of diabetes (C10) or diabetes care (66A)), received repeat prescriptions for diabetic medications or had glycosylated hemoglobin level was greater than 7.5%, aged 18 years and over. Women with gestational diabetes or who received treatment for polycystic ovarian syndrome rather than diabetes were excluded *n* = 32 practices out of 36 practices in the study area agreed to participate *n* = 4,284 patients registered with the 32 practices in both the 2003 and 2005 study periods	Data source: Primary care electronic healthcare records: "Wandsworth Primary Care Trust, located in southwest London, England, has established comprehensive primary care-based diabetes registers." Outcome measure: Practitioner-level: "We examined smoking status and cessation advice based on information recorded on practice computers during the 2003 and 2005 study periods."
** Simpson, 2006** [49]	Scotland	60. Alter incentive/allowance structures	Repeated cross-sectional study Analytical	Patients with diagnosis of transient ischaemic attack or stroke Pre-contract (31 March 2004): *n* = 21,901 patients had a computer record of any stroke or TIA (1.2% of everyone registered with the practices). n = 46 patients had a computer record of an exception code Post-contract (31 March 2005): *n* = 32,401 patients had a computer record of any stroke or TIA (1.8% of everyone registered with the practices). n = 2,565 had a computer record of an exception code	Data source: Primary care electronic healthcare records: "Anonymous retrospective data from all 310 of the 850 Scottish practices that use the General Practice Administrative Software System and that participate in SPICE were obtained in November 2005. These 310 practices were self-selected; however, they have been shown to be representative of all Scottish practices." Outcome measure: "From the accumulated data, we identified everyone who had a computer record of a TIA (read codes G65 to G654, G656 to G65zz) or stroke (including cerebral hemorrhagic; read codes G61 below but not G617, G66, and below) and nonhemorrhagic stroke (read codes G63y0-1, G6760, G6w, G6x, G64, and below) on March 31, 2004 (1 year before introduction of the new contract in April 2004, the “precontract” period in this article) and March 31, 2005 (1 year after introduction of the new contract in April 2004; the “postcontract” period). All registered patients with a recording of stroke before the 2 time points were included in the analyses." Practitioner-level: "… smoking habits (current, ex-smoker, or never smoked) and (where appropriate) provision of smoking cessation advice…"
** Sutton, 2010** [47]	Scotland	60. Alter incentive/allowance structures	Repeated cross-sectional study Analytical	Aged 45 years and over Unit of analysis: each risk factor for each patient in each year. Within a year therefore, there are five observations for each patient. … Patients that are registered with a practice throughout all 6 years appear 30 (5 risk factors * 6 observation years) times." 2000 to 2005: *n* = 9,416,130 observations on 5 five risk factors for *n* = 391,323 individuals in each of up to 6 years	Data source: Primary care electronic healthcare records: Scottish Programme for Improving Clinical Effectiveness in Primary Care (SPICE-PC) data from 315 Scottish practices. "Participation in SPICE-PC is voluntary" "Participation in SPICE-PC was less likely in the most deprived areas and showed some geographical concentration. Compared with non-participants, participating practices had more patients in total (but fewer patients per GP), were more likely to also participate in other voluntary initiatives and achieved 1% more points on average on the 2005/6 QOF. This suggests some caution in extrapolating the results to all Scottish practices. However the differences on each variable are relatively small." Outcome measure: Practitioner-level: recording of risk factor: smoking status. "Practices could also earn additional points for recording that they had offered cessation advice to patients whose current smoking status had been established."
** Szatkowski, 2010** [29]	UK	60. Alter incentive/allowance structures	Repeated cross-sectional study Descriptive	Aged 16 years and over. 1990 to 2006 1990: *n* = 56,595 patients across 103 practices 2006: *n* = 155,359 patients across 399 practices	Data source: UK-representative primary care electronic healthcare records, THIN Outcome measure: Practitioner-level: "We used the proportion of patients having their smoking status recorded within 90 days of registration as a proxy for smoking status being recorded at patient registration."
** Szatkowski, 2011** [28]	England	60. Alter incentive/allowance structures	Repeated cross-sectional study Descriptive	Aged 16 years and over THIN: July 2000: *n* = 1.8 million patients aged 16 + registered with a THIN practice in England July 2009: *n* = 2.0 million patients aged 16 + registered with a THIN practice in England PCT Patient Survey, in England: 2004: *n* = 122,113 completed patient questionnaires, response rate: 47.4% 2005: *n* = 116,939 completed patient questionnaires, response rate: 45.4% 2008: *n* = 69,470 completed patient questionnaires, response rate: 38.3%	(a) Data source: UK-representative primary care electronic healthcare records, THIN (a) Outcome measure: Practitioner-level: "…Read codes documenting the delivery of smoking cessation advice to that patient, and, for each year, the proportion of patients with a recording of cessation advice in the 12 months prior to the index date was calculated." (b) Data source: Representative survey of primary care patients in England (PCT Patient Survey) (b) Outcome measure: Practitioner-level: "postal questionnaire asked whether the respondent had ‘definitely’ or ‘to some extent’ received cessation advice from a health professional (GP or nurse) at their GP surgery within the last 12 months"
** Szatkowski, 2016** [27]	England	60. Alter incentive/allowance structures	Repeated cross-sectional study Interrupted time series analysis (no control)	Aged 16 years and over. 2004 to 2013 *n* = 3,337,881 (SD 81,110) patients aged > 16 years registered in THIN each month, on average *n* = 41,649 (SD 9,082) patients had a record of advice to quit each month, on average *n* = 1,001 (SD 371) patients had a record of referral to the NHS Stop Smoking Service each month, on average *n* = 9,921 (SD 1,851) patients had a prescription for a smoking cessation medication each month, on average	Data source: UK-representative primary care electronic healthcare records, THIN Outcome measure: Practitioner-level: "For each patient, Read Codes were used to identify whether they were advised to quit or referred to the NHS Stop Smoking Service in that month. Multilex drug codes were used to identify whether patients were prescribed a smoking cessation medication (NRT, bupropion, or varenicline) each month."
** Taggar, 2012** [30]	UK	60. Alter incentive/allowance structures	Repeated cross-sectional study Descriptive	Aged over 15 years. 2000 to 2008 2002 (before QOF): *n* = 1,998,631 patients 2004 (at introduction of QOF): *n* = 2,053,840 patients 2008 (after QOF): *n* = 2,149,026 patients	Data source: UK-representative primary care electronic healthcare records, THIN Outcome measure: Practitioner-level: "…patients with a record of smoking status in the last 27 months and patients recorded as smokers with documented cessation advice in the last 15 months; patients were excluded from analysis if they had registered with a practice within the last three months, corresponding to the grace period GPs have to update the records of new patients (which includes the recording of smoking status)."
** Tahrani, 2007** [42]	England	60. Alter incentive/allowance structures	Repeated cross-sectional study Analytical	Patients “on the diabetes register” *n* = 2 Primary Care Trusts (PCTs) in Shropshire, England; made up of 66 practices April 2004: *n* = 15,628 patients on the diabetes register March 2005: *n* = 16,121 patients on the diabetes register March 2006: *n* = 16,867 patients on the diabetes register	Data source: Primary care electronic healthcare records: Pre-intervention measures: National Diabetes Audit, data generated by 65 of the 66 Shropshire GP practices Post-intervention measures: data collected from 66 GP practices in Shropshire at March 2005 and March 2006 Outcome measure: Practitioner-level: "the proportion of patients achieving each quality indicator ("smoking record", "smoking cessation advice") in each practice out of the total number of patients on the diabetes register in that practice"
** Donner-Banzhoff, 1996** [75]	Germany vs UK	65. Use capitated payments	Cross-sectional study (comparing two groups) Analytical	Unspecified. Year unknown *n* = 778 consecutive patients attending for a consultation. "8% of the patients approached declined to take part in the study."	Data source: Patient survey and subsequent patient interview. "A total of 15 family practitioners' surgeries in Germany and the UK that were matched for rural–urban location were included in a cross-sectional survey." Outcome measure: Practitioner-level: Consecutive patients attending for consultation were asked to complete a questionnaire. "They filled in a questionnnaire on sociodemographic data, medication, diagnoses, risk factor concepts, and remembered intervention against smoking. In the following interview, queries arising from the questionnaire could be addressed so as to keep the proportion of missing data low. Patients' records were analyzed for medication, laboratory tests, and previous contacts. During this study, interviews and examinations were performed by one researcher (NDB) in both countries." "Whether a given patient could remember an intervention by his/her physician (or related staff) was defined as the main endpoint of the comparison. An intervention was assumed if the question "Has your family doctor ever talked to you about your smoking?" was answered by "yes" or if questions about possible interventions by doctor or nursing staff were answered in the affirmative. The questionnaire was developed simultaneously in German and English. It was then translated from English into German to correct linguistic ambiguities." Categorisation of 'cessation interventions': 'None' or 'Advice once' or 'Advice several times' or 'Nicotine patch/gum' or 'Other'
**Domain 9. Change infrastructure**					
** Szatkowski, 2021** [36]	England	66. Mandate change	Repeated cross-sectional study Segmented regression analysis, no control	Pregnant women, aged 15–49 years at time of giving birth, smokers during pregnancy 2005 to 2017: *n* = 84,539 pregnancies where the mother was recorded as smoking, this was 24.9% of n = 339,875 all pregnancies	Data source: UK-representative primary care electronic healthcare records, CPRD Outcome measure: "Women were identified as smoking in pregnancy if they had a diagnostic code indicating current smoking, or a prescription for a smoking cessation medication, recorded at least once during gestation." Practitioner-level: "Prescriptions for NRT were identified using relevant Multilex drug codes. Dual NRT was defined as prescription of a long-acting transdermal nicotine patch and a short-acting formulation (eg, gum, lozenge, inhalator, tablet, or spray) on the same day."
** Dhalwani, 2014** [41]	UK	69. Create or change credentialing and/or licensure standards	Repeated cross-sectional study Descriptive	Pregnant women, aged 15–49 years at time of giving birth 2001 to 2012: *n* = 71,685 pregnancies which resulted in live births or still births, where the mother was classified as a smoker during pregnancy, this was 18.5% of *n* = 388,142 of all pregnancies which resulted in live births or stillbirths	Data source: UK-representative primary care electronic healthcare records, THIN Outcome measure: Practitioner-level: "The smoking status of females was determined using Read Codes recorded from 27 months before conception up to the end of pregnancy, based on the recording rules in the GP contract." "Multilex Drug Codes for all NRT formulations available in the UK according to the British National Formulary (BNF) were used for NRT prescriptions." "The use of different forms of NRT (patches, gum, nasal spray, lozenges, sublingual tablets, inhalator cartridges, and combination) was assessed."
** Langley, 2011** [34]	England	69. Create or change credentialing and/or licensure standards	Repeated cross-sectional study Segmented regression analysis (no control)	Aged between 12–17 years. 2002 to 2009 *n* = missing	Data source: UK-representative primary care electronic healthcare records, THIN Outcome measure: Practitioner-level: "rates of prescribing of all NRT products per 100,000 adolescents registered with a THIN practice per month."
** Langley, 2012** [35]	England	69. Create or change credentialing and/or licensure standards	Repeated cross-sectional study Segmented regression analysis (no control)	Aged over 16 years, had diagnosis of cardiovascular disease or stroke. 2002 to 2009 *n* = 88,000 coronary heart disease (CHD) patients each month *n* = 39,000 stroke patients each month	Data source: UK-representative primary care electronic healthcare records, THIN Outcome measure: Practitioner-level: "the number of patients per 100,000 with CHD and stroke who received a prescription for NRT each month." Extracted data on prescribing of NRT, varenicline and bupropion to CHD and stroke patients
** Li, 2020** [55]	United States (multi-state)	69. Create or change credentialing and/or licensure standards	Repeated cross-sectional study Analytical	Aged between 55–80 years, smokers, no evidence of lung cancer. 2010 to 2017 *n* = 12,678 (63.8% of *n* = 19,862) current smokers were included in the analysis, whose eligibility for LDCT-LCS could be determined	Data source: Electronic healthcare records: from a "large healthcare system in northern California" Outcome measure: Practitioner-level: "Three types of smoking-cessation interventions (i.e., formal in-visit smoking-cessation counseling, informal smoking-cessation counseling or referrals to smoking-cessation programs, and medication orders for pharmacotherapy) were considered. … Keyword searches included but were not limited to, smoking cessation and tobacco counseling in the procedure description. Sessions of 3 − 10 min or > 10 min (e.g., billing codes: 99,406, G0375, G0376; 99,407, G0436, G0437, etc.) were classified as formal in-visit smoking-cessation counseling. Smoking-cessation counseling < 3 min is not separately billed; such unbilled in-visit smoking-cessation counseling, along with referrals for internal free smoking-cessation programs, are categorized as informal smoking-cessation counseling or referrals to smoking-cessation programs. Pharmacotherapy using smoking deterrents was identified by a prescription order for smoking-cessation medication, (e.g., bupropion HCl, varenicline tartrate, nicotine polacrilex, etc.)." "cigarettes smoked per day"
** Thorndike, 2007** [53]	United States (multi-state)	69. Create or change credentialing and/or licensure standards	Repeated cross-sectional study Analytical	Aged 18 years and over 1994 to 1996: *n* = 84,104 adult patient visits to 4,118 physicians. Physician response rate: 71% 2001 to 2003: *n* = 58,991 adult patient visits to 2,902 physicians. Physician response rate: 67% Difference between physician response rate is statistically significant (*p* = 0.001)	Data source: Physician survey, "The National Ambulatory Medical Care Survey (NAMCS) is an ongoing annual survey of US office-based physicians conducted by the National Center for Health Statistics." "We compared pooled data from the 1994–1996 NAMCSs with data from the 2001–2003 surveys. … We were unable to examine these outcomes for the years 1997–2000 because the smoking status item was not included on the NAMCS in those years." "Each participating physician completes a 1-page encounter form for each systematically sampled ambulatory care visit during a randomly assigned week." Outcome measure: Practitioner-level: "(1) Physicians identified a patient’s smoking status by answering the question, “Does patient use tobacco?” Smoking status was considered identified if the answer was “yes” or “no”; responses of “unknown” or left blank were considered not identified. (2) Physicians recorded smoking counseling by checking the “Tobacco use/exposure” box under “Counseling/Education.” (3) Prescription and nonprescription medications were recorded on the survey form under “Medications.” All adult patient visits were included in the analysis of smoking status. Analyses of smoking counseling and smoking medications were restricted to visits by patients identified was smokers Because bupropion is also used to treat depression, we excluded bupropion prescriptions prior to 1997, the year it was approved for smoking cessation."
** Peterson, 2016** [52]	United States (multi-state)	71. Change accreditation or membership requirements	Repeated cross-sectional study Analytical	Patients “with hypertension” *n* = 7,319 completed hypertension Performance in Practice Modules (PPMs) completed between 2006 and 2013, reflecting quality measures for between 80,000 and 160,000 patients, completed by eligible physicians (residing in the United States). "In 7.8% of the PPMs, physicians selected smoking cessation for improvement."	Data source: "We analyzed data from all hypertension Performance in Practice Modules (PPMs) completed from July 2006 to 2013." Patient health records: "diplomates gather quality measures from at least 10 patients with hypertension". Patient questionnaire: "patients complete a questionnaire" Outcome measure: "The PPM structure is based on a Plan-Do- Study-Act (PDSA) cycle. First, the physician, or assigned clinical staff, gathers data on 10 patients with hypertension from the chart and the corresponding patient survey data, and enters them on templates in the web-based PPM. [quality improvement exercise]…. After the physician implements their chosen interventions, collection of chart and survey data from the next 10 patients they see with a diagnosis of hypertension is repeated. After completion of data entry for this set of patients, the physician is provided with pre- and post-intervention comparisons as well as comparisons to the mean quality scores for all physicians who have previously completed the PPM." Practitioner-level: Patient records (physician-reported): Whether "smoking cessation counseling" was provided Patients complete a questionnaire that includes: "(6) for smokers, whether your doctor asked about quitting"
** Shi, 2017** [59]	United States (multi-state)	71. Change accreditation or membership requirements	Cross-sectional study (with control group) Analytical	Unspecified, aged 18 years and over, low income. 2012 *n* = 539 health centres (HCs) achieved 'Patient-centered medical home' (PCMH) recognition status *n* = 548 HCs did not achieve PCMH recognition status	Data source: Provider survey/electronic records (Health Resources and Services Administration [HRSA] 2012 Uniform Data System (UDS) + HRSA’s Patient-Centered Medical/Health Home Initiative) Outcome measure: Practitioner-level: "percent of adults (18 years or older) assessed for tobacco use". "percent of adults (18 years or older) who were known tobacco users that received tobacco cessation counseling and/or pharmacologic intervention."
** Van Doorn-Klomberg, 2014** [68]	Netherlands	71. Change accreditation or membership requirements	Cohort study	Patients with diagnosis of diabetes mellitus, chronic obstructive pulmonary disease (COPD) or cardiovascular disease (CVD). 2006 to 2011 Matched sample: 1st cohort: *n* = 69 practices. 2006–2008: *n* = 4,629 average number of patients per practice. 2009–2011: *n* = 4,808 average number of patients per practice Matched sample: 2nd cohort: *n* = 69 practices. 2009–2011: *n* = 4,830 average number of patients per practice	Data source: Primary care electronic healthcare records from Dutch primary care practices that participated in the accreditation program of the Dutch College of General Practitioners between 2006 and 2011 Outcome measure: Patient-level: Patients with COPD and Patients with CVD: "Percentage of patients that smoke" Practitioner-level: "Percentage of patients with a known smoke status", "Percentage of patients that smoke with a stop smoking advice"
**Multiple domains**					
** Akman, 2017** [72]	Turkey	Domain 8 65. Use capitated payments AND Domain 9 66. Mandate change, 67. Change record systems, 71. Change accreditation or membership requirements	Repeated cross-sectional study Analytical	Unspecified patient population 1993: *n* = 199 primary care doctors (response rate: 50%), "doctors working in inner city and urban areas were over-represented" 2012: *n* = 299 primary care doctors (response rate: 42.9%)	Data source: 1993: Primary care doctor survey: 1993 European GP Task Profile study. "In 1993, the study sample included a random sample of PCDs in 10 preselected provinces out of all 74 provinces in Turkey" 2012: Primary care doctor survey: 2012 Quality and Costs of Primary Care in Europe (QUALICOPC) study. "In 2012, data was collected from seven provinces of Turkey. Selection of provinces was based on the year the FD scheme was introduced, and the geographical distribution within the country. A quota of 10% per region was applied for all PCDs with family medicine specialist qualifications working in the region." Outcome measure: Practitioner-level: self-reported proportion of "primary care doctors who are usually or almost always involved in given preventive care service (smoking counselling during outpatient clinic)". "The questions in the 1993 survey on GP service profiles were repeated in 2012 with the purpose of comparing general practice between the two time points."
** Bailey, 2017** [50]	Oregon, USA	Domain 8 60. Alter incentive/allowance structures AND Domain 9 67. Change record systems	Repeated cross-sectional study Analytical	Aged 18 years and over, excludes pregnant patients. "Most are uninsured or Medicaid recipients and have disproportionately high rate of smoking compared with patients seen in private primary care clinics". 2010 to 2014 2010: *n* = 55,398 patients 2012: *n* = 60,610 patients 2014: *n* = 66,712 patients	Data source: Primary care electronic healthcare records (Oregon Community Health Information Network, “OCHIN, Inc.”): 26 Oregon community health centers (CHCs))(federally qualified heath centers that are subsidized to serve low-income and vulnerable populations) that were using OCHIN’s EHR before 1 July 2009 were extracted Outcome measure: Practitioner-level and patient-level: "The denominator for smoking status assessment included all study patients within a measurement year; the denominator for all other outcomes included patients identified as smokers within a measurement year." (1) "Smoking status was considered to be assessed if changes were made to the discrete data field with drop-down options for smoking status (located in both the vital signs and social history in all study years), if the button was selected to confirm that smoking status was reviewed, or if a status was captured via NLP in the free-text notes within the measurement year (2) A patient was identified as a current smoker if smoking status in the discrete data field was “current every day,” “current some day,” “smoker, current status unknown,” “heavy tobacco smoker,” or “light tobacco smoker.”" (3) "Receipt of counseling was deemed “yes” if the discrete field, “counseling given,” was coded as “yes,” if identified by standard procedure codes for smoking-cessation counseling or an internal OCHIN Epic code for counseling referral, or if any statements in the free-text fields about smoking and cessation (e.g., goals, triggers, efforts) were identified." (4) "Smoking-cessation medication orders (bupropion, varenicline, and all nicotine-replacement therapy products) were extracted from the medication orders list (5) “medications ordered or discussed”: included orders or any discussion of cessation medications as captured in the free text via NLP
** Fortmann, 2020** [56]	United States (multi-state)	Domain 8 60. Alter incentive/allowance structures AND Domain 9 71. Change accreditation or membership requirements	Cohort study Interrupted time series analysis (no control)	Aged 18 years and over. "72% of patients were members of ethnic and racial minority groups and 73% reported incomes below the Federal Poverty Level (FPL)." 2006–2013 *n* = 9 US states, 15 community health centres (CHCs), 706,840 patients. (Average CHC size: 4,700 to 67,000 patients.)	Data source: Electronic healthcare records (Community Health Applied Research Network (CHARN)—data from 15 community health centres, across 9 US states) Outcome measure: Practitioner-level: "structured EMR data (not free text) on smoking status and patient characteristics from the 15 CHCs with smoking data beginning either in 2006 or in the earliest year in which data were recorded." "Overall rates of documentation were assessed for each year from 2006 to 2013 at the clinic level (the denominator increased as clinics were added to the database)." "Smoking status was recorded as current, former, never, or unknown/missing. The EMRs in this study carried forward smoking status from previous visits to inform clinical staff of prior smoking status, which could then be reviewed and changed if necessary. If the smoking status was unchanged, this was often not specifically noted. …if no smoking status was recorded in a given year, status was set as that of the last recorded value. Thus, missing/unknown smoking status indicated that providers had never recorded smoking status for a given individual."
** Langley, 2011** [37]	England	Domain 8 59. Place innovation on fee for service lists/formularies AND Domain 9 69. Create or change credentialing and/or licensure standards	Repeated cross-sectional study Interrupted time series analysis (no control)	Unspecified, "all primary care patients registered". 2000–2009 *n* = missing. "Prescribing of varenicline increased most markedly in July 2007, growing to around 100 prescriptions per 100,000 population, and remained around this higher rate for the rest of the study period."	Data source: UK-representative primary care electronic healthcare records, THIN Outcome measure: Practitioner-level: "monthly rates of general practice prescribing for each of NRT, bupropion and varenicline and all smoking cessation medications combined."
** Mullins, 2009** [51]	Delaware, USA	Domain 5 40. Distribute educational materials 42. Conduct educational meetings AND Domain 7 54. Prepare patients/consumers to be active participants	Repeated cross-sectional study Analytical	Unspecified, "all [primary care] patients". "Patients without a recorded smoking history were excluded." 2006 to 2008 Pre-intervention group (office visit between 1 July 2006 and 1 January 2007): *n* = 922 patients Post-intervention group (office visit between 1 July 2007 and 1 January 2008): *n* = 3,154 patients	Data source: Primary care electronic healthcare records from Family Medicine Center of Christiana Care Health System. "… suburban outpatient office in Wilmington, Delaware", USA Outcome measure: Practitioner-level and patient-level: "The number of patients recorded as current smokers and the number of patients counseled to quit by their physician…" "Smokers were defined as patients who had an EMR flow sheet value for “smoking status” that read “current.” Patients were defined as nonsmokers if smoking status flow sheet values were “quit,” “never,” or if no value was recorded "Tobacco cessation counselling", "patient had at some time been counseled to quit smoking by their provider": Patients were defined as having been counseled to quit smoking if the flow sheet value for “advised to quit” was ever recorded as “yes.”" "The inquiry to determine the preintervention group was “Find Patients where Home Location is 'FMC' AND Date of Last Office Visit is on or after '07/01/2006' AND Date of Last Office Visit is before '01/01/2007' AND SMOK STATUS (any entry) contains 'current' AND SMOK ADVICE (last entry) contains 'yes'.” The inquiry to determine the postintervention group was the same, with visits on or after July 1, 2007, and before January 1, 2008."
** Verbiest, 2013** [67]	Netherlands	Domain 8 59. Place innovation on fee for service lists/formularies AND Domain 9 69. Create or change credentialing and/or licensure standards	Repeated cross-sectional study Interrupted time series analysis (no control) (of three nation-wide representative databases)	Unspecified, adults (aged 15 years and over) Netherlands Information Network of Primary Care (LINH): representative sample of 84 general practices with approximately 350,000 listed patients. 2001 to 2011 Dutch Foundation for Pharmaceutical Statistics (SFK): representative panel of 95% of Dutch community pharmacies. 2001 to 2012 Dutch Continuous Survey of Smoking Habits (DCSSH): representative of Dutch adult population (15 years and older). 2001 to 2012	(a) Data source: Nation-wide general practice electronic health records (Netherlands Information Network of Primary Care (LINH)). "The characteristics of the study population (GPs and patients) are comparable with the general Dutch population in terms of age and gender." (a) Outcome measure: Practitioner-level: "number of quarterly prescribed stop-smoking medications in general practice… "prescriptions of NRT, varenicline and bupropion in the period 2001–2011 and calculated prescription rates per 1000 smokers. …. The number of smokers was based on the total population and smoking prevalence." (b) Data source: Nation-wide prescription database (Dutch Foundation for Pharmaceutical Statistics (SFK)). "The SFK gathers data from a representative panel of 95% of Dutch community pharmacies. Data were extrapolated to nation-wide figures." (b) Outcome measure: Practitioner-level: "prescriptions of stop-smoking medication dispensed in out-patient pharmacies". "dispensations of NRT, varenicline and bupropion in the period 2001–2012 and calculated dispensed rates per 1000 smokers." For bupropion: **"primary care prescriptions (a) in this study represent the total number of prescriptions for both depression and quit smoking and the dispensed items (b) represent only stop-smoking medication" For varenicline: "We did not assess the impact of the GP guideline introduction on the number of primary care prescriptions and dispensed prescriptions of varenicline because this pharmaceutical was introduced in the Netherlands around the same time as the GP guideline (December 2006)." "we only assessed the immediate effect of the introduction and abolition of the insurance coverage in (dispensed) prescriptions and smoking prevalence, as we lacked sufficient time-points to estimate a change in trend." (c) Data source: Population-based survey of adults in the Netherlands (Dutch Continuous Survey of Smoking Habits (DCSSH)). "The DCSSH assesses smoking behaviour of the Dutch adult population (15 years and older)." Representative; weightings based on gender, age, education level, socioeconomic status, the province in which they lived, and their family and community size (c) Outcome measure: Patient-level: "Smoking prevalence (2001–2012) was assessed by asking participants ‘Do you (ever) smoke?’."

Table summarising the characteristics of the studies included in this systematic review. The included studies are ordered by implementation strategy domain (5, 7, 8 and 9 and ‘Multiple domains’). Within the domains, the studies are ordered by implementation strategy category then alphabetically by first author surname

(Wright, 2018) [71] was excluded from narrative synthesis as it was at critical risk, but it is included in this table

Thirteen were cohort studies [31, 40, 44, 48, 54, 56, 60, 62, 65, 66, 68, 71, 73]. One was a controlled before-and-after study [69]. Three were cross-sectional with a comparator [58, 59, 75]. The other 32 studies were repeated cross-sectional studies. Ten of these used advanced analytical techniques: interrupted time series design [27, 33, 37, 46, 67], segmented regression design [34–36], regression discontinuity design [45], difference-in-differences and triple differences design [57]. Thirteen of the repeated cross-sectional studies tested for statistical significance between pre- and post-intervention measurements [32, 42, 43, 47, 49–51, 53, 55, 70, 72, 74], and nine only described the pre- and post-intervention measures [28–30, 38, 39, 41, 61, 63, 64].

### Risk of bias

One study [71] had ‘critical’ risk of bias and was not included in the narrative synthesis as per Cochrane guidance [25]. Twenty-four studies [28–30, 32, 36, 38, 39, 41–44, 49, 51, 52, 55, 61, 63, 64, 66, 69, 70, 72, 74, 75] had ‘serious’ risk of bias, predominantly due to receiving a poor rating for the ‘bias due to confounding’ domain. Twenty studies [27, 33–35, 37, 45–47, 50, 53, 54, 56–60, 65, 67, 68, 73] had ‘moderate’ risk of bias; these had more sophisticated study designs which controlled for time-varying confounders but did not have a pre-specified/pre-registered analysis plan, scoring a poor rating for the ‘bias in selection of reported result’ domain. Four studies [31, 40, 48, 62] were at ‘low’ risk of bias – these were cohort studies which controlled for various confounders and had pre-specified analysis plans (Appendix 6).

### RQ1: Implementation strategies that were implemented

**Fig. 2:**
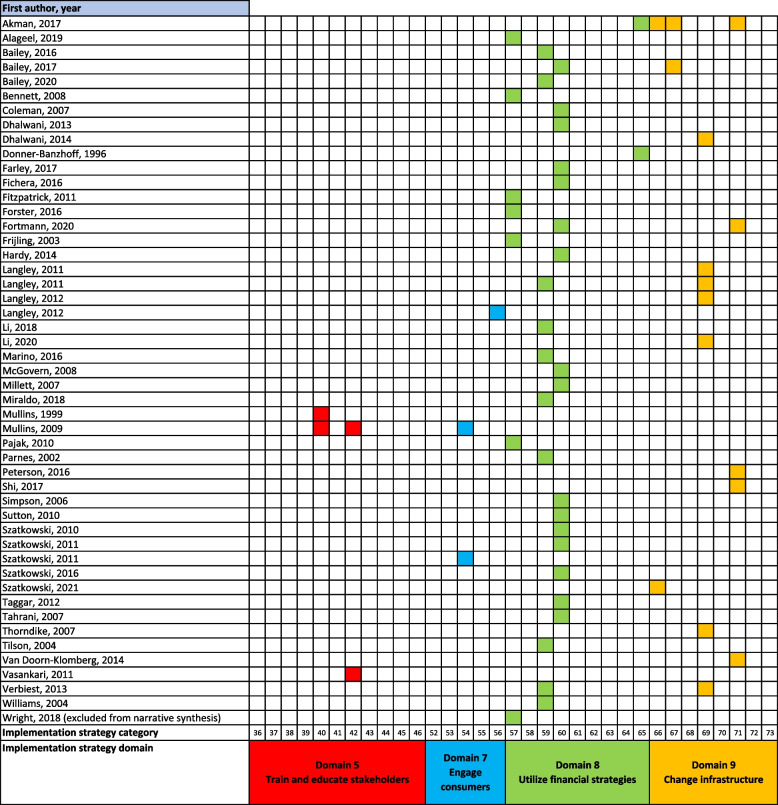
Implementation strategy categories identified in the included studies. The interventions in the 49 included studies were coded to the implementation strategy domains (1 to 9) and categories (1 to 73) developed by the Expert Recommendations for Implementing Change (ERIC) program [18, 19]. Each column represents one of the 73 implementation strategy categories. A shaded cell indicates the specific strategy that the intervention under investigation in the study involved. Only the four domains that were identified are displayed in this figure. The other domains were not included in any of the studies: Use of evaluative and iterative strategies (Domain 1), Provide interactive assistance (Domain 2), Adapt and tailor to context (Domain 3), Develop stakeholder inter-relationships (Domain 4), Support clinicians (Domain 6). (Wright, 2018) [71] was excluded from narrative synthesis as it was at critical risk of bias

Interventions in six studies [37, 50, 51, 56, 67, 72] used multiple implementation strategies; interventions in the other 42 studies used one key implementation strategy category only (Fig. 2). We did not identify studies for all possible implementation strategy domains and categories which are outlined in the list developed by the ERIC program [18, 19] (Appendix 2). The domains in which implementation strategies were identified were ‘Utilize financial strategies’ (Domain 8., 34 studies), ‘Change infrastructure’ (Domain 9., 14 studies), ‘Train and educate stakeholders’ (Domain 5., three studies), and ‘Engage consumers’ (Domain 7., three studies). More details of the implementation strategy domains and categories are given in Table 2 and summarised below when discussing outcomes for RQ2 and RQ3.

**Table 2:** Results

First author, year	Location	Implementation strategy category	Intervention	Practitioner-level outcomes			Patient-level outcomes		Perceived facilitators and barriers	Risk of bias
				**Recording smoking status**	**Providing cessation advice**	**Prescribing cessation medication**	**Quit attempts**	**Cessation**		
**Domain 5. Train and educate stakeholders**										
** Mullins, 1999** [70]	Victoria, Australia	40. Distribute educational materials	Simple intervention: GPs mailed a pack containing: information letter for GPs, self-help booklet ('The Can Quit Book') to give to patients, plastic stand for GPs' office/waiting room	**0**	** + **				**Facilitators:** Intervention characteristics: complexity (the intervention was simple and acceptable: survey found that 95% of primary care physicians could recall receiving copies of The Can Quit Book and most physicians reported giving them to patients) Outer setting: external policies and incentives (GPs may have been affected by smoking cessation articles in medical journals and medical magazines, the RACGP’s Guidelines for Preventive Activities in General Practice, societal changes of embracing anti-smoking advice) **Barriers:** Outer setting: cosmopolitanism (lack of appropriate/easy referral system to effective cessation programs or products)	Serious
** Mullins, 2009** [51]	Delaware, USA	Domain 5 40. Distribute educational materials 42. Conduct educational meetings AND Domain 7 54. Prepare patients/consumers to be active participants	‘Ask and Act program’ Program contains: (i) educational component for physicians (free patient materials for offices, continuing medical education programs for physicians and allied health professionals, and information on evidence-based interventions), and (ii) free patient materials which engage patients (patient materials include pre-printed prescription pads with tips on how to quit, brochures, and laminated quitline referral cards. Metal lapel pins and wall posters act as visual cues to encourage patients to ask their family physician for help, and a guide to tobacco cessation group visits details how practices can organize and bill for counselling sessions)		** + **			** + **	**Facilitators:** Inner setting: readiness for implementation: (iii) access to knowledge and information (physicians reported that they felt more comfortable with smoking cessation counselling and billing for this intervention, and that they were more likely to counsel their patients after hearing the presentation)	Serious
** Vasankari, 2011** [74]	Finland	42. Conduct educational meetings	Finnish ‘National Programme for Chronic Bronchitis and COPD 1998–2007’: training events organised in hospitals and primary health care centres, covering topics: COPD as a disease, diagnosis of COPD (spirometry), smoking cessation and treatment of COPD	** + **					**Facilitators:** Outer setting: external policies and incentives (anti-smoking work and legislation on the national level, increased improvements in the national level of spirometry and knowledge of smoking habits of COPD patients)	Serious
**Domain 7. Engage consumers**										
** Mullins, 2009** [51]	Delaware, USA	Domain 5 40. Distribute educational materials 42. Conduct educational meetings AND Domain 7 54. Prepare patients/consumers to be active participants	‘Ask and Act program’ Program contains: (i) educational component for physicians (free patient materials for offices, continuing medical education programs for physicians and allied health professionals, and information on evidence-based interventions), and (ii) free patient materials which engage patients (patient materials include pre-printed prescription pads with tips on how to quit, brochures, and laminated quitline referral cards. Metal lapel pins and wall posters act as visual cues to encourage patients to ask their family physician for help, and a guide to tobacco cessation group visits details how practices can organize and bill for counselling sessions)		** + **			** + **	**Facilitators:** Inner setting: readiness for implementation: (iii) access to knowledge and information (physicians reported that they felt more comfortable with smoking cessation counselling and billing for this intervention, and that they were more likely to counsel their patients after hearing the presentation)	Serious
** Szatkowski, 2011** [33]	England	54. Prepare patients/consumers to be active participants	Introduction of smoke-free legislation			** + / 0**			**Barriers:** Outer setting: external policies and incentives (contextual factors and social norms continue to influence smoking behaviour: the provision of outdoor facilities for smoking, spending time with smoking friends) Implementation process: executing (cessation support could have been advertised in the months after the smoke-free legislation was enacted)	Moderate
** Langley, 2012** [46]	England (and Wales)	56. Use mass media	Anti-tobacco mass media advertising, and pharmaceutical company-funded smoking cessation medication advertising			**0**			**Barriers:** Implementation process: executing (effect of mass media campaign seems to be restricted to the month of the campaign, suggesting that campaigns need to be sustained over time; the messages of the mass media campaigns could be improved: greater focus on encouraging supported quit attempts, encouraging smokers to seek advice and medication from their GP)	Moderate
**Domain 8. Utilize financial strategies**										
** Alageel, 2019** [31]	England	57. Fund and contract for the clinical innovation	NHS Health Check program (primary prevention of cardiovascular disease and related disorders)		** + **	** + **		** + **	**Barriers:** Characteristics of individuals: knowledge and beliefs about the intervention (lower uptake of health checks among patients at greatest risk of cardiovascular disease) Characteristics of individuals: knowledge and beliefs about the intervention (physicians have doubts about the effectiveness of the EBP, physicians lack guidance on how to implement risk management interventions which follow after risk factor detection) Inner setting: structural characteristics (delivery of EBP is restricted by lack of time and follow-up in primary care)	Low
** Bennett, 2008** [65]	Ireland	57. Fund and contract for the clinical innovation	Heartwatch (secondary prevention of cardiovascular disease)					** + **	**Barriers:** Outer setting: cosmopolitanism (further improvements may be achieved through improved linkages to community-based programmes and support) Outer setting: patient needs and resources (further improvements may be achieved through attention to improving body weight, exercise levels and glucose metabolism)	Moderate
** Fitzpatrick, 2011** [66]	Ireland	57. Fund and contract for the clinical innovation	Heartwatch (secondary prevention of cardiovascular disease)					** + **	**Facilitators:** Inner setting: implementation climate: (ii) compatibility (the effect of the intervention is likely to be additive, to the effect from secondary prevention interventions that already exist in primary care) **Barriers:** Inner setting: structural characteristics (delivery of EBP is restricted by lack of time and follow-up in primary care)	Serious
** Forster, 2016** [48]	England	57. Fund and contract for the clinical innovation	NHS Health Check program (primary prevention of cardiovascular disease and related disorders)	** + **					**Barriers:** Characteristics of individuals: knowledge and beliefs about the intervention (lower uptake of health checks among patients who are smokers)	Low
** Frijling, 2003** [69]	Netherlands	57. Fund and contract for the clinical innovation	Cardiovascular disease (secondary) prevention program		** + **				**Barriers:** Inner setting: readiness for implementation: (ii) available resources (GPs reported time constraints and insufficient financial recompense as a barrier to change, extra resources and personnel will be needed, GPs' current workload needs to be reduced) Outer setting: patient needs and resources (multi-faceted interventions are more effective)	Serious
** Pajak, 2010** [73]	Poland	57. Fund and contract for the clinical innovation	Health Check Program of cardiovascular disease prevention	** + **	**0**	**0**		**0**	**Barriers:** Inner setting: readiness for implementation: (iii) access to knowledge and information (the intervention should be enriched with well-designed structured intervention) Characteristics of individuals: knowledge and beliefs about the intervention (less than 50% of family physicians felt competent to deliver smoking cessation interventions, primary care physicians have been shown to inadequate knowledge and to be not fully aware as to the efficacy of intervention on risk factors) Inner setting: structural characteristics (primary care physicians have been shown to have time limitations) **Facilitators:** Inner setting: implementation climate: (iii) relative priority (over 90% of family physicians felt that health promotion should be a part of their daily work) Inner setting: readiness for implementation: (ii) available resources (over 90% of family physicians had educational materials in their waiting rooms)	Moderate
** Bailey, 2016** [54]	Oregon, USA	59. Place innovation on fee for service lists/formularies	Increasing access to health insurance coverage which included smoking cessation treatment			** + **		** + **	**Facilitators:** Inner setting: structural characteristics (increased access to consultations and follow-up consultations in primary care, increased access to cessation medications) Outer setting: cosmopolitanism (increased access to smoking cessation counselling or referral for such services)	Moderate
** Bailey, 2020** [60]	United States (multi-state)	59. Place innovation on fee for service lists/formularies	Increasing access to health insurance coverage which included smoking cessation treatment			** + **		** + **	**Facilitators:** Inner setting: structural characteristics (increased access to consultations in primary care, increased access to cessation medications) Outer setting: cosmopolitanism (increased access to smoking cessation counselling or referral for such services)	Moderate
** Langley, 2011** [37]	England	Domain 8 59. Place innovation on fee for service lists/formularies AND Domain 9 69. Create or change credentialing and/or licensure standards	(i) Introduction of a new cessation medication (varenicline) onto a country’s prescription scheme, December 2006 (ii) Introduction of NICE guideline for varenicline, July 2007			**(i) + / 0** **(ii) + / 0**			**Facilitators:** Inner setting: readiness for implementation: (iii) access to knowledge and information (measures to increase physicians' confidence in the effectiveness and safety of the medication) Characteristics of individuals: knowledge and beliefs about the intervention (raising awareness of varenicline amongst smokers)	Moderate
** Li, 2018** [61]	United States (multi-state)	59. Place innovation on fee for service lists/formularies	Low-dose computed tomography for lung cancer screening (LDCT-LCS) became a Medicare-covered preventive service	** + **					**Barriers:** Inner setting: readiness for implementation: (ii) available resources (lack of available staff time and financial factors) Intervention characteristics: complexity (information in new guidelines was complex)	Serious
** Marino, 2016** [62]	Oregon, USA	59. Place innovation on fee for service lists/formularies	Increasing access to health insurance coverage which included smoking cessation treatment	** + **					**Facilitators:** Inner setting: structural characteristics (increased access to primary care office visits)	Low
** Miraldo, 2018** [57]	Massachusetts, USA	59. Place innovation on fee for service lists/formularies	Increasing access to health insurance coverage which included smoking cessation treatment				**0**		**Barriers:** Inner setting: structural characteristics (require an extensive amount of physician time) Inner setting: implementation climate: (iii) relative priority (some physicians are not inclined to working with behavioural interventions and perceive risk reduction as something beyond their direct responsibility) Characteristics of individuals: knowledge and beliefs about the intervention (differences across race/ethnic groups also suggest the need to tailor health interventions for multiple races, ethnicities and cultures) **Facilitators:** Outer setting: external policies and incentives (methods for encouraging healthy behaviour, coordinating care of chronic diseases) Implementation process: reflecting and evaluating (multifaceted approaches to implementation, with a combination of activities such as audit and feedback and active education)	Moderate
** Parnes, 2002** [58]	Colorado, USA	59. Place innovation on fee for service lists/formularies	Health insurance types: uninsured vs Medicaid insured vs private/health maintenance organization (HMO) insured		** + **				**Barriers:** Inner setting: structural characteristics (lack of access to cessation resources/treatment) Inner setting: structural characteristics (competing demands on physicians' time) Characteristics of individuals: other personal attributes (studies have documented a lower quality of care for Medicaid and uninsured patients with chronic diseases)	Moderate
** Tilson, 2004** [63]	Ireland	59. Place innovation on fee for service lists/formularies	Introduction of a new cessation medication (NRT) onto a country’s prescription scheme			** + / 0**			**Barriers:** Inner setting: structural characteristics (organisational issues) Inner setting: structural characteristics (drug reimbursement) Inner setting: readiness for implementation: (iii) access to knowledge and information (education and training)	Serious
** Verbiest, 2013** [67]	Netherlands	Domain 8 59. Place innovation on fee for service lists/formularies AND Domain 9 69. Create or change credentialing and/or licensure standards	(i) Increasing access to health insurance coverage which included smoking cessation treatment (ii) Introduction of the first Dutch guideline ‘Treatment of Tobacco Dependence’			**(i) + ** **(ii) 0**		**(i) + ** **(ii) 0**	**Facilitators:** Inner setting: structural characteristics (increased access to cessation medications, health insurance coverage for smoking cessation treatment prompts GPs to prescribe evidence-based pharmaceuticals for smoking cessation)	Moderate
** Williams, 2004** [64]	Ireland	59. Place innovation on fee for service lists/formularies	Introduction of a new cessation medication (NRT) onto a country’s prescription scheme			** + / 0**			N/A	Serious
** Bailey, 2017** [50]	Oregon, USA	Domain 8 60. Alter incentive/allowance structures AND Domain 9 67. Change record systems	‘Meaningful use’ (MU) criteria (i) Change record systems: 2012: addition of 'readiness to quit' and 'counselling given' fields to the vital sign section of the medical record (ii) 2014: Full implementation of policy, including incentive payments	** + **	** + **	** + **		** + **	**Facilitators:** Inner setting: structural characteristics (inclusion of smoking status as a ‘vital sign’ increases the rate of identifying smokers)	Moderate
** Coleman, 2007** [32]	UK	60. Alter incentive/allowance structures	QOF 2004. Financially incentivised target for general practitioners: to record their patients’ smoking status (‘ever’); and to record smoking status every 15 months for patients who have coronary heart disease, diabetes mellitus, COPD, transient ischaemic attack or stroke, asthma, or hypertension, and every 15 months offer cessation advice or referral to a cessation service for these co-morbid patients who smoke	** + **	** + **	**0**			**Facilitators:** Inner setting: structural characteristics (availability of cessation services to refer patients to, availability of nicotine treatment to prescribe) **Barriers:** Implementation process: executing (no targets were set for prescribing nicotine addiction treatments; the rates of NRT prescriptions did not increase)	Serious
** Dhalwani, 2013** [38]	UK	60. Alter incentive/allowance structures	QOF 2004	** + **					**Facilitators:** Inner setting: readiness for implementation: (iii) access to knowledge and information (GPs' awareness of the impending introduction of the 2004 GP contract)	Serious
** Farley, 2017** [40]	UK	60. Alter incentive/allowance structures	QOF 2004	** + **	** + **	** + **		**0**	**Barriers:** Inner setting: culture (cancer patients would benefit if general practitioners became more actively involved in supporting smoking cessation) **Facilitators:** Outer setting: external policy and incentives (QOF incentive not targeting cancer patients resulted in the increase of smoking targets for cancer patients too)	Low
** Fichera, 2016** [45]	England	60. Alter incentive/allowance structures	QOF 2004		**0**	**0**			**Facilitators:** Inner setting: structural characteristics (improvements in care induced by the QOF for individuals with the targeted health conditions might include better monitoring of the condition, increased contacts with the doctor, healthcare, and lifestyle advice)	Moderate
** Fortmann, 2020** [56]	United States (multi-state)	Domain 8 60. Alter incentive/allowance structures AND Domain 9 71. Change accreditation or membership requirements	(i) Financial incentives via 'meaningful use' (MU) criteria (ii) Accreditation requirement change: "in 2011, the Health Resources and Services Administration (HRSA)… updated its standards for documenting smoking and cessation counselling; these standards apply to all community health centres (CHCs) certified as Federally Qualified Community Health Centres and meeting all reporting requirements is a condition of funding”	** + **					**Barriers:** Characteristics of individuals: other personal attributes (smoking status documentation was lower for younger patients, men, non-white subgroups, and patients with opioid use disorders) **Facilitators:** Characteristics of individuals: other personal attributes (most comorbidities were associated with higher odds of documented smoking status)	Moderate
** Hardy, 2014** [39]	UK	60. Alter incentive/allowance structures	QOF 2004		** + / 0**				**Facilitators:** Outer setting: external policy and incentives (QOF incentive not targeting pregnant patients resulted in the increase of smoking targets for these patients too)	Serious
** McGovern, 2008** [43]	Scotland	60. Alter incentive/allowance structures	QOF 2004	** + **	** + **				N/A	Serious
** Millett, 2007** [44]	UK	60. Alter incentive/allowance structures	QOF 2004	** + **	** + **				**Facilitators:** Outer setting: external policies and incentives (reduced tobacco use in society, financial incentives are likely to be most effective in reducing the prevalence of smoking when combined with other quality improvement initiatives [e.g. active dissemination of clinical guidelines on smoking cessation, ongoing training and support for front-line staff] within a comprehensive tobacco control strategy) Inner setting: readiness for implementation: (ii) available resources (ongoing training and support for front-line staff) Inner setting: readiness for implementation: (iii) access to knowledge and information (active dissemination of clinical guidelines on smoking cessation)	Serious
** Simpson, 2006** [49]	Scotland	60. Alter incentive/allowance structures	QOF 2004	** + **	** + **				**Barriers:** Characteristics of individuals: knowledge and beliefs about the intervention (patients in deprived areas and males may be less willing to seek advice for their condition) Characteristics of individuals: other personal attributes (average consultation length for deprived patients is ~ 1 to 2 min shorter than for affluent patients; this may have reduced the opportunity for GPs to record risk factors) **Facilitators:** Outer setting: external policies and incentives (other developments may have also contributed)	Serious
** Sutton, 2010** [47]	Scotland	60. Alter incentive/allowance structures	QOF 2004	** + **					N/A	Moderate
** Szatkowski, 2010** [29]	UK	60. Alter incentive/allowance structures	QOF 2004	** + **					N/A	Serious
** Szatkowski, 2011** [28]	England	60. Alter incentive/allowance structures	QOF 2004		** + / 0**				**Barriers:** Implementation process (discrepancy between practitioner-reported and patient-reported outcome measures is a problem)	Serious
** Szatkowski, 2016** [27]	England	60. Alter incentive/allowance structures	QOF 2012 amendment: encouraging GPs to offer referral to the NHS Stop Smoking Services and prescribe pharmacotherapy to all smokers, regardless of their smoking-related medical history		** + **	**0**			**Barriers:** Implementation process: executing (the electronic codes that GPs were able to use to receive payment included the 'record of cessation advice' code that they had used before the policy change, when the 2012 policy was not intending to incentivise this action)	Moderate
** Taggar, 2012** [30]	UK	60. Alter incentive/allowance structures	(i) QOF 2004 (ii) QOF 2006, QOF 2008 2006 amendment: recording smoking status in patients without smoking-related morbidity was required periodically (every 27 months) rather than ‘ever’ 2008 amendment: chronic kidney disease (CKD) and mental illness (schizophrenia, bipolar affective disorder and other psychoses) were added to the list of smoking-related conditions which required recording of smoking status and cessation advice every 15 months	**(i): + ** **(ii): 0**	**(i): + ** **(ii): 0**				**Facilitators:** Implementation process: executing (specific wording within QOF targets is influential on clinical behaviour)	Serious
** Tahrani, 2007** [42]	England	60. Alter incentive/allowance structures	QOF 2004	** + **	** + **				N/A	Serious
** Akman, 2017** [72]	Turkey	Domain 8 65. Use capitated payments AND Domain 9 66. Mandate change, 67. Change record systems, 71. Change accreditation or membership requirements	‘Health Transformation Program’ Capitated payments: “With the introduction of new structure, family doctors are paid on a capitation basis with incentives for selected preventive services” Mandate change:” To establish a stronger primary care system, in 2003 the Turkish government introduced the ‘Health Transformation Program’.” Change record systems: “Facilities for the family health centres were improved compared to former health centres including computerization enabling electronic record keeping.” Change accreditation or membership requirements: “Those primary care doctors who were formerly called ‘general practitioners’ were re-designated as ‘family doctors’ after completing a 10-day orientation course.”		**0**				**Facilitators:** Outer setting: external policy and incentives (other contributing factors, health agenda has shifted from communicable and vaccine preventable diseases to non-communicable diseases)	Serious
** Donner-Banzhoff, 1996** [75]	Germany vs UK	65. Use capitated payments	Fee-For-Service (FFS) based systems (Germany) vs Capitation (UK)		**0**	**0**			**Barriers:** Inner setting: culture (physicians show a lack of enthusiasm for encouraging smoking cessation because they are aware of the barriers that prevent their smoking patients from complying with their advice and the work does not conform with the traditional medical curative model)	Serious
**Domain 9. Change infrastructure**										
** Akman, 2017** [72]	Turkey	Domain 8 65. Use capitated payments AND Domain 9 66. Mandate change, 67. Change record systems, 71. Change accreditation or membership requirements	‘Health Transformation Program’ Capitated payments: “With the introduction of new structure, family doctors are paid on a capitation basis with incentives for selected preventive services” Mandate change:” To establish a stronger primary care system, in 2003 the Turkish government introduced the ‘Health Transformation Program’.” Change record systems: “Facilities for the family health centres were improved compared to former health centres including computerization enabling electronic record keeping.” Change accreditation or membership requirements: “Those primary care doctors who were formerly called ‘general practitioners’ were re-designated as ‘family doctors’ after completing a 10-day orientation course.”		**0**				**Facilitators:** Outer setting: external policy and incentives (other contributing factors, health agenda has shifted from communicable and vaccine preventable diseases to non-communicable diseases)	Serious
** Szatkowski, 2021** [36]	England	66. Mandate change	Change to the public health commissioning infrastructure			**-**			**Barriers:** Outer setting: external policies and incentives (where there is no local Stop Smoking Service to which general practitioners can refer pregnant women there is a reduced stimulus for discussion of smoking cessation and less direct prescribing of NRT)	Serious
** Bailey, 2017** [50]	Oregon, USA	Domain 8 60. Alter incentive/allowance structures AND Domain 9 67. Change record systems	‘Meaningful use’ (MU) criteria (i) Change record systems: 2012: addition of 'readiness to quit' and 'counselling given' fields to the vital sign section of the medical record (ii) 2014: Full implementation of policy, including incentive payments	** + **	** + **	** + **		** + **	**Facilitators:** Inner setting: structural characteristics (inclusion of smoking status as a ‘vital sign’ increases the rate of identifying smokers)	Moderate
** Dhalwani, 2014** [41]	UK	69. Create or change credentialing and/or licensure standards	Clinical guideline change; broadening of indications for NRT for pregnant women			**0**			**Facilitators:** Characteristics of individuals: other personal attributes (females with asthma or mental illnesses and those from more socioeconomically-deprived areas were more likely to receive prescriptions during pregnancy)	Serious
** Langley, 2011** [37]	England	Domain 8 59. Place innovation on fee for service lists/formularies AND Domain 9 69. Create or change credentialing and/or licensure standards	(i) Introduction of a new cessation medication (varenicline) onto a country’s prescription scheme, December 2006 (ii) Introduction of NICE guideline for varenicline, July 2007			**(i) + / 0** **(ii) + / 0**			**Facilitators:** Inner setting: readiness for implementation: (iii) access to knowledge and information (measures to increase physicians' confidence in the effectiveness and safety of the medication) Characteristics of individuals: knowledge and beliefs about the intervention (raising awareness of varenicline amongst smokers)	Moderate
** Langley, 2011** [34]	England	69. Create or change credentialing and/or licensure standards	Clinical guideline change; broadening of indications for NRT for adolescents			**0**			**Barriers:** Outer setting: patient needs and resources (teenagers make fewer visits to their GP than adults and may be less likely than adults to ask for NRT, therefore general practice may not be an effective setting for the distribution of NRT to people within this age group) Characteristics of individuals: knowledge and beliefs about the intervention (some young people would find using NRT embarrassing, unpleasant or expensive) Characteristics of individuals: knowledge and beliefs about the intervention (concerns among healthcare professionals as to the safety of NRT for teenagers) Inner setting: readiness for implementation: (iii) access to knowledge and information (lack of awareness of the licensing change among GPs)	Moderate
** Langley, 2012** [35]	England	69. Create or change credentialing and/or licensure standards	Clinical guideline change; broadening of indications for NRT for patients with cardiovascular disease			**0**			**Barriers:** Outer setting: external policies and incentives (factors other than the licensing change have led to a widespread decrease in prescribing for NRT)	Moderate
** Li, 2020** [55]	United States (multi-state)	69. Create or change credentialing and/or licensure standards	US Preventive Services Task Force (USPSTF) 2013 guideline recommendation to provide low-dose computed tomography for lung cancer screening (LDCT-LCS)	** + **	** + **	** + **			**Facilitators:** Outer setting: external policies and incentives (rereleased USPSTF recommendation in 2015 for clinicians to offer cessation support to smokers)	Serious
** Thorndike, 2007** [53]	United States (multi-state)	69. Create or change credentialing and/or licensure standards	Release and update of the US Public Health Service evidence-based national guidelines for the treatment of tobacco use	**0**	**0**				**Barriers:** Inner setting: structural characteristics (lack of time to provide adequate preventive counselling, lack of insurance coverage for smoking cessation pharmacotherapies) Characteristics of individuals: other personal attributes (competing demands of other medical problems during a visit) **Facilitators:** Outer setting: cosmopolitanism (embedding physicians in a broader system that integrates smoking cessation treatment more easily into practice [facilitating referral] and cessation support outside the office) Inner setting: implementation climate: (iii) relative priority (most physicians regard addressing smoking as important) Characteristics of individuals: knowledge and beliefs about the intervention (most physicians report feeling prepared to counsel about smoking)	Moderate
** Verbiest, 2013** [67]	Netherlands	Domain 8 59. Place innovation on fee for service lists/formularies AND Domain 9 69. Create or change credentialing and/or licensure standards	(i) Increasing access to health insurance coverage which included smoking cessation treatment (ii) Introduction of the first Dutch guideline ‘Treatment of Tobacco Dependence’			**(i) + ** **(ii) 0**		**(i) + ** **(ii) 0**	**Facilitators:** Inner setting: structural characteristics (increased access to cessation medications, health insurance coverage for smoking cessation treatment prompts GPs to prescribe evidence-based pharmaceuticals for smoking cessation)	Moderate
** Fortmann, 2020** [56]	United States (multi-state)	Domain 8 60. Alter incentive/allowance structures AND Domain 9 71. Change accreditation or membership requirements	(i) Financial incentives via 'meaningful use' (MU) criteria (ii) Accreditation requirement change: "in 2011, the Health Resources and Services Administration (HRSA)… updated its standards for documenting smoking and cessation counselling; these standards apply to all community health centres (CHCs) certified as Federally Qualified Community Health Centres and meeting all reporting requirements is a condition of funding”	** + **					**Barriers:** Characteristics of individuals: other personal attributes (smoking status documentation was lower for younger patients, men, non-white subgroups, and patients with opioid use disorders) **Facilitators:** Characteristics of individuals: other personal attributes (most comorbidities were associated with higher odds of documented smoking status)	Moderate
** Peterson, 2016** [52]	United States (multi-state)	71. Change accreditation or membership requirements	Accreditation program for primary care physicians		** + **				**Barriers:** Inner setting: implementation climate: (ii) compatibility (QI is difficult to sustain if it is not integrated into the existing culture and systems of care)	Serious
** Shi, 2017** [59]	United States (multi-state)	71. Change accreditation or membership requirements	Changing standards for primary care practices—'Patient-centered medical home' (PCMH) recognition status	** + **	** + **	** + **			N/A	Moderate
** Van Doorn-Klomberg, 2014** [68]	Netherlands	71. Change accreditation or membership requirements	Changing standards for primary care practices	** + / 0**	** + / 0**			**0**	**Facilitators:** Implementation process: reflecting and evaluating (audit and feedback as a central mechanism) Outer setting: external policies and incentives (other developments in the primary care field) Intervention characteristics: complexity (adaptations to the program were made to reduce the burden of work) Intervention characteristics: adaptability (health professionals can take ownership of the improvement plans that are tailored to the individual practices)	Moderate
**Multiple domains**^a^										
** Akman, 2017** [72]	Turkey	Domain 8 65. Use capitated payments AND Domain 9 66. Mandate change, 67. Change record systems, 71. Change accreditation or membership requirements	‘Health Transformation Program’ Capitated payments: “With the introduction of new structure, family doctors are paid on a capitation basis with incentives for selected preventive services” Mandate change:” To establish a stronger primary care system, in 2003 the Turkish government introduced the ‘Health Transformation Program’.” Change record systems: “Facilities for the family health centres were improved compared to former health centres including computerization enabling electronic record keeping.” Change accreditation or membership requirements: “Those primary care doctors who were formerly called ‘general practitioners’ were re-designated as ‘family doctors’ after completing a 10-day orientation course.”		**0**				**Facilitators:** Outer setting: external policy and incentives (other contributing factors, health agenda has shifted from communicable and vaccine preventable diseases to non-communicable diseases)	Serious
** Bailey, 2017** [50]	Oregon, USA	Domain 8 60. Alter incentive/allowance structures AND Domain 9 67. Change record systems	‘Meaningful use’ (MU) criteria (i) Change record systems: 2012: addition of 'readiness to quit' and 'counselling given' fields to the vital sign section of the medical record (ii) 2014: Full implementation of policy, including incentive payments	** + **	** + **	** + **		** + **	**Facilitators:** Inner setting: structural characteristics (inclusion of smoking status as a ‘vital sign’ increases the rate of identifying smokers)	Moderate
** Fortmann, 2020** [56]	United States (multi-state)	Domain 8 60. Alter incentive/allowance structures AND Domain 9 71. Change accreditation or membership requirements	(i) Financial incentives via 'meaningful use' (MU) criteria (ii) Accreditation requirement change: "in 2011, the Health Resources and Services Administration (HRSA)… updated its standards for documenting smoking and cessation counselling; these standards apply to all community health centres (CHCs) certified as Federally Qualified Community Health Centres and meeting all reporting requirements is a condition of funding”	** + **					**Barriers:** Characteristics of individuals: other personal attributes (smoking status documentation was lower for younger patients, men, non-white subgroups, and patients with opioid use disorders) **Facilitators:** Characteristics of individuals: other personal attributes (most comorbidities were associated with higher odds of documented smoking status)	Moderate
** Langley, 2011** [37]	England	Domain 8 59. Place innovation on fee for service lists/formularies AND Domain 9 69. Create or change credentialing and/or licensure standards	(i) Introduction of a new cessation medication (varenicline) onto a country’s prescription scheme, December 2006 (ii) Introduction of NICE guideline for varenicline, July 2007			**(i) + / 0** **(ii) + / 0**			**Facilitators:** Inner setting: readiness for implementation: (iii) access to knowledge and information (measures to increase physicians' confidence in the effectiveness and safety of the medication) Characteristics of individuals: knowledge and beliefs about the intervention (raising awareness of varenicline amongst smokers)	Moderate
** Mullins, 2009** [51]	Delaware, USA	Domain 5 40. Distribute educational materials 42. Conduct educational meetings AND Domain 7 54. Prepare patients/consumers to be active participants	‘Ask and Act program’ Program contains: (i) educational component for physicians (free patient materials for offices, continuing medical education programs for physicians and allied health professionals, and information on evidence-based interventions), and (ii) free patient materials which engage patients (patient materials include pre-printed prescription pads with tips on how to quit, brochures, and laminated quitline referral cards. Metal lapel pins and wall posters act as visual cues to encourage patients to ask their family physician for help, and a guide to tobacco cessation group visits details how practices can organize and bill for counselling sessions)		** + **			** + **	**Facilitators:** Inner setting: readiness for implementation: (iii) access to knowledge and information (physicians reported that they felt more comfortable with smoking cessation counselling and billing for this intervention, and that they were more likely to counsel their patients after hearing the presentation)	Serious
** Verbiest, 2013** [67]	Netherlands	Domain 8 59. Place innovation on fee for service lists/formularies AND Domain 9 69. Create or change credentialing and/or licensure standards	(i) Increasing access to health insurance coverage which included smoking cessation treatment (ii) Introduction of the first Dutch guideline ‘Treatment of Tobacco Dependence’			**(i) + ** **(ii) 0**		**(i) + ** **(ii) 0**	**Facilitators:** Inner setting: structural characteristics (increased access to cessation medications, health insurance coverage for smoking cessation treatment prompts GPs to prescribe evidence-based pharmaceuticals for smoking cessation)	Moderate

A summary of the key results. The included studies are ordered by implementation strategy domain (5, 7, 8 and 9 and ‘Multiple domains’). Within the domains, the studies are ordered by implementation strategy category then alphabetically by first author surname

(Wright, 2018) [71] was excluded from narrative synthesis as it was at critical risk, so it is excluded from this table

^a^Note: the studies under ‘Multiple domains’ are also listed above in the relevant separate domains

Effectiveness outcomes key:

• + : positive effect on outcome

• -: negative effect on outcome

• 0: no significant effect on outcome

•: outcome was not assessed

### RQ2: Effectiveness & RQ3: Perceived Facilitators and Barriers

For conciseness and clarity, we present the effectiveness findings and the key facilitators and barriers proposed by the included studies’ authors together in this section, organised by implementation strategy domain and category. Details can be found in Table 2 and a summary of the facilitators and barriers in Table 3. The extracted quantitative outcomes are included in Appendix 8.

**Table 3:** Summary of perceived facilitators and barriers

**Construct**	**Perceived facilitators and barriers**
Intervention characteristics	Facilitators: Intervention should be simple, accessible, and adaptable.
Outer setting	Facilitators: policies and incentives (tobacco control measures, anti-smoking social norms, funding for public health and cessation clinics), systems-level interventions allowing easy referral to cessation programs or community-based support.
Inner setting	Barriers: time and resource constraints, lack of access to free cessation medications and follow-up appointments, incompatibility with and lower priority of smoking cessation compared to existing clinical demands, lack of knowledge and training around guideline changes.
Patient/physician characteristics	Barriers: smokers with greatest risk of cardiovascular disease are less likely to take up health check invitations, some patient characteristics are associated with worse smoking outcome measures, smokers’ lack of awareness of, and negative perceptions about, the effectiveness of cessation medications; physicians having doubts about the effectiveness and safety of cessation interventions and not feeling competent to deliver cessation counselling.
Implementation process	Facilitators: multifaceted approaches to intervention implementation (which include audit and feedback) Barriers during the execution of intervention implementation delivery: wording/coding of targets not optimally targeting the desired clinical behaviours/outcome measures, focussing on the ‘risk factor identification’ and not the ‘intervention’ aspects of cessation treatment, lack of sustained advertising of cessation support, insufficient messaging to patients trying to quit smoking about the cessation support options that are available

This table shows a condensed summary of the key facilitators and barriers from the included studies. Perceived facilitators and barriers, extracted from the studies, were mapped to the constructs defined by the Consolidated Framework for Implementation Research (CFIR) [26] (Appendix 7)

#### Utilize financial strategies (Domain 8)

Thirty-four studies [27–32, 37–40, 42–45, 47–50, 54, 56–58, 60–67, 69, 72, 73, 75] evaluated interventions using an implementation strategy that increased funding towards the provision of smoking cessation treatment in primary care.

##### Fund and contract for the clinical innovation (Category 57)

The six studies in this category investigated policies where primary care practices received funding to deliver national cardiovascular disease prevention programs (including health checks). Two studies were at low risk of bias [31, 48], two moderate [65, 73], and two serious [66, 69].

Effectiveness. For practitioner-level outcomes: two studies showed an increase in smoking status recording [48, 73]; two indicated an increase [31, 69] and one no effect [73] on the provision of cessation advice; and one increased [31] and one had no effect [73] on cessation medication prescribing. For patient-level outcomes, three studies indicated an increase [31, 65, 66] in cessation while one showed no effect [73].

Facilitators/barriers. A perceived barrier was that health check programs focused on the ‘risk factor identification’ and not the ‘intervention’ aspects of cessation treatment [31, 73]. Another barrier was time constraints and insufficient financial recompense for physicians to deliver cessation treatment [31, 66, 69, 73]. Authors noted that there was selection bias in the type of patients who respond to an invitation for a health check [31, 48], but that the value of opportunistic health checks should not be underplayed [31]. A proposed facilitator to increase effectiveness was improved linkages to community-based programmes and support [65, 69] or improved mechanisms for follow-up/monitoring of cardiovascular risk factors in primary care [31, 66].

##### Place innovation on fee for service lists/formularies (Category 59)

The 10 studies in this category examined changes in insurance schemes which included aspects of smoking cessation treatment [54, 57, 58, 60–62, 67] or the introduction of a new smoking cessation medication [37, 63, 64]. One study was at low risk of bias [62], six moderate [37, 54, 57, 58, 60, 67], and three serious [61, 63, 64].

Effectiveness. For practitioner-level outcomes, the introduction of a new cessation medication onto a country’s prescription scheme – NRT in Ireland in 2001 [63, 64], and varenicline in England in 2006 [37] – increased the prescription of the new medication, but did not change overall prescribing of smoking cessation medications. For practitioner-level outcomes, in the USA, increasing access to health insurance coverage which included smoking cessation treatment, increased smoking status recording (multi-state, Oregon) [61, 62], cessation advice provision (Colorado) [58] and cessation medication prescribing (Oregon, multi-state) [54, 60]. In the Netherlands [67], increasing health insurance coverage for smoking cessation also increased cessation medication prescribing. For patient-level outcomes, in the USA, one study (Massachusetts) found no difference in quit attempts [57] but two studies (Oregon, and multi-state) found a positive effect on smoking cessation following the increases in medication prescribing [54, 60]. The Dutch study [67] indicated increased cessation, but evidence for this was less robust. Patient-level outcomes were not measured in the studies assessing the introduction of new medications.

Facilitators/barriers. Perceived barriers were that physician confidence in, and patients’ awareness of, cessation medications was too low [37]. A proposed facilitator of increasing access to health insurance coverage was that this increases access to medications and primary care services [54, 60], which in turn increase the odds that services like smoking status assessment would be performed [62]. Other proposed facilitators included structural characteristics, such as providing sufficient education/training about the 5As/VBA [63], delivering 5As/VBA as an organisational priority and allocating sufficient physician time for it [57, 58, 63].

##### Alter incentive/allowance structures (Category 60)

Of the 16 studies in this category, two studies in the USA (Oregon, and multi-state) [50, 56] investigated the ‘Meaningful use’ (MU) scheme, which included the introduction of incentive payment for physicians to record their patients’ smoking status and offer cessation assistance alongside other measures (such as changing recording systems) from 2011. The other 14 studies examined various amendments of the Quality and Outcomes Framework (QOF), a pay-for-performance scheme in the UK which financially incentivised GPs to perform certain interventions.

- 13 studies [28–30, 32, 38–40, 42–45, 47, 49] investigated the 2004 QOF, which set the following targets: every 15 months record smoking status for patients who have coronary heart disease, diabetes mellitus, COPD, transient ischaemic attack or stroke, asthma, or hypertension; and every 15 months offer cessation advice or referral to a cessation service for these co-morbid patients who smoke.
- One study [30] investigated the 2004, 2006 and 2008 QOF. 2006 amendment: record smoking status in patients without smoking-related morbidity every 27 months rather than ‘ever’. 2008 amendment: chronic kidney disease, schizophrenia, bipolar disorder, and other psychoses were added to the list of smoking-related conditions which required recording of smoking status and cessation advice every 15 months.
- One study [27] investigated the 2012 QOF amendment: offer referral to the National Health Service Stop Smoking Services (NHS SSS) and prescribe pharmacotherapy to all people who smoke, regardless of their smoking-related medical history.

In this category, one study [40] was at low risk of bias, five moderate [27, 45, 47, 50, 56], and ten serious [28–30, 32, 38, 39, 42–44, 49]; most of the latter did not account for underlying secular trends.

Effectiveness. For practitioner-level outcomes, several studies in the UK for the 2004 QOF found increased smoking status [29, 30, 32, 38, 40, 42–44, 47, 49] and cessation advice recording [30, 32, 40, 42–44, 49] in primary care for all patients and those who had a QOF-targeted-morbidity. However, one study of survey participants with a QOF-targeted-morbidity found that the recall of receiving cessation advice by patients did not increase significantly [45]; one study found that there was an increase in cessation advice provision to pregnant women who smoked [not direct targets of this policy] but this increase was not sustained long term [39]; and one study which compared the rate of cessation advice recording in primary care electronic health records with the rate of patient recall of receiving cessation advice found mixed results (increase in the former, no effect on the latter) [28]. Two studies found that there was no effect of the 2004 QOF on cessation medication prescribing [an indirect target of the policy] [32, 45], while one found an increase in cessation medication prescribing [40]. The study which assessed the 2006 and 2008 revisions of the QOF only examined practitioner-level outcomes and found no significant effect on smoking status recording or cessation advice provision (but the outcome for the 2008 QOF is less robust) [30]. The one study investigating the 2012 QOF amendment also only examined practitioner-level outcomes and found an increase in the provision of cessation advice and referrals to NHS SSS, but no increase in cessation medication prescribing [27]. In the USA, the two studies (Oregon, and multi-state) [50, 56] examined the introduction of incentive payments via the ‘Meaningful Use’ scheme, however as this intervention included several measures from multiple implementation strategy domains, it is not possible to disentangle individual effects. These studies found an increase for practitioner-level outcomes: an increase in smoking status recording [50, 56], cessation counselling [50] and cessation medication prescribing [50]. For patient-level outcomes, one study indicated an increase in cessation too [50]. In contrast, the only study assessing a patient-level outcome of the QOF 2004 found no effect on cessation [40].

Facilitators/barriers. A suggested barrier to effectiveness on the cessation medication prescribing outcome was incorrect wording/electronic coding of clinical targets [27, 30, 32] – authors recommended that the clinical behaviours and outcome measures targeted are made clearer [27]. Another proposed barrier to effectiveness was the way the implementation outcomes are measured: some authors suggested that any observed increase in cessation advice-giving may not reflect an increase in ‘real life’, but rather more complete recording of advice GPs were already giving [28, 32]. Alternatively, GPs may have increased their provision of cessation advice, but patients were not recognising it as ‘advice’ [28, 39], perhaps due to improper practitioner training on smoking cessation and the delivery of the 5As/VBA [39]. Hence, uncertainty regarding real-world cessation advice provision may be the reason for the mixed effect observed for the cessation medication prescribing outcome. A proposed facilitator was to combine financial incentives with other quality improvement initiatives, such as active dissemination of cessation guidelines and ongoing training [39] and support for front-line staff, within a comprehensive tobacco control strategy [44].

##### Use capitated payments (Category 65)

The two studies [72, 75] in this category assessed capitated payments (where providers of care are given a set amount of money per patient for delivering clinical care). One [72] (described in more detail in Category 66 below) assessed other measures in addition to capitated payments so it is not possible to disentangle individual effects there. Both studies in this category were at serious risk of bias.

Effectiveness. The two studies [72, 75] found no effect on cessation advice provision, and one [75] found no effect on cessation medication prescribing. No patient-level outcomes were measured.

Facilitators/barriers. A proposed barrier was regarding cultural factors within health infrastructures, one study suggested that physicians did not consider cessation treatment to fit with the “traditional curative model of medicine” and that physicians assume they know the barriers which prevent their patients from quitting [75].

#### Change infrastructure (Domain 9)

Fourteen studies [34–37, 41, 50, 52, 53, 55, 56, 59, 67, 68, 72] evaluated an intervention using an implementation strategy that involved infrastructure change aiming to increase the provision of smoking cessation treatment in primary care.

##### Mandate change (Category 66)

The two studies [36, 72] assessed aspects of wider infrastructure change which aimed to increase smoking cessation treatment provision in primary care. One study [72] investigated a broad national health infrastructure change occurring between 2003–2010 in Turkey (‘Health Transformation Program’) alongside other measures, the other study [36] investigated a change in 2013 to the public health commissioning infrastructure in England (where responsibility for commissioning cessation services was transferred to regional budgets). Both studies were at serious risk of bias.

Effectiveness. Only practitioner-level outcomes were measured. The Turkish study [72] found no effect on the provision of cessation counselling. The English study [36] found a negative effect on the prescribing of any and dual NRT to pregnant women who smoke.

Facilitators/barriers. A proposed barrier for effectiveness was that external policies which indirectly result in the decommissioning of cessation services decrease the stimulus for GPs to discuss smoking cessation and directly prescribe NRT in primary care [36].

##### Change record systems (Category 67)

The two studies in this category both assessed multiple implementation strategy domains, so it is not possible to disentangle individual effects for this category. Both studies have been described earlier: one examined the ‘Meaningful use’ (MU) scheme in Oregon, USA which included changing recording systems alongside other measures [50], and the other was the broad health infrastructure change in Turkey which included changing recording systems as one of its measures [72]. One study was at serious risk of bias [72] and one moderate [50].

Effectiveness. For practitioner-level outcomes, the Turkish study found no effect on the provision of cessation counselling [72]. The Oregon study [50] found increased smoking status recording, cessation counselling and prescribing of cessation medications. Patient-level outcomes were only assessed in the Oregon study which also indicated an increase in cessation [50].

Facilitators/barriers. A proposed facilitator from the Oregon study which indicated effectiveness was that the change to the recording system aligned well with an existing practice in the clinic (having smoking status as a ‘vital sign’) [50].

##### Create or change credentialing and/or licensure standards (Category 69)

The seven studies in this category investigated either an expansion to the indications for NRT to new patient populations in primary care [34, 35, 41] or the publication of new/updated national guidelines regarding smoking cessation treatment [37, 53, 55, 67]. Five studies were at moderate risk of bias [34, 35, 37, 53, 67], two serious [41, 55].

Effectiveness. Most of the effects were measured for practitioner-level outcomes. In the UK, the expansion of indications for NRT in 2005 did not increase prescribing of NRT to pregnant women who smoke [41], to adolescents who smoke [34], or to patients who have cardiovascular disease who smoke [35]. Publication of the national guideline related to varenicline in 2007 in the UK increased prescribing of varenicline but had no effect on the overall prescribing rate for cessation medications [37]. In the USA (multi-state), the release (1996) and update (2000) of the national guidelines for the treatment of tobacco use had no impact on the recording of smoking status or cessation advice [53]. In the USA (multi-state), the 2013 national guideline recommendation to provide low-dose computed tomography for lung cancer screening for certain patients who smoke led to an increase in cessation counselling recording and referral to smoking cessation programs, and increased smoking cessation medication prescribing [55] – however the outcome measure for this study may have been confounded by the re-released 2015 national guideline recommendation for clinicians to offer cessation support to smokers. In the Netherlands, the introduction of the first national tobacco treatment guideline in 2007 did not have any significant immediate or long-term trend impact on primary care prescriptions of smoking cessation medications or dispensed prescriptions [67]. The only study to assess a patient-level outcome was the Dutch study, which found no significant effect of this intervention on cessation [67].

Facilitators/barriers. A proposed barrier for the lack of increase on NRT prescribing to patients who have cardiovascular disease who smoke was that external factors – perhaps even the increase in the prescription of varenicline – led to a widespread decrease in prescribing for NRT [35]. Regarding guideline publications, a proposed facilitator to achieve effectiveness was that future guideline changes should be accompanied by other measures which target the time barriers that clinicians face, such as systems-level interventions that can identify patients’ smoking status and support clinicians’ efforts by facilitating referral to resources outside the physician’s office [53].

##### Change accreditation or membership requirements (Category 71)

Of the five studies in this category, two studies [52, 72] investigated accreditation programs for primary care *physicians* and were at serious risk of bias. The other three [56, 59, 68] investigated changing accreditation standards for primary care *practices* and were at moderate risk of bias.

Effectiveness. For the accreditation programs for physicians [52, 72], only practitioner-level outcomes were measured. The program in Turkey (already described) found no effect on the provision of smoking cessation counselling [72]. The other study in the USA (multi-state) [52] found that patient-recalled cessation advice increased significantly post-intervention. For the accreditation standards for practices [56, 59, 68], most of the effects were measured for practitioner-level outcomes. In the two multi-state studies in the USA, there was an increase in the recording of smoking status [56, 59] and provision of cessation interventions [59] following the change of standards which applied to community health centres. However, in one of the studies [56] (described above), incentive payments were also introduced as the change to standards occurred so the effects of individual implementation strategies cannot be disentangled. In the Netherlands [68], the accreditation program for primary care in 2005 had a mixed effect on smoking status recording (no effect for COPD patients, but positive effect for cardiovascular patients) and an uncertain effect on cessation advice provision. This study had no effect on the patient-level outcome: cessation [68].

Facilitators/barriers. A proposed facilitator was that quality improvement interventions may be effective if they are compatible with and integrated into the clinics’ usual culture and systems of care [52]. These may be attractive to physicians because they can take ownership of the tailored improvement plans, but the intervention should be simple [68].

#### Engage consumers (Domain 7)

Three studies [33, 46, 51] evaluated an intervention which used an implementation strategy that involved engaging people who smoke to raise awareness about the availability of cessation treatment in primary care. The facilitators and barriers are discussed for the domain.

##### Prepare patients/consumers to be active participants (Category 54)

Of the two studies in this category, one examined the introduction of smoke-free legislation in England [33] because an indirect target was to increase smoking cessation treatment in primary care. The other study in Delaware, USA was the ‘Ask and Act’ program which displayed patient materials in primary care clinics designed to engage patients who smoked, alongside other measures [51]. One study was at moderate risk of bias [33] and one serious [51].

Effectiveness. One study found that prescribing of all smoking cessation medications increased in the months leading up to the introduction of smoke-free legislation, but this increase was not sustained [33]. The other study found an increase in cessation advice recording (practitioner-level outcome) and an increase in cessation (patient-level outcome) following the program which engaged patients who smoked [51], but the authors implied the effect was more likely due to the other ‘educating healthcare professionals’ components of the intervention (Domain 5, below).

##### Use mass media (Category 56)

The only study [46] in this category evaluated the impact of anti-tobacco mass media advertising and pharmaceutical company-funded smoking cessation medication advertising in England, and was at moderate risk of bias.

Effectiveness. The only relevant finding was that neither intervention had a significant effect on NRT prescribing in primary care (practitioner-level outcome) [46].

Facilitators/barriers: A proposed facilitator was that when engaging consumers, the intervention needs to be sustained for longer durations [33, 46] or that consumers need to be engaged in multiple ways, considering other contextual factors and social norms around tobacco use [33].

#### Train and educate stakeholders (Domain 5)

Three studies in this domain [51, 70, 74] evaluated an intervention which used an implementation strategy that involved training and educating healthcare professionals in primary care who deliver cessation treatment. The facilitators and barriers are discussed for the domain.

##### Distribute educational materials (Category 40)

Of the two studies in this category, one examined the ‘Ask and Act’ program in Delaware, USA (mentioned above) which included a measure where cessation materials were distributed to physicians [51]. The other study evaluated an intervention where an educational pack designed to prompt the delivery of smoking status assessment and cessation advice was distributed to GPs in Victoria, Australia [70]. Both studies [51, 70] were at serious risk of bias.

Effectiveness. For practitioner-level outcomes, one study [70] found no effect on smoking status recording but both studies found an increase in cessation advice provision [51, 70]. For patient-level outcomes, one study [51] indicated increased cessation – however, the intervention also involved ‘Conduct educational meetings’ (Category 42) and ‘Prepare patients/consumers to be active participants’ (Domain 7, Category 54), but the authors implied that the effect can likely be attributed to Domain 5.

##### Conduct educational meetings (Category 42)

Of the two studies in this category, one examined the ‘Ask and Act’ program in Delaware, USA (mentioned above) which included a measure which delivered continuing medical education programs for physicians [51]. The other study evaluated the Finnish ‘National Programme for Chronic Bronchitis and COPD 1998–2007’ where training events were organised for primary healthcare personnel [74]. Both studies [51, 74] were at serious risk of bias.

Effectiveness. For practitioner-level outcomes, one study [74] found a positive effect on smoking status recording and the other study [51] found increased cessation advice recording. The Delaware study [51] also found an increase in cessation (patient-level outcome), but, as aforementioned, this intervention covered multiple implementation strategy categories.

Facilitators/barriers: For this domain, proposed facilitators were the simplicity of the educational material the physicians received [70] and it was suggested that educating physicians in smoking cessation treatment can lead to physicians feeling more comfortable with delivering and billing for cessation counselling [51].

### RQ4: Cost-effectiveness of implementation strategies

Some studies [28, 30, 32, 38, 43, 45, 47, 66, 69] included the cost of the interventions but none investigated cost-effectiveness.

## Discussion

### Summary of evidence

This systematic review aimed to find evidence for the adoption of implementation strategies on a national/state-wide scale, and effectiveness and cost-effectiveness regarding smoking cessation treatment provision and patient smoking outcomes in real-world primary care settings. The 49 included studies assessed only four out of nine implementation strategy domains. The majority of studies identified in this review did not measure patient-level outcomes. We found some evidence for interventions which utilized financial strategies having a beneficial impact on cessation. There were 34 studies which investigated interventions utilizing financial strategies, with only four being at low risk of bias. These appeared to increase the recording of smoking status and cessation advice, but the effect on cessation medication prescribing was mixed. Only one study assessed quit attempts and it found no effect, but seven out of nine studies which assessed smoking cessation found an increase. There were 14 studies which investigated interventions changing infrastructure, none at low risk of bias. These had mixed results for smoking status recording, cessation advice provision and cessation medication prescribing. No studies measured quit attempts, and one out of three studies which assessed smoking cessation found an increase. Only three studies, all at serious risk of bias, investigated interventions which trained and educated stakeholders. These indicated a beneficial impact on smoking status and cessation advice recording, and smoking cessation, but should be interpreted with caution because the evidence was low-quality. There were three studies which investigated interventions engaging consumers, none at low risk of bias. Two studies showed no effect on cessation medication prescribing in primary care. One study assessed cessation advice provision and cessation (both increased), but the intervention also involved implementation strategy categories which involved training and educating stakeholders and the effectiveness was attributed to this latter domain by the study authors. No studies assessed cost-effectiveness.

Authors of the included studies suggested a range of barriers and facilitators. Some key facilitators were the simplicity of the intervention and external policies/incentives which were complementary to the smoking cessation aims of the intervention (such as, wider tobacco control measures and funding for public health and cessation clinics) and having the ability for physicians to refer smokers to cessation programs or community-based support. Some of the key barriers included time and financial constraints, lack of free cessation medications and follow-up, deprioritisation and unclear targets in primary care, lack of knowledge of healthcare professionals, and insufficient messaging to patients about available cessation support options. Some of the key barriers identified were similar to those identified recently by the UK Royal College of Physicians [76].

This review complements the findings of a recent Cochrane review [20] which evaluated randomised and cluster-randomised trials of similar interventions but in controlled environments. There appears to be a ‘gap’ between the implementation strategies that have been enacted on a national/state-wide scale (identified by this review) and those demonstrating efficacy in trials [20]. While trials indicated efficacy of adjunctive counselling and tailored print materials on quit rate [20], no studies have assessed these interventions in national implementation.

Trials found a beneficial impact of adding cost-free medications to standard cessation support on smoking quit rates and quit attempts [20]. Regarding real-world implementation, this review found some evidence that increasing access to health insurance which included coverage for smoking cessation treatment had a beneficial impact on the recording of smoking status, the provision of cessation advice and cessation medications, and cessation. The only study which assessed quit attempts found no effect [57]. Where new free cessation medications were introduced, prescribing of the new medication increased but there was no change in overall prescribing for cessation medications (other outcomes were not assessed).

Trials found no clear evidence that provider incentives could increase smoking cessation [20]. In real-world implementation of financial incentives studied in this review, cessation outcomes were only assessed in two out of 16 studies (one showed an increase [50], one no effect [40]). We found evidence that a nationally implemented financial incentive for GPs was effective in increasing the recording of smoking status and cessation advice, and (in one study [27]) referral to cessation services; however, there was a mixed effect on cessation medication prescribing and smoking cessation. We also identified studies where primary care practices received funding to deliver national cardiovascular disease prevention programs (including health checks); these overall indicated increased smoking status recording, cessation advice and cessation medication provision, and cessation. There was no robust evidence regarding capitated payments.

Trials found some evidence for provider training, either individually or in combination with other interventions: the former having some beneficial impact on smoking status recording, cessation advice provision, cessation counselling, and providing self-help materials; the latter, a beneficial impact on quit rates and some outcomes of cessation assistance (setting a quit date, providing self-help materials, and arranging patient follow-up) [20]. We identified some low-quality evidence of provider training as a ‘real-world’ intervention (three studies, all at serious risk of bias), having a beneficial impact on smoking status recording, cessation advice recording, and cessation.

### Strengths and limitations

A robust approach was used to identify and synthesise relevant literature using a pre-registered protocol and a comprehensive search strategy. However, the search strategy may not have identified all relevant papers, because different terminology exists internationally for ‘primary care setting’ and no effective observational study filter exists [77]. To mitigate against this, the search terms from a recent Cochrane review [20] were used, the search strategy was piloted, and backward and forward citation tracking of included studies was conducted. A limitation is that only articles in English were included.

This systematic review investigated the scalability of national and state-wide policies, where policies were implemented without researcher input over large geographical areas, potentially diverse in patient and provider characteristics. This review evaluated observational studies which, whilst at risk of bias and unable to demonstrate causality, can provide evidence of real-world implementation. A large number of studies were included in the evidence synthesis, however, only half were at moderate or low risk of bias. Despite an international scope, most studies were set in the UK and the USA. In six studies, the intervention involved multiple implementation strategy categories and it was challenging to disentangle their individual effects.

### Implications and recommendations

Our findings indicate that during the development of future implementation strategies, a significant consideration should be given to the current demands of the primary care setting, such as existing time constraints and clinical priorities; future implementation strategies should better align with existing technologies and the routine systems in place; and the clinical outcomes which are targeted should be clearly communicated. We recommend profiling, both in the clinic and in government papers, that smoking cessation is a key priority and that various cessation support is available.

Future research could investigate the five implementation strategy domains not identified by this review (‘Use of evaluative and iterative strategies’, ‘Provide interactive assistance’, ‘Adapt and tailor to context’, ‘Develop stakeholder inter-relationships’, ‘Support clinicians’) and the strategies that were efficacious in the controlled-trial setting [20]: adjunctive counselling and tailored print materials. However, we recommend that the perceived facilitators and barriers identified by this review are considered when designing interventions.

We advise that hybrid effectiveness-implementation designs [15] are used, where studies robustly assess both the effectiveness of implementation strategies on (practitioner-level) provider performance as well as (patient-level) smoking outcomes. Additionally, we recommend measuring ‘advice provision about e-cigarettes’ as an additional outcome – due to the relative novelty of e-cigarettes being recommended as harm reduction tools in clinical guidelines (in 2021 in the UK [6] and Australia [78]), none of the studies in this review investigated this. Lastly, we recommend using methods such as Multiphase Optimization Strategy (MOST) [79], which consider the time and resource constraints of clinical settings, and verify that all the components of the 5As/VBA or the proposed implementation strategy interventions are optimised and cost-effective.

## Conclusions

This systematic review aimed to find evidence for the adoption, on a national or state-wide scale, of implementation strategies aiming to increase smoking cessation treatment provision in real-world primary care settings. The implementation strategies identified involved utilizing financial strategies, changing infrastructure, training and educating stakeholders, and engaging consumers. The first three strategies appeared to increase the rate of smoking status recording and cessation advice provision in primary care. The most amount of evidence was identified for the utilizing financial strategies domain, which also appeared to increase smoking cessation.

## Supplementary Information

**Additional file 1: Appendix 1. **5As/Very Brief Advice table.

**Additional file 2: Appendix 2.  **Expert Recommendations for Implementing Change (ERIC) programme definitions of implementation strategies from Powell et al. and Waltz et al.

**Additional file 3: Appendix 3.  **Completed PRISMA_2020 checklist for reporting.

**Additional file 4: Appendix 4.  **Search terms and search strategy.

**Additional file 5: Appendix 5.  **Pre-piloted data extraction form fields.

**Additional file 6: Appendix 6.  **Risk of bias assessments.

**Additional file 7: Appendix 7.  **Consolidated Framework for Implementation Research (CFIR) determinants from Damschroder et al.

**Additional file 8: Appendix 8.  **Supplementary table containing long-form quantitative outcome measures for RQ2 effectiveness.

## Data Availability

The systematic review protocol (ID: CRD42021246683) is available at https://www.crd.york.ac.uk/prospero/display_record.php?RecordID=246683. As not all the included studies are available Open Access, the completed data extraction form and PDFs of the 49 included studies are available from the corresponding author on reasonable request.

## Abbreviations

CFIR: Consolidated Framework for Implementation Research
COPD: Chronic Obstructive Pulmonary Disease
ERIC: Expert Recommendations for Implementing Change
GP: General Practitioner
MOST: Multiphase Optimization Strategy
NHS: National Health Service
NRT: Nicotine Replacement Therapy
QOF: Quality and Outcomes Framework
ROBINS-I: Risk Of Bias In Non-Randomized Studies of Interventions
SSS: Stop Smoking Service
UK: United Kingdom
USA: United States of America
VBA: Very Brief Advice
