# Supplementary material for: Implementation strategies to increase smoking cessation treatment provision in primary care: a systematic review of observational studies
Source: BMC Prim Care. 2023 Jan 25;24:32. doi: 10.1186/s12875-023-01981-2 (PMC9875430; doi:10.1186/s12875-023-01981-2)
Supplement: Supplementary file 1 — Additional file 1: Appendix 1. 5As/Very Brief Advice table. [file 12875_2023_1981_MOESM1_ESM.docx]

**Appendix. 5As and 3As/Very Brief Advice**

A table outlining and comparing the steps involved in the 5As and 3As/Very Brief Advice process for delivering ‘brief advice’ about smoking cessation to service users.

| **5As** | **Very Brief Advice (VBA)** |
| --- | --- |
| Ask about tobacco use | Ask about current/past smoking behaviour |
| Advise to quit | Advise about the consequences of smoking and smoking cessation |
| Assess willingness to make a quit attempt |  |
| Assist in quit attempt (provide general assistance, prescribe cessation medications, set quit date, provide counselling, provide self‐help materials) | Act: Provide options for later/additional support, and advise on stop smoking medications. |
| Arrange follow-up appointment to address smoking |  |
