# Supplementary material for: Implementation strategies to increase smoking cessation treatment provision in primary care: a systematic review of observational studies
Source: BMC Prim Care. 2023 Jan 25;24:32. doi: 10.1186/s12875-023-01981-2 (PMC9875430; doi:10.1186/s12875-023-01981-2)
Supplement: Supplementary file 4 — Additional file 4: Appendix 4. Search terms and search strategy. [file 12875_2023_1981_MOESM4_ESM.docx]

**Appendix: Systematic review search terms and the search strategy**

Records identified from*:

Databases total (n = 12532)

Embase (n = 4964)

Medline (n = 2863)

APA PsycInfo (n = 955)

CINAHL (n = 1861)

Global Health (n = 652)

Social Policy and Practice (n = 28)

Applied Social Sciences Index and Abstracts (ASSIA) (n = 830)

OpenGrey (n = 11)

Social Care Online (n = 15)

Healthcare Management Information Consortium (HMIC) Database (n = 353)

Search and export: 7 April 2021

‘Citation, abstract, subject headings’ in RIS.

Embase 7 April 2021

Via Ovid

Database: Embase <1974 to 2021 Week 13>

Search Strategy:

--------------------------------------------------------------------------------

1 exp smoking/ (405134)

2 exp cigarette smoking/ (57862)

3 exp tobacco consumption/ (3011)

4 exp tobacco/ (47678)

5 exp tobacco dependence/ (21693)

6 tobacco.mp. [mp=title, abstract, heading word, drug trade name, original title, device manufacturer, drug manufacturer, device trade name, keyword, floating subheading word, candidate term word] (161193)

7 smoking.mp. [mp=title, abstract, heading word, drug trade name, original title, device manufacturer, drug manufacturer, device trade name, keyword, floating subheading word, candidate term word] (506719)

8 cigarett*.mp. [mp=title, abstract, heading word, drug trade name, original title, device manufacturer, drug manufacturer, device trade name, keyword, floating subheading word, candidate term word] (126082)

9 exp smoking cessation/ (61774)

10 smoking cessation.mp. [mp=title, abstract, heading word, drug trade name, original title, device manufacturer, drug manufacturer, device trade name, keyword, floating subheading word, candidate term word] (68962)

11 exp smoking cessation program/ (3482)

12 quit* smoking.mp. [mp=title, abstract, heading word, drug trade name, original title, device manufacturer, drug manufacturer, device trade name, keyword, floating subheading word, candidate term word] (11181)

13 stop* smoking.mp. [mp=title, abstract, heading word, drug trade name, original title, device manufacturer, drug manufacturer, device trade name, keyword, floating subheading word, candidate term word] (6500)

14 tobacco cessation.mp. [mp=title, abstract, heading word, drug trade name, original title, device manufacturer, drug manufacturer, device trade name, keyword, floating subheading word, candidate term word] (3388)

15 smoking abstinence.mp. [mp=title, abstract, heading word, drug trade name, original title, device manufacturer, drug manufacturer, device trade name, keyword, floating subheading word, candidate term word] (1997)

16 quit attempt*.mp. [mp=title, abstract, heading word, drug trade name, original title, device manufacturer, drug manufacturer, device trade name, keyword, floating subheading word, candidate term word] (3178)

17 quit date*.mp. [mp=title, abstract, heading word, drug trade name, original title, device manufacturer, drug manufacturer, device trade name, keyword, floating subheading word, candidate term word] (748)

18 exp primary medical care/ (111688)

19 primary care.mp. [mp=title, abstract, heading word, drug trade name, original title, device manufacturer, drug manufacturer, device trade name, keyword, floating subheading word, candidate term word] (169863)

20 primary medic*.mp. [mp=title, abstract, heading word, drug trade name, original title, device manufacturer, drug manufacturer, device trade name, keyword, floating subheading word, candidate term word] (114080)

21 primary health*.mp. [mp=title, abstract, heading word, drug trade name, original title, device manufacturer, drug manufacturer, device trade name, keyword, floating subheading word, candidate term word] (89046)

22 exp general practice/ (79786)

23 general practi*.mp. [mp=title, abstract, heading word, drug trade name, original title, device manufacturer, drug manufacturer, device trade name, keyword, floating subheading word, candidate term word] (205510)

24 general medic*.mp. [mp=title, abstract, heading word, drug trade name, original title, device manufacturer, drug manufacturer, device trade name, keyword, floating subheading word, candidate term word] (22708)

25 exp family medicine/ (11673)

26 family medic*.mp. [mp=title, abstract, heading word, drug trade name, original title, device manufacturer, drug manufacturer, device trade name, keyword, floating subheading word, candidate term word] (20287)

27 family practi*.mp. [mp=title, abstract, heading word, drug trade name, original title, device manufacturer, drug manufacturer, device trade name, keyword, floating subheading word, candidate term word] (12825)

28 family physician*.mp. [mp=title, abstract, heading word, drug trade name, original title, device manufacturer, drug manufacturer, device trade name, keyword, floating subheading word, candidate term word] (19317)

29 family doctor*.mp. [mp=title, abstract, heading word, drug trade name, original title, device manufacturer, drug manufacturer, device trade name, keyword, floating subheading word, candidate term word] (6797)

30 1 or 2 or 3 or 4 or 5 or 6 or 7 or 8 (591107)

31 9 or 10 or 11 or 12 or 13 or 14 or 15 or 16 or 17 (73395)

32 18 or 19 or 20 or 21 or 22 or 23 or 24 or 25 or 26 or 27 or 28 or 29 (437307)

33 30 and 31 and 32 (5451)

34 limit 33 to randomized controlled trial (487)

35 33 not 34 (4964)

***************************

Medline 7 April 2021

Via Ovid

Database: Ovid MEDLINE(R) and Epub Ahead of Print, In-Process, In-Data-Review & Other Non-Indexed Citations and Daily <1946 to April 06, 2021>

Search Strategy:

--------------------------------------------------------------------------------

1 exp Smoking/ (150989)

2 exp Tobacco Smoking/ (3969)

3 exp Cigarette Smoking/ (2459)

4 exp Tobacco/ (31718)

5 exp "Tobacco Use"/ (5915)

6 exp "Tobacco Use Disorder"/ (11528)

7 tobacco.mp. [mp=title, abstract, original title, name of substance word, subject heading word, floating sub-heading word, keyword heading word, organism supplementary concept word, protocol supplementary concept word, rare disease supplementary concept word, unique identifier, synonyms] (133865)

8 smoking.mp. [mp=title, abstract, original title, name of substance word, subject heading word, floating sub-heading word, keyword heading word, organism supplementary concept word, protocol supplementary concept word, rare disease supplementary concept word, unique identifier, synonyms] (294977)

9 cigarett*.mp. [mp=title, abstract, original title, name of substance word, subject heading word, floating sub-heading word, keyword heading word, organism supplementary concept word, protocol supplementary concept word, rare disease supplementary concept word, unique identifier, synonyms] (75715)

10 exp Smoking Cessation/ (29636)

11 smoking cessation.mp. [mp=title, abstract, original title, name of substance word, subject heading word, floating sub-heading word, keyword heading word, organism supplementary concept word, protocol supplementary concept word, rare disease supplementary concept word, unique identifier, synonyms] (40497)

12 quit* smoking.mp. [mp=title, abstract, original title, name of substance word, subject heading word, floating sub-heading word, keyword heading word, organism supplementary concept word, protocol supplementary concept word, rare disease supplementary concept word, unique identifier, synonyms] (8424)

13 stop* smoking.mp. [mp=title, abstract, original title, name of substance word, subject heading word, floating sub-heading word, keyword heading word, organism supplementary concept word, protocol supplementary concept word, rare disease supplementary concept word, unique identifier, synonyms] (4893)

14 tobacco cessation.mp. [mp=title, abstract, original title, name of substance word, subject heading word, floating sub-heading word, keyword heading word, organism supplementary concept word, protocol supplementary concept word, rare disease supplementary concept word, unique identifier, synonyms] (2497)

15 smoking abstinence.mp. [mp=title, abstract, original title, name of substance word, subject heading word, floating sub-heading word, keyword heading word, organism supplementary concept word, protocol supplementary concept word, rare disease supplementary concept word, unique identifier, synonyms] (1668)

16 quit attempt*.mp. [mp=title, abstract, original title, name of substance word, subject heading word, floating sub-heading word, keyword heading word, organism supplementary concept word, protocol supplementary concept word, rare disease supplementary concept word, unique identifier, synonyms] (2721)

17 quit date*.mp. [mp=title, abstract, original title, name of substance word, subject heading word, floating sub-heading word, keyword heading word, organism supplementary concept word, protocol supplementary concept word, rare disease supplementary concept word, unique identifier, synonyms] (625)

18 exp Primary Health Care/ (166713)

19 primary care.mp. [mp=title, abstract, original title, name of substance word, subject heading word, floating sub-heading word, keyword heading word, organism supplementary concept word, protocol supplementary concept word, rare disease supplementary concept word, unique identifier, synonyms] (124282)

20 primary medic*.mp. [mp=title, abstract, original title, name of substance word, subject heading word, floating sub-heading word, keyword heading word, organism supplementary concept word, protocol supplementary concept word, rare disease supplementary concept word, unique identifier, synonyms] (2138)

21 primary health*.mp. [mp=title, abstract, original title, name of substance word, subject heading word, floating sub-heading word, keyword heading word, organism supplementary concept word, protocol supplementary concept word, rare disease supplementary concept word, unique identifier, synonyms] (102377)

22 exp General Practice/ (75681)

23 general practi*.mp. [mp=title, abstract, original title, name of substance word, subject heading word, floating sub-heading word, keyword heading word, organism supplementary concept word, protocol supplementary concept word, rare disease supplementary concept word, unique identifier, synonyms] (94408)

24 general medic*.mp. [mp=title, abstract, original title, name of substance word, subject heading word, floating sub-heading word, keyword heading word, organism supplementary concept word, protocol supplementary concept word, rare disease supplementary concept word, unique identifier, synonyms] (14767)

25 exp Family Practice/ (65514)

26 family medic*.mp. [mp=title, abstract, original title, name of substance word, subject heading word, floating sub-heading word, keyword heading word, organism supplementary concept word, protocol supplementary concept word, rare disease supplementary concept word, unique identifier, synonyms] (12281)

27 family practi*.mp. [mp=title, abstract, original title, name of substance word, subject heading word, floating sub-heading word, keyword heading word, organism supplementary concept word, protocol supplementary concept word, rare disease supplementary concept word, unique identifier, synonyms] (70243)

28 family physician*.mp. [mp=title, abstract, original title, name of substance word, subject heading word, floating sub-heading word, keyword heading word, organism supplementary concept word, protocol supplementary concept word, rare disease supplementary concept word, unique identifier, synonyms] (15119)

29 family doctor*.mp. [mp=title, abstract, original title, name of substance word, subject heading word, floating sub-heading word, keyword heading word, organism supplementary concept word, protocol supplementary concept word, rare disease supplementary concept word, unique identifier, synonyms] (4794)

30 1 or 2 or 3 or 4 or 5 or 6 or 7 or 8 or 9 (369611)

31 10 or 11 or 12 or 13 or 14 or 15 or 16 or 17 (45754)

32 18 or 19 or 20 or 21 or 22 or 23 or 24 or 25 or 26 or 27 or 28 or 29 (388463)

33 30 and 31 and 32 (3356)

34 limit 33 to randomized controlled trial (493)

35 33 not 34 (2863)

***************************

APA PsycInfo 7 April 2021

Via Ovid

Database: APA PsycInfo <1806 to March Week 5 2021>

Search Strategy:

--------------------------------------------------------------------------------

1 exp Tobacco Smoking/ (34023)

2 exp "Tobacco Use Disorder"/ (239)

3 smoking.mp. [mp=title, abstract, heading word, table of contents, key concepts, original title, tests & measures, mesh] (59809)

4 tobacco.mp. [mp=title, abstract, heading word, table of contents, key concepts, original title, tests & measures, mesh] (43684)

5 cigarett*.mp. [mp=title, abstract, heading word, table of contents, key concepts, original title, tests & measures, mesh] (22468)

6 exp Smoking Cessation/ (13661)

7 smoking cessation.mp. [mp=title, abstract, heading word, table of contents, key concepts, original title, tests & measures, mesh] (18659)

8 quit* smoking.mp. [mp=title, abstract, heading word, table of contents, key concepts, original title, tests & measures, mesh] (4301)

9 stop* smoking.mp. [mp=title, abstract, heading word, table of contents, key concepts, original title, tests & measures, mesh] (1664)

10 tobacco cessation.mp. [mp=title, abstract, heading word, table of contents, key concepts, original title, tests & measures, mesh] (1113)

11 smoking abstinence.mp. [mp=title, abstract, heading word, table of contents, key concepts, original title, tests & measures, mesh] (1289)

12 quit attempt*.mp. [mp=title, abstract, heading word, table of contents, key concepts, original title, tests & measures, mesh] (1952)

13 quit date*.mp. [mp=title, abstract, heading word, table of contents, key concepts, original title, tests & measures, mesh] (419)

14 exp Primary Health Care/ (18961)

15 primary care.mp. [mp=title, abstract, heading word, table of contents, key concepts, original title, tests & measures, mesh] (32989)

16 primary medic*.mp. [mp=title, abstract, heading word, table of contents, key concepts, original title, tests & measures, mesh] (570)

17 primary health*.mp. [mp=title, abstract, heading word, table of contents, key concepts, original title, tests & measures, mesh] (25808)

18 exp General Practitioners/ (5993)

19 general practi*.mp. [mp=title, abstract, heading word, table of contents, key concepts, original title, tests & measures, mesh] (15647)

20 general medic*.mp. [mp=title, abstract, heading word, table of contents, key concepts, original title, tests & measures, mesh] (4741)

21 exp Family Medicine/ (1252)

22 family medic*.mp. [mp=title, abstract, heading word, table of contents, key concepts, original title, tests & measures, mesh] (2830)

23 exp Family Physicians/ (1557)

24 family practi*.mp. [mp=title, abstract, heading word, table of contents, key concepts, original title, tests & measures, mesh] (6648)

25 family physician*.mp. [mp=title, abstract, heading word, table of contents, key concepts, original title, tests & measures, mesh] (3148)

26 family doctor*.mp. [mp=title, abstract, heading word, table of contents, key concepts, original title, tests & measures, mesh] (769)

27 1 or 2 or 3 or 4 or 5 (68742)

28 6 or 7 or 8 or 9 or 10 or 11 or 12 or 13 (20188)

29 14 or 15 or 16 or 17 or 18 or 19 or 20 or 21 or 22 or 23 or 24 or 25 or 26 (61794)

30 27 and 28 and 29 (955)

***************************

CINAHL 7 April 2021

<http://search.ebscohost.com/login.aspx?profile=ehost&defaultdb=cin20&authtype=ip,shib&custid=s5003934>

‘Direct Export in RIS Format (e.g. CITAVI, EasyBib, EndNote, ProCite, Reference Manager, Zotero)’

Wednesday, April 07, 2021 9:13:43 AM

# Query Limiters/Expanders Last Run Via Results

S30 S27 AND S28 AND S29 Expanders - Apply equivalent subjects

Search modes - Boolean/Phrase Interface - EBSCOhost Research Databases

Search Screen - Advanced Search

Database - CINAHL 1,861

S29 S15 OR S16 OR S17 OR S18 OR S19 OR S20 OR S21 OR S22 OR S23 OR S24 OR S25 OR S26 Expanders - Apply equivalent subjects

Search modes - Boolean/Phrase Interface - EBSCOhost Research Databases

Search Screen - Advanced Search

Database - CINAHL 163,724

S28 S6 OR S7 OR S8 OR S9 OR S10 OR S11 OR S12 OR S13 OR S14 Expanders - Apply equivalent subjects

Search modes - Boolean/Phrase Interface - EBSCOhost Research Databases

Search Screen - Advanced Search

Database - CINAHL 29,163

S27 S1 OR S2 OR S3 OR S4 OR S5 Expanders - Apply equivalent subjects

Search modes - Boolean/Phrase Interface - EBSCOhost Research Databases

Search Screen - Advanced Search

Database - CINAHL 126,242

S26 "family doctor*" Expanders - Apply equivalent subjects

Search modes - Boolean/Phrase Interface - EBSCOhost Research Databases

Search Screen - Advanced Search

Database - CINAHL 12,882

S25 "family physician*" Expanders - Apply equivalent subjects

Search modes - Boolean/Phrase Interface - EBSCOhost Research Databases

Search Screen - Advanced Search

Database - CINAHL 16,195

S24 "family practi*" Expanders - Apply equivalent subjects

Search modes - Boolean/Phrase Interface - EBSCOhost Research Databases

Search Screen - Advanced Search

Database - CINAHL 27,402

S23 "family medic*" Expanders - Apply equivalent subjects

Search modes - Boolean/Phrase Interface - EBSCOhost Research Databases

Search Screen - Advanced Search

Database - CINAHL 4,964

S22 "general medic*" Expanders - Apply equivalent subjects

Search modes - Boolean/Phrase Interface - EBSCOhost Research Databases

Search Screen - Advanced Search

Database - CINAHL 5,173

S21 "general practi*" Expanders - Apply equivalent subjects

Search modes - Boolean/Phrase Interface - EBSCOhost Research Databases

Search Screen - Advanced Search

Database - CINAHL 31,588

S20 (MH "Family Practice") Expanders - Apply equivalent subjects

Search modes - Boolean/Phrase Interface - EBSCOhost Research Databases

Search Screen - Advanced Search

Database - CINAHL 25,431

S19 "primary health*" Expanders - Apply equivalent subjects

Search modes - Boolean/Phrase Interface - EBSCOhost Research Databases

Search Screen - Advanced Search

Database - CINAHL 73,983

S18 "primary medic*" Expanders - Apply equivalent subjects

Search modes - Boolean/Phrase Interface - EBSCOhost Research Databases

Search Screen - Advanced Search

Database - CINAHL 684

S17 (MH "Physicians, Family") Expanders - Apply equivalent subjects

Search modes - Boolean/Phrase Interface - EBSCOhost Research Databases

Search Screen - Advanced Search

Database - CINAHL 20,676

S16 "primary care" Expanders - Apply equivalent subjects

Search modes - Boolean/Phrase Interface - EBSCOhost Research Databases

Search Screen - Advanced Search

Database - CINAHL 89,309

S15 (MH "Primary Health Care") Expanders - Apply equivalent subjects

Search modes - Boolean/Phrase Interface - EBSCOhost Research Databases

Search Screen - Advanced Search

Database - CINAHL 66,100

S14 "quit date*" Expanders - Apply equivalent subjects

Search modes - Boolean/Phrase Interface - EBSCOhost Research Databases

Search Screen - Advanced Search

Database - CINAHL 334

S13 "quit attempt*" Expanders - Apply equivalent subjects

Search modes - Boolean/Phrase Interface - EBSCOhost Research Databases

Search Screen - Advanced Search

Database - CINAHL 1,752

S12 "smoking abstinence" Expanders - Apply equivalent subjects

Search modes - Boolean/Phrase Interface - EBSCOhost Research Databases

Search Screen - Advanced Search

Database - CINAHL 874

S11 "stop* smoking" Expanders - Apply equivalent subjects

Search modes - Boolean/Phrase Interface - EBSCOhost Research Databases

Search Screen - Advanced Search

Database - CINAHL 15,000

S10 "quit* smoking" Expanders - Apply equivalent subjects

Search modes - Boolean/Phrase Interface - EBSCOhost Research Databases

Search Screen - Advanced Search

Database - CINAHL 15,979

S9 "smoking cessation" Expanders - Apply equivalent subjects

Search modes - Boolean/Phrase Interface - EBSCOhost Research Databases

Search Screen - Advanced Search

Database - CINAHL 27,315

S8 "tobacco cessation" Expanders - Apply equivalent subjects

Search modes - Boolean/Phrase Interface - EBSCOhost Research Databases

Search Screen - Advanced Search

Database - CINAHL 1,626

S7 (MH "Smoking Cessation Programs") Expanders - Apply equivalent subjects

Search modes - Boolean/Phrase Interface - EBSCOhost Research Databases

Search Screen - Advanced Search

Database - CINAHL 2,479

S6 (MM "Smoking Cessation") Expanders - Apply equivalent subjects

Search modes - Boolean/Phrase Interface - EBSCOhost Research Databases

Search Screen - Advanced Search

Database - CINAHL 14,065

S5 "tobacco" Expanders - Apply equivalent subjects

Search modes - Boolean/Phrase Interface - EBSCOhost Research Databases

Search Screen - Advanced Search

Database - CINAHL 35,423

S4 "cigarett*" Expanders - Apply equivalent subjects

Search modes - Boolean/Phrase Interface - EBSCOhost Research Databases

Search Screen - Advanced Search

Database - CINAHL 24,757

S3 "smoking" Expanders - Apply equivalent subjects

Search modes - Boolean/Phrase Interface - EBSCOhost Research Databases

Search Screen - Advanced Search

Database - CINAHL 111,904

S2 (MH "Tobacco") Expanders - Apply equivalent subjects

Search modes - Boolean/Phrase Interface - EBSCOhost Research Databases

Search Screen - Advanced Search

Database - CINAHL 7,850

S1 (MH "Smoking+") Expanders - Apply equivalent subjects

Search modes - Boolean/Phrase Interface - EBSCOhost Research Databases

Search Screen - Advanced Search

Database - CINAHL 73,196

--------------------------------------------------------------------------------

Global Health 7 April 2021

Via Ovid

Database: Global Health <1973 to 2021 Week 13>

Search Strategy:

--------------------------------------------------------------------------------

1 exp smoking/ (1417)

2 exp tobacco smoking/ (51559)

3 exp tobacco/ (21785)

4 tobacco.mp. [mp=abstract, title, original title, broad terms, heading words, identifiers, cabicodes] (60470)

5 smoking.mp. [mp=abstract, title, original title, broad terms, heading words, identifiers, cabicodes] (82809)

6 cigarett*.mp. [mp=abstract, title, original title, broad terms, heading words, identifiers, cabicodes] (26193)

7 exp smoking cessation/ (6536)

8 smoking cessation.mp. [mp=abstract, title, original title, broad terms, heading words, identifiers, cabicodes] (9629)

9 quit* smoking.mp. [mp=abstract, title, original title, broad terms, heading words, identifiers, cabicodes] (3067)

10 stop* smoking.mp. [mp=abstract, title, original title, broad terms, heading words, identifiers, cabicodes] (1177)

11 tobacco cessation.mp. [mp=abstract, title, original title, broad terms, heading words, identifiers, cabicodes] (873)

12 smoking abstinence.mp. [mp=abstract, title, original title, broad terms, heading words, identifiers, cabicodes] (492)

13 quit attempt*.mp. [mp=abstract, title, original title, broad terms, heading words, identifiers, cabicodes] (1199)

14 quit date*.mp. [mp=abstract, title, original title, broad terms, heading words, identifiers, cabicodes] (165)

15 exp primary health care/ (16265)

16 primary care.mp. [mp=abstract, title, original title, broad terms, heading words, identifiers, cabicodes] (19465)

17 primary medic*.mp. [mp=abstract, title, original title, broad terms, heading words, identifiers, cabicodes] (324)

18 primary health*.mp. [mp=abstract, title, original title, broad terms, heading words, identifiers, cabicodes] (23841)

19 general pract*.mp. [mp=abstract, title, original title, broad terms, heading words, identifiers, cabicodes] (11543)

20 general medic*.mp. [mp=abstract, title, original title, broad terms, heading words, identifiers, cabicodes] (1509)

21 exp general practitioners/ (4458)

22 family medic*.mp. [mp=abstract, title, original title, broad terms, heading words, identifiers, cabicodes] (1404)

23 family practi*.mp. [mp=abstract, title, original title, broad terms, heading words, identifiers, cabicodes] (820)

24 family physician*.mp. [mp=abstract, title, original title, broad terms, heading words, identifiers, cabicodes] (1718)

25 family doctor*.mp. [mp=abstract, title, original title, broad terms, heading words, identifiers, cabicodes] (653)

26 1 or 2 or 3 or 4 or 5 or 6 (91239)

27 7 or 8 or 9 or 10 or 11 or 12 or 13 or 14 (11400)

28 15 or 16 or 17 or 18 or 19 or 20 or 21 or 22 or 23 or 24 or 25 (45313)

29 26 and 27 and 28 (652)

***************************

Social Policy and Practice 7 April 2021

Via Ovid

Database: Social Policy and Practice <202101>

Search Strategy:

--------------------------------------------------------------------------------

1 smoking.mp. [mp=abstract, title, publication type, heading word, accession number] (1675)

2 tobacco.mp. [mp=abstract, title, publication type, heading word, accession number] (566)

3 cigarett*.mp. [mp=abstract, title, publication type, heading word, accession number] (261)

4 smoking cessation.mp. [mp=abstract, title, publication type, heading word, accession number] (162)

5 quit* smoking.mp. [mp=abstract, title, publication type, heading word, accession number] (24)

6 stop* smoking.mp. [mp=abstract, title, publication type, heading word, accession number] (56)

7 tobacco cessation.mp. [mp=abstract, title, publication type, heading word, accession number] (3)

8 smoking abstinence.mp. [mp=abstract, title, publication type, heading word, accession number] (3)

9 quit attempt*.mp. [mp=abstract, title, publication type, heading word, accession number] (5)

10 quit date*.mp. [mp=abstract, title, publication type, heading word, accession number] (2)

11 primary care.mp. [mp=abstract, title, publication type, heading word, accession number] (5457)

12 primary medic*.mp. [mp=abstract, title, publication type, heading word, accession number] (44)

13 primary health*.mp. [mp=abstract, title, publication type, heading word, accession number] (1050)

14 general practi*.mp. [mp=abstract, title, publication type, heading word, accession number] (3962)

15 general medic*.mp. [mp=abstract, title, publication type, heading word, accession number] (198)

16 family medic*.mp. [mp=abstract, title, publication type, heading word, accession number] (37)

17 family practi*.mp. [mp=abstract, title, publication type, heading word, accession number] (200)

18 family physician*.mp. [mp=abstract, title, publication type, heading word, accession number] (55)

19 family doctor*.mp. [mp=abstract, title, publication type, heading word, accession number] (73)

20 1 or 2 or 3 (1906)

21 4 or 5 or 6 or 7 or 8 or 9 or 10 (209)

22 11 or 12 or 13 or 14 or 15 or 16 or 17 or 18 or 19 (8793)

23 20 and 21 and 22 (28)

***************************

***************************

ASSIA Applied Social Sciences Index and Abstracts 7 April 2021

Via ProQuest

(ab,ti,su(smoking) OR ab,ti,su(cigarett[*20]) OR ab,ti,su(tobacco) OR MAINSUBJECT.EXACT.EXPLODE("Smoking"))

AND (ab,ti,su(smoking cessation) OR ab,ti,su(quit[*20] smoking) OR ab,ti,su(stop[*20] smoking) OR ab,ti,su(tobacco cessation) OR ab,ti,su(smoking abstinence) OR ab,ti,su(quit attempt[*20]) OR ab,ti,su(quit date[*20]) OR MAINSUBJECT.EXACT.EXPLODE("Cessation"))

AND (ab,ti,su(primary care) OR ab,ti,su(primary medic[*20]) OR ab,ti,su(primary health[*20]) OR ab,ti,su(general medic[*20]) OR ab,ti,su(general practi[*20]) OR ab,ti,su(family practi[*20]) OR ab,ti,su(family medic[*20]) OR ab,ti,su(family physician[*20]) OR ab,ti,su(family doctor[*20]) OR MAINSUBJECT.EXACT.EXPLODE("Primary health care"))

830 results

***************************

OpenGrey 7 April 2021

<http://www.opengrey.eu/>

“GreyNet has recently archived OpenGrey in its collection of research data housed in the DANS EASY Archive <https://doi.org/10.17026/dans-xtf-47w5>. OpenGrey will be shutdown before summer.”

(smoking OR tobacco OR cigarett*) AND (general medic* OR general practi* OR primary care OR primary health* OR primary medic* OR family practi* OR family medic* OR family physician* OR family doctor*)

- Exclude ‘thesis’

11 results (other 272 results were PhD theses)

***************************

Social Care Online 7 April 2021

<https://www.scie-socialcareonline.org.uk/>

(smoking OR tobacco OR cigarett*) 748

(general medic* OR general practi* OR primary care OR primary health* OR primary medic* OR family practi* OR family medic* OR family physician* OR family doctor*) 10,809

(smoking cessation OR quit* smoking OR stop* smoking OR tobacco cessation OR smoking abstinence OR quit attempt* OR quit date* OR cessation) 178

**Current search (with results shown below)**

- **(New Combined Search:**
  - Smoking concept   [
     -  AllFields:'smoking'
     - OR AllFields:'tobacco'
     - OR AllFields:'cigarett*'
    ]
    **AND**
  - Primary care concept   [
     -  AllFields:'general medic*'
     - OR AllFields:'general practi*'
     - OR AllFields:'primary care'
     - OR AllFields:'primary health*'
     - OR AllFields:'primary medic*'
     - OR AllFields:'family practi*'
     - OR AllFields:'family medic*'
     - OR AllFields:'family physician*'
     - OR AllFields:'family doctor*'
    ]
    **AND**
  - Smoking cessation concept   [
     -  AllFields:'smoking cessation'
     - OR AllFields:'quit* smoking'
     - OR AllFields:'stop* smoking'
     - OR AllFields:'tobacco cessation'
     - OR AllFields:'smoking abstinence'
     - OR AllFields:'quit attempt*'
     - OR AllFields:'quit date*'
     - OR AllFields:'cessation'
    ]

**)**

15 results

***************************

Healthcare Management Information Consortium Database 7 April 2021

Via Ovid

Database: HMIC Health Management Information Consortium <1979 to January 2021>

Search Strategy:

--------------------------------------------------------------------------------

1 exp Smoking/ (3747)

2 exp Tobacco/ (725)

3 exp Cigarette tobacco/ (6)

4 exp Tobacco consumption/ (169)

5 smoking.mp. [mp=title, other title, abstract, heading words] (8418)

6 tobacco.mp. [mp=title, other title, abstract, heading words] (2746)

7 cigarett*.mp. [mp=title, other title, abstract, heading words] (1730)

8 exp Smoking cessation/ (1895)

9 exp Smoking treatment/ (218)

10 smoking cessation.mp. [mp=title, other title, abstract, heading words] (2208)

11 quit* smoking.mp. [mp=title, other title, abstract, heading words] (329)

12 stop* smoking.mp. [mp=title, other title, abstract, heading words] (511)

13 tobacco cessation.mp. [mp=title, other title, abstract, heading words] (36)

14 smoking abstinence.mp. [mp=title, other title, abstract, heading words] (42)

15 quit attempt*.mp. [mp=title, other title, abstract, heading words] (122)

16 quit date*.mp. [mp=title, other title, abstract, heading words] (51)

17 exp primary care/ (22774)

18 primary care.mp. [mp=title, other title, abstract, heading words] (24846)

19 primary medic*.mp. [mp=title, other title, abstract, heading words] (261)

20 primary health*.mp. [mp=title, other title, abstract, heading words] (3603)

21 exp Primary care teams/ (659)

22 exp General practice/ (9229)

23 general practi*.mp. [mp=title, other title, abstract, heading words] (26625)

24 general medic*.mp. [mp=title, other title, abstract, heading words] (2586)

25 family medic*.mp. [mp=title, other title, abstract, heading words] (201)

26 family practi*.mp. [mp=title, other title, abstract, heading words] (1147)

27 exp General practitioners/ (10304)

28 family physician*.mp. [mp=title, other title, abstract, heading words] (267)

29 family doctor*.mp. [mp=title, other title, abstract, heading words] (388)

30 1 or 2 or 3 or 4 or 5 or 6 or 7 (9309)

31 8 or 9 or 10 or 11 or 12 or 13 or 14 or 15 or 16 (2428)

32 17 or 18 or 19 or 20 or 21 or 22 or 23 or 24 or 25 or 26 or 27 or 28 or 29 (48184)

33 30 and 31 and 32 (353)

***************************
