# Supplementary material for: Implementation strategies to increase smoking cessation treatment provision in primary care: a systematic review of observational studies
Source: BMC Prim Care. 2023 Jan 25;24:32. doi: 10.1186/s12875-023-01981-2 (PMC9875430; doi:10.1186/s12875-023-01981-2)
Supplement: Supplementary file 5 — Additional file 5: Appendix 5. Pre-piloted data extraction form fields. [file 12875_2023_1981_MOESM5_ESM.docx]

**Appendix: Pre-piloted data extraction form fields**

- Authors’ information
- Year of publication
- Year(s) the data analysed was collected in
- Country in which intervention was delivered
- Details of the intervention (including duration)
- Description of comparator/control
- Description of the setting/context (e.g. environmental and cultural factors)
- Study design type
- Data collection method (interview, telephone, mail survey, electronic health records)
- Respondent (patient, provider, other: specify)
- Inclusion criteria, including sub-populations
- Characteristics of study participants (age, sex, co‐morbidities, readiness to quit)
- Outcome measures and definitions used (including self-reported or biochemically verified etc), and time point at which they were assessed.
  - Quantitative outcomes:
- number of participants included in analysis
- number of people in each group
- estimate effect with confidence interval
- Explanation offered to explain why certain strategies to increase the provision and uptake of smoking cessation treatment in primary care settings were/were not effective
- Cost effectiveness estimates or economic indicators
- Methods for managing missing data
- Funding and declaration of interest for primary investigators
- Authors' conclusions
