# Supplementary material for: Implementation strategies to increase smoking cessation treatment provision in primary care: a systematic review of observational studies
Source: BMC Prim Care. 2023 Jan 25;24:32. doi: 10.1186/s12875-023-01981-2 (PMC9875430; doi:10.1186/s12875-023-01981-2)
Supplement: Supplementary file 6 — Additional file 6: Appendix 6. Risk of bias assessments. [file 12875_2023_1981_MOESM6_ESM.pdf]

Appendix - Risk of bias assessment

As outlined in the manuscript, The ROBINS-I (Risk Of Bias In Non-randomized Studies of Interventions) tool was used to evaluate the risk of bias in non-randomised observational studies (23–25).
BT performed the risk of bias assessments. After the first five studies were assessed, LB also assessed these, and BT and LB compared ratings. The risk of bias assessment ratings and justifications are included below.

The tool assesses risk of bias in seven domains (23):
•Pre-intervention: (1) bias due to confounding, (2) bias in selection of participants into the study.
•At intervention: (3) bias in classification of interventions.
•Post-intervention: (4) bias due to deviations from intended interventions, (5) bias due to missing data, (6) bias in measurement of outcomes, (7) bias in selection of the reported result.

Then an overall risk of bias rating is decided for each study (23):
•Low risk of bias: The study is comparable to a well performed randomised trial.
•Moderate risk of bias: The study provides sound evidence for a non-randomised study but cannot be considered comparable to a well performed randomised trial.
•Serious risk of bias: The study has some important problems.
•Critical risk of bias: The study is too problematic to provide any useful evidence and should not be included in any synthesis.
•No information: No information on which to base a judgement about risk of bias.

References:

23.Sterne JA, Hernán MA, Reeves BC, Savović J, Berkman ND, Viswanathan M, et al. ROBINS-I: a tool for assessing risk of bias in non-randomised studies of interventions. BMJ. 2016 Oct;355:i4919.
24.Mueller M, D'Addario M, Egger M, Cevallos M, Dekkers O, Mugglin C, et al. Methods to systematically review and meta-analyse observational studies: a systematic scoping review of recommendations. BMC Med Res Methodol. 2018;18(1):44.
25.Sterne JAC, Hernán MA, McAleenan A, Reeves BC, Higgins JPT. Chapter 25: Assessing risk of bias in a non-randomized study. In: Higgins J, Thomas J, Chandler J, Cumpston M, Li T, Page M, et al., editors. Cochrane Handbook for Systematic Reviews of Interventions version 62 (updated February 2021) [Internet]. 2021 [cited 2022 Feb 3]. Available from: https://training.cochrane.org/handbook

First author, year	Bias due to confounding	Support for judgement	Bias in selection of participants into the study	Support for judgement	Bias in classification of interventions	Support for judgement	Bias due to deviations from intended interventions	Support for judgement	Bias due to missing data	Support for judgement	Bias in measurement of outcomes	Support for judgement	Bias in selection of the reported result	Support for judgement	Overall risk of bias	Double rated
Szatkowski, 2016	Moderate	Plausible other events could have affected outcomes but there was no discussion of this in the paper, but authors did use ARIMA (autoregressive integrated moving average) modelling which can account for underlying long-term and/or seasonal trends. Limited time period post-intervention (1 year) (though 12 time points is acceptable for a time series design).	Low	Electronic health records of 12 million patients aged >16 years from 600 GP clinics in the UK, broadly representative of the UK population in terms of the age-sex distribution.	Low	Population and intervention clearly outlined.	Low	Characteristics of GP clinics may have an influence, but these were assumed to be the same pre- and post- the intervention.	Moderate	No mention of missing or excluded data but assumed that the proportion of participants and reasons for missing data are similar pre- and post- intervention.	Low	Outcome measure was the same pre- and post- the intervention. Electronic record of: record of advice to quit, record of referral to the NHS Stop Smoking Service, and prescription for a smoking cessation medication. GPs enter data into electronic records, 'record of advice to quit' could be subjective.	Moderate	Two analyses reported: pre period: Apr 2004 to Mar 2012, post: Apr 2012 to Apr 2013. And pre period: Apr 2010 to Mar 2012, post period: Apr 2012 to Apr 2013. Reporting was transparent, but there was no pre-specified analysis plan.	Moderate	LB
Szatkowski, 2011	Moderate	Primary Care Trust Patient Survey data was standardised by age, sex and Strategic Health Authority (SHA) to match the THIN primary care electronic healthcare records, but no other confounders were mentioned or adjusted for.	Serious	Response rate for the PCT Patient Survey was "47.4%, 45.4% and 38.3%" for the 3 years analysed - this could harbour response bias.	Low	Population and intervention clearly outlined.	Low	This is not expected in observational studies of individuals in routine care.	Moderate	No mention of missing or excluded data but assumed that the proportion of participants and reasons for missing data are similar pre- and post- intervention.	Low	Two different types of the outcome measure are compared (self report by patients vs self report by the GPs)	Moderate	Only one effect estimate is reported (for the whole population). No pre-specified analysis plan.	Serious	LB
Szatkowski, 2010	Serious	This is a descriptive, repeated cross-sectional study, no controlling for confounders or secular trend. Causality cannot be inferred.	Low	Electronic health records from 600 GP clinics in the UK were used, broadly representative of the UK population. Note: the study period is from 1990, it is assumed that THIN was nationally representative in the past too.	Low	Population and intervention clearly outlined.	Low	This is not expected in observational studies of individuals in routine care.	Moderate	No mention of missing or excluded data but assumed that the proportion of participants and reasons for missing data are similar pre- and post- intervention.	Low	Outcome measure was the same pre- and post- the intervention. Electronic record of smoking status.	Moderate	Smoking status recording proportion for whole population per year is reported, no subgroup analyses or adjusted results. No pre-specified analysis plan.	Serious	
Taggar, 2012	Serious	This is a descriptive, repeated cross-sectional study. There is some controlling for confounders but not for any secular trends or extraneous policy events. Causality cannot be inferred.	Low	Electronic health records from 600 GP clinics in the UK were used, broadly representative of the UK population. Note: the study period is from 1990, it is assumed that THIN was nationally representative in the past too.	Low	Population and intervention clearly outlined.	Low	This is not expected in observational studies of individuals in routine care.	Moderate	No mention of missing or excluded data apart from for Townsend category - proportion of patients missing Townsend category is similar in 2002, 2004 and 2008.	Low	Outcome measure was the same pre- and post- the intervention. Electronic record of: smoking status and smoking cessation advice. GPs enter data into electronic records, 'record of cessation advice' could be subjective.	Moderate	Several adjusted analyses were performed but reported and outlined appropriately. No pre-specified analysis plan.	Serious	
Alageel, 2019	Low	Used interrupted time series design, with matched controls, and adjusted for confounding factors.	Low	Patients who received the health check were identified from electronic health records - study authors had no input into the selection of the patients.	Low	Population and intervention clearly outlined.	Low	This is not expected in observational studies of individuals in routine care.	Low	High level of missing data, but attempted to address using last observation carried forward (LOCF) method. Sensitivity analyses reported.	Low	Outcome measure was the same pre- and post- the intervention	Low	Study protocol included and sensitivity analyses reported.	Low	
Coleman, 2007	Serious	This is a descriptive, repeated cross-sectional study, no controlling for confounders or secular trend. Causality cannot be inferred.	Low	Electronic health records from GP clinics in the UK were used, broadly representative of the UK population. Note: the study period is from 1990, it is assumed that THIN was nationally representative in the past too.	Low	Population and intervention clearly outlined.	Low	This is not expected in observational studies of individuals in routine care.	Moderate	No mention of missing or excluded data but assumed that the proportion of participants and reasons for missing data are similar pre- and post- intervention.	Low	Outcome measure was the same pre- and post- the intervention. Electronic record of: smoking status and smoking cessation advice. GPs enter data into electronic records, 'record of cessation advice' could be subjective.	Moderate	Results reported against their relevant target groups of interest. No pre-specified analysis plan.	Serious	
Szatkowski, 2011	Moderate	Plausible other events could have affected outcomes but there was no discussion of this in the paper, but authors did use ARIMA (autoregressive integrated moving average) modelling which can account for underlying long-term and/or seasonal trends. Sufficient pre- and post- intervention points included in the analyses. Several relevant confounding factors adjusted for.	Low	Electronic health records GP clinics in the UK, broadly representative of the UK population in terms of the age-sex distribution. Note: the study period is from 2000, it is assumed that THIN was nationally representative in the past too.	Low	Population and intervention clearly outlined.	Low	Unlikely that the policy was implemented differently nationally.	Moderate	No mention of missing or excluded data but assumed that the proportion of participants and reasons for missing data are similar pre- and post- intervention.	Low	Outcome measure was the same pre- and post- the intervention. Electronic record of prescriptions issued is a robust, objective outcome measure.	Moderate	The paper reports a relevant range of analyses of different anticipatory and lag effect of the policy. No pre-specified analysis plan.	Moderate	
Langley, 2011	Moderate	Plausible other events could have affected outcomes. But authors did use segmented regression analysis with generalized additive mixed models (GAMM) to account for the secular trend.	Low	Electronic health records GP clinics in the UK, broadly representative of the UK population in terms of the age-sex distribution. Note: the study period is from 2002, it is assumed that THIN was nationally representative in the past too.	Low	Population and intervention clearly outlined.	Low	This is not expected in observational studies of individuals in routine care.	Moderate	No mention of missing or excluded data but assumed that the proportion of participants and reasons for missing data are similar pre- and post- intervention.	Low	Outcome measure was the same pre- and post- the intervention. Electronic record of NRT prescriptions issued is a robust, objective outcome measure.	Moderate	Results reported against their relevant target groups of interest. No pre-specified analysis plan.	Moderate	
Langley, 2012	Moderate	Plausible other events could have affected outcomes. But authors did use segmented regression analysis with generalized additive mixed models (GAMM) to account for the secular trend.	Low	Electronic health records GP clinics in the UK, broadly representative of the UK population in terms of the age-sex distribution. Note: the study period is from 2002, it is assumed that THIN was nationally representative in the past too.	Low	Population and intervention clearly outlined.	Low	This is not expected in observational studies of individuals in routine care.	Moderate	No mention of missing or excluded data but assumed that the proportion of participants and reasons for missing data are similar pre- and post- intervention.	Low	Outcome measure was the same pre- and post- the intervention. Electronic record of prescriptions issued is a robust, objective outcome measure.	Moderate	Results reported against their relevant target groups of interest. No pre-specified analysis plan.	Moderate	
Bailey, 2017	Moderate	Relevant confounders identified and adjusted for. "Models were adjusted for known variables associated with smoking rates/assistance: race/ethnicity, gender, age category, household percentage of federal povery level (below 100% vs over 100%), insurance status at the majority of visits (insured versus uninsured), number of visits in the measurement year, and comorbid diseases (hypertension, lipid disorder, asthma/chronic obstructive pulmonary disease, cancer diagnosis, diabetes, psychiatric diagnosis, and substance abuse diagnosis, excluding tobacco use disorders). Service area of the patient's primary clinic was included as a fixed effect to adjust for potential differences between health centers. The authors did not adjust for urban versus rural health center as over 90% had a ZIP code in an urban setting across years, as identified using rural-urban commuting area codes."	Low	Electronic health records of the same clinics, pre- and post- intervention.	Low	Population and intervention clearly outlined.	Low	This is not expected in observational studies of individuals in routine care.	Moderate	No mention of missing or excluded data apart from for Household Income. The proportion of patients missing this variable is similar across the study period.	Low	Outcome measure was the same pre- and post- the intervention. GPs enter data into electronic records, 'smoking counselling given' could be subjective.	Moderate	Unadjusted and adjusted odds ratios are reported transparently. No pre-specified analysis plan.	Moderate	LB
Szatkowski, 2021	Moderate	Plausible other events could have affected outcomes. But authors did use segmented regression and tested for autocorrelation to account for the secular trend. Did not control for other confounders.	Low	Electronic health records GP clinics in the UK, representative of the UK population.	Serious	Intervention is not well defined. The "transfer of public health budgets from the NHS to local authorities since 2013" is not well defined and it is unclear if the intervention was the 'same' for all local authorities nationally.	Moderate	Due to the lack of clarity around the classification of the intervention, the time the policy was implemented may have differed between local authorities nationally.	Moderate	No mention of missing or excluded data but assumed that the proportion of participants and reasons for missing data are similar pre- and post- intervention.	Low	Outcome measure was the same pre- and post- the intervention. Electronic record of NRT prescriptions issued is a robust, objective outcome measure.	Moderate	Only one effect estimate is reported (change to prescription rate per whole population over time), but no indication that any sub-group analyses were excluded on purpose. Reporting was transparent, but no pre-specified analysis plan.	Serious	
Langley, 2011	Moderate	Plausible other events could have affected outcomes, like smokefree legislation in 2007, but authors included this in their model. Authors used ARIMA (autoregressive integrated moving average) modelling which can account for underlying long-term and/or seasonal trends. Limited number of data points in between the two interventions investigated.	Low	Electronic health records GP clinics in the UK, representative of the UK population.	Low	Population and intervention clearly outlined.	Low	This is not expected in observational studies of individuals in routine care.	Moderate	No mention of missing or excluded data but assumed that the proportion of participants and reasons for missing data are similar pre- and post- intervention.	Low	Outcome measure was the same pre- and post- the intervention. Electronic record of NRT prescriptions issued is a robust, objective outcome measure.	Moderate	Only one effect estimate is reported (change to prescription rate per whole population over time), but no indication that any sub-group analyses were excluded on purpose. Reporting was transparent, but no pre-specified analysis plan.	Moderate	
Dhalwani, 2013	Serious	Examined several confounding factors: age, socioeconomic status, BMI, asthma, hypertension, diabetes, mental illness. But did not account for other plausible other events which could have affected outcomes, or the underlying secular trend.	Low	Electronic health records GP clinics in the UK, representative of the UK population.	Low	Population and intervention clearly outlined.	Low	This is not expected in observational studies of individuals in routine care.	Moderate	Missing data outlined. Data were reasonably complete. Although it was not stated whether missing data was missing at random, "missing data for Townsend quintile and BMI were included as separate categories in the analyses".	Low	Outcome measure was the same pre- and post- the intervention. Electronic record of: smoking status is a robust outcome measure.	Moderate	Methods was transparent regarding which confounding characteristics were included in the regression model, but there is no overall pre- and post- odds ratio reported. No pre-specified analysis plan.	Serious	
Hardy, 2014	Serious	Examined several confounding factors: age, socioeconomic status, BMI, asthma, hypertension, diabetes, mental illness. But did not account for other plausible other events which could have affected outcomes, or the underlying secular trend.	Low	Electronic health records GP clinics in the UK, representative of the UK population.	Low	Population and intervention clearly outlined.	Low	This is not expected in observational studies of individuals in routine care.	Moderate	Missing data outlined. Data were reasonably complete. Although it was not stated whether missing data was missing at random, "missing data for Townsend quintile and BMI were included as separate categories in the analyses".	Low	Outcome measure was the same pre- and post- the intervention. Electronic record of: smoking cessation advice. GPs enter data into electronic records, 'record of cessation advice' could be subjective.	Moderate	Methods was transparent regarding which confounding characteristics were included in the regression model, but there is no overall pre- and post- odds ratio reported. Only post-intervention odds ratios reported for the individual characteristics. But both adjusted and unadjusted odds ratios are reported. No pre-specified analysis plan.	Serious	
Farley, 2017	Low	Examined several confounding factors and used a control group. Causation cannot be inferred but the study is comparable to a well-performed randomized trial with regard to this domain.	Low	Electronic health records GP clinics in the UK, representative of the UK population.	Low	Population and intervention clearly outlined.	Low	This is not expected in observational studies of individuals in routine care.	Low	No direct mention of missing data but authors are transparent about the completeness of smoking status recording and have matched this to their control group.	Low	Outcome measure was the same pre- and post- the intervention. Electronic record of: record of smoking status (including change of status to indicate smoking cessation after one year post-diagnosis), record of advice to quit, prescription for a smoking cessation medication. GPs enter data into electronic records, 'record of advice to quit' could be subjective.	Low	Appropriate outcome measures reported, study protocol included.	Low	
Dhalwani, 2014	Serious	Examined several confounding factors. But did not account for other plausible other events which could have affected outcomes, or the underlying secular trend.	Low	Electronic health records GP clinics in the UK, representative of the UK population.	Low	Population and intervention clearly outlined.	Low	This is not expected in observational studies of individuals in routine care.	Moderate	Data were reasonably complete. Although it was not stated whether missing data was missing at random, missing data for Townsend score and BMI were included as separate categories in the analyses.	Low	Outcome measure was the same pre- and post- the intervention. Electronic record of NRT prescriptions issued is a robust, objective outcome measure.	Moderate	The overall population ("NRT prescribing in all pregnancies") effect is reported but no pre-specified analysis plan.	Serious	
Tahrani, 2007	Serious	Plausible other events could have affected outcomes but there was no adjustment for these in analyses. Did not control for confounders. "Although the clinical quality data suggest that there has been a real and important health gain, this could have resulted from healthcare initiatives separate from the QOF, such as national and international guidelines regarding cholesterol, blood pressure and blood glucose control targets." Confounding factors re patient characteristics were not reported but the pre-, post-design of the study potentially minimises their effect.	Low	Effort made to control for selection bias: "To remove a potential source of bias and to be able to compare the QOF data with the national diabetes audit, the proportion of patients achieving each quality indicator in each practice out of the total number of patients on the diabetes register in that practice rather than the denominators provided by the practices, was calculated. These denominators provided by practices take into account exception reporting and, as a result, any possible bias of exception reporting from the study's data has been removed."	Moderate	Population and intervention described. Time period of implementation of the intervention not 100% clear. For smoking cessation advice outcome measure, the pre-intervention data is from October 2004 instead of April 2004 - this is not transparently discussed.	Low	This is not expected in observational studies of individuals in routine care.	Moderate	No mention of missing or excluded data but assumed that the proportion of participants and reasons for missing data are similar pre- and post- intervention.	Low	Outcome measure was the same pre- and post- the intervention. Electronic record of: a patient's smoking status, smoking cessation advice. GPs enter data into electronic records, 'smoking cessation advice' could be subjective.	Moderate	Only one effect estimate is reported (for the patient population). No pre-specified analysis plan.	Serious	LB
Mullins, 2009	Serious	Plausible other events could have affected outcomes but there was no discussion of this in the paper. Did not control for gender or race, which is a confounding factor in smoking cessation therapy provision. "These populations were seen during two different periods of time; therefore, it is possible that other factors contributed to differences between the groups (such as TV commercials, or more pharmacotherapy publicity)."	Serious	Electronic health records of the same clinic used, pre- and post- intervention. Paper slightly unclear about inclusion. "Patients without a recorded smoking history were excluded." "Patients were defined as nonsmokers if smoking status flow sheet values were "quit," "never," or if no value was recorded." Excluded patients without a recorded smoking history could have a significant effect on their measured outcomes. Also, "A limitation of our study is an internal flaw of the EMR, which is designed for real-time performance improvement assessment, not for retrospective research. Because of this program design, inquiries searching for patients seen during a particular time period using the parameter "date of last office visit" overestimate the number of patients seen in the most recent time frame. For example, if a patient was seen in 2006 and in 2007, this individual would only appear under "date of last office visit" in 2007. Effectively, this may underestimate the improvement seen from our preintervention group, as the patients seen both before and after the intervention are only being counted in the postintervention group."	Moderate	Population and intervention described. Time period of implementation of the intervention not 100% clear. Also, post-intervention data is actually 'during the intervention'.	Low	This is not expected in observational studies of individuals in routine care.	No information	No mention of missing or excluded data.	Low	Outcome measure was the same pre- and post- the intervention. Electronic record of: smoking status, tobacco cessation counselling. GPs enter data into electronic records, 'record of counselled to quit' could be subjective.	Moderate	Only one effect estimate is reported (for the whole patient population who "had an office visit on or after July 1, 2006" and had a "recorded smoking history", pre- and 'during' the intervention). No pre-specified analysis plan.	Serious	LB
Tilson, 2004	Serious	This is a descriptive, repeated cross-sectional study, no controlling for confounders or secular trend. Causality cannot be inferred.	Low	General Medical Services Payments Board prescription database (for patients eligible for free medications) was used pre- and post- intervention.	Low	Population and intervention clearly outlined.	Low	This is not expected in observational studies of individuals in routine care.	Moderate	No mention of missing or excluded data but assumed that the proportion of participants and reasons for missing data are similar pre- and post- intervention.	Low	Outcome measure was the same pre- and post- the intervention. Electronic record of NRT prescriptions dispensed is a robust, objective outcome measure.	Moderate	Only one effect estimate is reported (for the eligible patient population nationally). No pre-specified analysis plan.	Serious	
Akman, 2017	Moderate	The two surveys' sampling strategy was appropriate (random and quota) and although there is response bias, the characteristics of the 1993 and 2012 samples is similar.	Serious	2 national GP surveys used. In 1993: 199 doctors responded (response rate: 50%), "doctors working in inner city and urban areas were over-represented". In 2012: "final analysis was conducted on data from 299 doctors (response rate: 42.9%)". Small sample size and relatively low response rate could harbour response bias.	Moderate	Intervention is not well defined. It is unclear if the intervention was the 'same' for all local areas nationally, or if there were various lag times. But overall, the "program was implemented between 2003–2010" and the pre- and post-measurements are from 1993 and 2012.	Moderate	The time the policy was implemented may have differed between local authorities nationally.	Moderate	No mention of missing or excluded data but assumed that the proportion of survey participants and reasons for missing data (non-response) are similar pre- and post- intervention.	Low	"The questions in the 1993 survey on GP service profiles were repeated in 2012 with the purpose of comparing general practice between the two time points. As the questions and the categories of answers that were used in 1993 were copied either literally or only revised slightly without changing the meaning in 2012, the data is deemed to be comparable." However, the outcome measure itself is poorly designed: self reported proportion of "primary care doctors who are usually or almost always involved in given preventive care service (smoking counselling during outpatient clinic)".	Moderate	No pre-specified analysis plan. Study reports outcome from survey questions related to smoking cessation therapy provision.	Serious	

Peterson, 2016	Serious	Each participant has their own pre- and post- measurement. Controlled for physician characteristics retained in the anonymous dataset ("The retained physician variables are age in years, gender, zip code, date of residency graduation, years in practice, and number of recertifications.") Patient characteristics not controlled for. Post-intervention data was categorised by time, but this may have been too short - long term impact of the intervention not assessed. "The variable days to PPM completion was categorized into 7–14 days, 15–30 days, 31–60 days, 61–90 days, and 90+ days to investigate associations between time to complete the PPM and outcomes. Physicians cannot enter postintervention data earlier than 7 days after the intervention is started." 55% took more than 91 days to complete the post-intervention measure, mean time to complete the module was 134.9 days. " After the physician implements their chosen interventions, collection of chart and survey data from the next 10 patients they see with a diagnosis of hypertension is repeated." "we used data collected from 10 patients pre- and post-intervention to assess quality of care; this limited number of patients may not accurately capture the true quality of care delivered by the physician." "We found much higher rates of blood pressure control than what is reported in the literature, which may suggest that physicians "cherry picked" the patients they reported."	Low	"We analyzed data from all hypertension PPMs completed from July 2006 to 2013. Exclusion criteria included physicians with incomplete quality data and those residing outside the United States." Study authors had no input into the selection of the patients.	Low	Population and intervention clearly outlined.	Low	This is not expected in observational studies of individuals in routine care.	Low	Missing data outlined. "We identified 8,028 completed hypertension PPMs, reflecting quality measures for between 80,000 and 160,000 patients. After excluding modules completed by physicians not residing in the United States (n=93), those with missing demographic information (n = 1), those with zip codes that could not be linked to a RUCA code (n = 47), and those with incomplete quality data (n = 568), our final sample was 7,319 PPMs."	Low	Outcome measure same for all participants. Rates of physician-reported counseling for smoking cessation. Doctors enter data into electronic records, 'counseling for smoking cessation' could be subjective. There are also patient reported measures (physician administered patient surveys), patients asked pre- and post-intervention: "For smokers: has your doctor talked to you about quitting?".	Moderate	No pre-specified analysis plan but no indication that any sub-group analyses were excluded on purpose.	Serious	
Thorndike, 2007	Moderate	Adjusted for some confounders "We compared rates using weighted multiple logistic regression and controlled for patient demographics, physician specialty, and patient diagnoses." But plausible other events could have affected outcomes, no controlling for any secular trends. Causality cannot be inferred.	Moderate	Robust sampling of doctors. "The NAMCS is an ongoing annual survey of US office-based physicians conducted by the National Center for Health Statistics." "Physician response rates were 67% for 2001–2003 and 71% for 1994."	Moderate	Interventions in 1996, 1997 and 2000. It is unclear if the intervention was the 'same' for all local areas nationally, or if there were various lag times. But overall, the pre- and post- measurements are from a physician survey from 1994–1996 and 2001–2003.	Low	This is not expected in observational studies of individuals in routine care.	Moderate	No mention of missing or excluded data but assumed that the proportion of survey participants and reasons for missing data (non-response) are similar pre- and post- intervention.	Low	Outcome measure was the same pre- and post- the intervention, but are self-reported by the doctor. "Each participating physician completes a 1-page encounter form for each systematically sampled ambulatory care visit during a randomly assigned week. Physicians record information about patient demographics, patient's smoking status, expected source of payment, reasons for patient's visit, the physician's diagnoses, counseling and education provided, patient's current medications, and the duration of the visit in minutes." "(1) identification of the patient's smoking status, (2) provision of smoking counseling, and (3) report of smokers' use of nicotine replacement therapy or bupropion." Authors do not elaborate how many prescriptions of bupropion there were in the pre-period and how many of the post-period may be for depression ("Because bupropion is also used to treat depression, we excluded bupropion prescriptions prior to 1997, the year it was approved for smoking cessation.").	Moderate	No pre-specified analysis plan but no indication that any sub-group analyses were excluded on purpose.	Moderate	
Pajak, 2010	Low	Apart from the lack of random sampling of the active and control clinics, the rest of the study design is good. They adjust for confounders and have well matched the control clinics to the active ones. "There was no significant difference between active and control clinics. There were no significant differences in age and sex distribution between the groups. Patients from active clinics were slightly better educated than patients from control clinics."	Low	Does not seem like there was selection bias.	Moderate	Intervention not well described in the paper. Seems to be financially incentivising doctors to perform a primary prevention health check on patients. "However, the NFZ procedure did not include any type of structure intervention, which meant that all the decisions as to the means and extent of intervention were left to primary care physicians."	Moderate	The time the policy was implemented may have differed between local authorities nationally.	Moderate	No mention of missing or excluded data.	Moderate	For both active and control clinics: smoking status obtained from patients' medical records and receipt of smoking cessation advice obtained from patient interviews by study researchers. Paper does not mention whether the study researchers were blinded or not but "interview was carried out according to the standard questionnaire" hence rating this 'moderate'.	Moderate	No pre-specified analysis plan but no indication that any sub-group analyses were excluded on purpose.	Moderate	
Wright, 2018	Serious	Adjusted for some confounders ("client numbers, number of services by state or territory and remoteness, by TIS-funded status"). Had control group of non-funded services. But had significant issues regarding the periods of data periods they analysed (the intervention began in 2016, but they analysed data from 2014 to 2016 - only "first 6 months under the revised TIS program") - enough post-intervention data? Also, other plausible other events could have affected outcomes.	Critical	"the study had an inherent selection bias because the 2016 TIS funding was a targeted funding round to selected services based mostly on those previously funded by the TSHL program and services considered to have greater capacity to deliver tobacco control campaigns and activities. This substantially biased the results due to lack of randomisation. ... the services selected for funding may differ from those not funded by the TIS program."	Low	Population and intervention clearly outlined.	Low	This is not expected in observational studies of individuals in routine care.	Serious	"A large number of services (n = 81) was excluded from the analysis because of missing data (n = 50) or due to changing definition of a 'client' (n = 31).""65% of services were included overall."	Serious	Authors outline issues regarding 'smoking status' . "We are unsure how often smoking status is updated and this is not included as an nKPI indicator, which means that smoking status may have been recorded a long time ago and not updated." Also, they only had aggregate service-level data.	Moderate	No pre-specified analysis plan but no indication that any sub-group analyses were excluded on purpose.	Critical	
Vasankari, 2011	Serious	Some confounding factors accounted for but analysis did not account for other plausible other events which could have affected outcomes, or the underlying secular trend.	Moderate	Only one "medium-sized primary healthcare center in south-west Finland with computerized patient records was chosen" for the study.	Low	Population and intervention clearly outlined.	Low	This is not expected in observational studies of individuals in routine care.	Moderate	No mention of missing or excluded data but assumed that the proportion of participants and reasons for missing data are similar pre- and post- intervention.	Low	Outcome measure was the same pre- and post- the intervention. Electronic record of: a patient's smoking status (of patients who are seeking medical advice due to their respiratory symptoms).	Moderate	No pre-specified analysis plan but no indication that any sub-group analyses were excluded on purpose.	Serious	
McGovern, 2008	Serious	Some confounding factors accounted for (gender, age, CHD related co-morbidities, deprivation categories and practice size, clustering of patients within practices) but analysis did not account for other plausible other events which could have affected outcomes, or the underlying secular trend. Time period of analysis: "(e.g. 15 months) was determined by the nGMS contract, when it was introduced and when it had been in place for 12 months." - pre-period might be short.	Moderate	"The 310 SPICE practices used for this study were self-selected. However, the patients registered with these practices have previously been shown to be representative of the Scottish population."	Low	Population and intervention clearly outlined.	Low	This is not expected in observational studies of individuals in routine care.	Low	"We excluded from all analyses individuals with an 'exception code', which records that the person has refused follow-up or been considered unsuitable to have any of the CHD-related quality indicators measured. Individuals were also excluded from particular analyses if they had an exception code pertaining to a particular quality indicator, e.g. for aspirin treatment, if there was a recorded contraindication or allergy to this medicine." "Patients with missing data (e.g. smoking status) were excluded from the analysis of that factor." Undertook sensitivity analyses (but these were not shown): "It is possible that the exclusion of patients with exception codes who are likely to be one particular patient group, e.g. the elderly, might have impacted on our analyses; however, a further analyses, which included these patients, showed little change from existing results."	Low	Outcome measure was the same pre- and post- the intervention. Electronic record of: a patient's smoking status and provision of smoking cessation advice. GPs enter data into electronic records, 'record of smoking cessation advice' could be subjective.	Moderate	No pre-specified analysis plan but no indication that any sub-group analyses were excluded on purpose.	Serious	
Millett, 2007	Serious	Some confounding factors accounted for (gage, sex, ethnic background, deprivation group and practice level clustering) but analysis did not account for other plausible other events which could have affected outcomes, or the underlying secular trend. "Changes in achievement between the 2 study periods were assessed by conditioning 2005 achievements on 2003 achievements."	Moderate	Only one Primary Care Trust (Wandsworth Primary Care Trust) was used for this study. Contained 36 primary care practices but is not representative of the whole population of England.	Low	Population and intervention clearly outlined.	Low	This is not expected in observational studies of individuals in routine care.	Low	"The percentage of missing data in patient records was low (2.9%). We therefore restricted our analyses to records with complete information; thus, missing data had little effect on our conclusions. The 4 practices that did not participate in our study accounted for less than 6% of the registered population in the study area. The practices that did not participate were smaller (3 had fewer than 3000 patients) and were located in more deprived areas than the participating practices."	Low	Outcome measure was the same pre- and post- the intervention. Electronic record of: a patient's smoking status and provision of smoking cessation advice. GPs enter data into electronic records, 'record of smoking cessation advice' could be subjective.	Moderate	No pre-specified analysis plan but no indication that any sub-group analyses were excluded on purpose.	Serious	
Williams, 2004	Serious	This is a descriptive, repeated cross-sectional study, no controlling for confounders or secular trend. Causality cannot be inferred.	Low	General Medical Services Payments Board prescription database (for patients eligible for free medications) was used pre- and post- intervention.	Low	Population and intervention clearly outlined.	Low	This is not expected in observational studies of individuals in routine care.	Moderate	No mention of missing or excluded data but assumed that the proportion of participants and reasons for missing data are similar pre- and post- intervention.	Low	Outcome measure was the same pre- and post- the intervention. Electronic record of bupropion and NRT prescriptions dispensed is a robust, objective outcome measure.	Moderate	Only one effect estimate is reported (for the eligible patient population nationally). No pre-specified analysis plan.	Serious	
Mullins, 1999	Serious	Cross-sectional survey of smokers in the general population. Plausible other events could have affected outcomes, such as GPs seeing smoking cessation articles in medical journals and magazines, societal changes regarding anti-smoking, and RACGP's Guidelines for Preventive Activities in General Practice (released in 1996). Intervention began in 1991 - survey data is from 1990, 1992, 1994, 1996 - it is a positive that there is data from the pre-intervention 1990 year.	Moderate	Surveys were conducted by a "large market research company" using a robust cluster stratification and random sampling, interveiws were conducted at participants' households. Sampling strategy did not change over the study period. "There was no significant difference in the sample composition by sex, but there was for age. In 1992, the youngest group was under-represented and the middle group correspondingly over-represented." Response rate is not provided, neither is what proportion of the respondents were smokers (this paper only includes the responses of participants who identified themselves as being a smoker).	Low	Population and intervention clearly outlined.	Low	The intervention pack was mailed to GPs regularly from a central source. The mailing list of GPs was obtained from the Medical Board of Victoria (official register of medical practitioners).	Moderate	No mention of missing or excluded data but assumed that the proportion of participants and reasons for missing data are similar pre- and post- intervention.	Moderate	Self-reported, may be subject to recall bias - but outcome measures were the same pre- and post- the intervention. "In each year, smokers were shown a card, and asked, "Which if any of those things has your GP ever said to you?". The options were the same in each year, with some minor changes in the wording to make them easier to understand following the first survey." Post-interview, during the analysis, "each respondent was coded according to the most appropriate advice he or she had ever been given" - may be subject to human error or bias from the researchers. Final categories were "information or help to stop", "advised to stop smoking", "advised to cut down", "asked if smoked/no advice given", "none".	Moderate	Only one effect estimate is reported (the responses of all smokers per year, as proportions). No pre-specified analysis plan.	Serious	
Verbiest, 2013	Moderate	"the results cannot be extrapolated to the individual level and no confounder data were available. ... inferences regarding causality need to be made with caution, taking into account other explanations for changes in outcomes." Interrupted time series design allows accounting for secular trends and autocorrelation. Used multiple sources of data, but did not use control which (which would be unaffected by the interventions under investigation). Only one year post- one of the interventions modelled.	Low	3 data sources used. (1) Electronic health records of 350,000 patients from 84 GP clinics in the Netherlands, representative of the Netherlands population in terms of the age and gender. (2) "For prescriptions of stop-smoking medication dispensed in out-patient pharmacies, we used quarterly data of the Dutch Foundation for Pharmaceutical Statistics (SFK) in the period 2001–2012. ... representative panel of 95% of Dutch community pharmacies. Data were extrapolated to nation-wide figures." (3) National survey: Dutch Continuous Survey of Smoking Habits (DCSSH).	Low	Population and intervention clearly outlined.	Low	This is not expected in observational studies of individuals in routine care.	Moderate	No mention of missing or excluded data but assumed that the proportion of participants and reasons for missing data are similar pre- and post- intervention.	Low	Outcome measures were the same pre- and post- the interventions. Prescriptions are a robust and objective measure.	Moderate	Only one effect estimate is reported (for the whole population). No pre-specified analysis plan.	Moderate	
Fichera, 2016	Moderate	Used regression discontinuity design which can account for underlying trends. Plausible other events could have affected outcomes. Sufficient number of pre- and post-intervention time points included.	Low	Used a population cross-sectional survey (Health Survey for England (HSE)). "It is designed to be nationally representative of the English adult population with regard to age, gender, geographic area and sociodemographic circumstances. We use twelve years of data from 1997, after which income information was collected."	Low	Population and intervention clearly outlined.	Low	This is not expected in observational studies of individuals in routine care.	Moderate	"As the smoking cessation variable was not recorded in 2000, 2001 and 2002, we use a multiple imputation procedure to account for missing observations." Other mention of missing data is not present, we assume that the proportion of participants and reasons for missing data are similar pre- and post- intervention.	Low	Self-reported, may be subject to recall bias - but outcome measures were the same pre- and post- the intervention. Number of cigarettes smoked per day and whether participant was given smoking cessation advice from a medical practiitoner in the last 12 months.	Moderate	No pre-specified analysis plan but otherwise transparent reporting of various sensitivity analyses and robustness checks.	Moderate	
Van Doorn-Klomberg, 2014	Low	"evaluated the effect of practice accreditation in a design with two separate comparisons. In the first comparison we assessed improvements over time within the first cohort. In the second comparison we assessed differences between the first and second cohort by comparing the follow-up measurement of the first cohort with the start measurement of the second cohort." "We used a matched design to select an equally large sample of 69 practices ... Matching was based on availability of data, practice location (degree of urbanization), visitation date, practice type, and practice size." Causation cannot be inferred but the study is comparable to a well-performed randomized trial with regard to this domain.	Moderate	"Practices have been invited to participate voluntarily in the Dutch practice accreditation program since 2005." Not randomised. "In our models, we accounted for practice type, size and location. However, there may have been an underrepresentation of practices with a less than average interest in quality improvement, especially in the first cohort, since participation in the program was voluntary."	Low	Population and intervention clearly outlined.	Low	This is not expected in observational studies of individuals in routine care.	Moderate	Not much mention of missing data, but in their outline of the selection of the second cohort, 362 out of 802 practices which started the accreditation program between 2009-2011 had "no data on diabetes, COPD and/or CVD". Quite high proportion (45%).	Low	Proportion of patients which known smoking status, proportion of patients that smoking who were given stop smoking advice. Submitted via electronic health record database. Outcome measure was the same at start (pre-) and post- the intervention. Doctors enter data into electronic records, 'record of stop smoking advice given' could be subjective.	Moderate	No pre-specified analysis plan but no indication that any sub-group analyses were excluded on purpose.	Moderate	
Bailey, 2016	Moderate	Used control group. Adjusted for confounders. "Because the insured and uninsured groups differed in multiple characteristics, a propensity score matching approach was used to balance potential confounders between groups and reduce bias. We used logistic regression models to generate propensity scores based on baseline and pre-baseline characteristics including demographics, insurance history, utilization, smoking history (e.g., years smoked at baseline, number of pre-baseline smoking assessments), comorbidities, and health center characteristics." Robust analysis: "Between-group differences were tested using generalized estimating equations (GEE) to account for clustering among propensity score-matched pairs. ... For our GEE models, we assumed a compound symmetry correlation structure and applied a robust sandwich variance estimator to account for possible misspecification." In Appendix, the authors included a table comparing "characteristics of the propensity score matched sample to smokers excluded for having no follow-up smoking assessment (N = 7524)." Because we used EHR data, we only had follow-up smoking status for those patients who returned to the clinic, and therefore cannot determine the quit status of non-returning patients. Non-returning patients differed from the study sample on a number of baseline demographics; the results of the study may not generalize to this group. Although we used propensity score matching to balance a range of baseline factors, there could be residual confounding due to unmeasured variables that could account, in part, for the observed differences."	Low	Used comprehensive OCHIN Oregon community health centres electronic healthcare record dataset. "We obtained Medicaid enrollment data from the state of Oregon and linked it to EHR data using a unique Medicaid patient identifier available in both data sets." "After exclusions, we had a cohort of 5935 current smokers who gained Medicaid. To identify a matched control group of smokers who did not gain Medicaid, we considered patients who were continuously uninsured throughout the 24-month follow-up period and met the current smoker criteria (n = 9371 after excluding 6344 smokers with no follow-up)."	Low	Population and intervention clearly outlined.	Low	This is not expected in observational studies of individuals in routine care.	Low	Proportion of missing data in the final intervention and control groups are similar.	Low	Outcome measure was the same for the intervention and control group. "Our primary outcome was 'quit' smoking status after the baseline assessment, coded as a binary yes/no variable. A person was identified as 'quit' if baseline smoking status was 'current every day' or 'some day' and status changed to 'former smoker' at a subsequent visit. We also assessed prevalence of having a smoking cessation medication ordered (yes/no), and analyzed quit smoking status stratified by whether medication was ordered. Medications included bupropion, varenicline, and all nicotine replacement products." "We did not assess if bupropion was prescribed for smoking cessation or depression; however, we used propensity scores to match patients on psychiatric diagnoses, including depression. It should be noted that we were only able to assess whether medications were prescribed, not whether the patients filled the prescriptions and took the medications. Finally, as mentioned previously, we were not able to reliably capture counseling services or referrals in the EHR during the study period."	Low	Study was "registered as an observational study at clinicaltrials.gov". No indication that any sub-group analyses were excluded on purpose.	Moderate	
Li, 2020	Serious	Used control groups (LCS-ineligible smoker patients) and a pre-, post design, and adjusted for some confounding factors (age, sex, race/ethnicity). But other plausible other events could have affected outcomes. "One potential contributor is the rereleased USPSTF recommendation in 2015 for clinicians to offer cessation support to smokers. ... there were no efforts in the healthcare system specifically targeting tobacco treatment during 2010–2017."	Moderate	Only one 'healthcare system' was used for this study ("a large healthcare system in northern California"). Is not representative of the whole population of the US. "the data come from a single healthcare organization whose primary care base reflects a relatively well-insured patient population. However, the organization covers a substantial geographic area that includes low-income, rural, non-English speaking, and minority patients reflecting the population of California."	Low	Population and intervention clearly outlined.	Low	This is not expected in observational studies of individuals in routine care.	Moderate	"Excluding those visits (n=7,184) in which eligibility for LDCT-LCS could not be determined owing to lack of sufficient information on smoking history, 63.8% of 19,862 (n=12,678) current smokers were included in the analysis." Missing data for race/ethnicity shown, similar proportion in pre- and post- samples. No other mention of missing or excluded data.	Low	Outcome measure was the same pre- and post- the intervention. Electronic record of: Smoking cessation counselling (formal and informal), referral to smoking cessation program, prescription for smoking cessation pharmacotherapy. "An internal EHR data analyst reviewed a random sample of physician's notes and confirmed that the quality metric of tobacco-cessation counseling is a reliable indicator for in-visit smoking cessation counseling. Keyword searches included but were not limited to, smoking cessation and tobacco counseling in the procedure description." Limitation: "EHR structured fields may not have captured all informal counseling sessions and referrals"	Moderate	No pre-specified analysis plan but no indication that any sub-group analyses were excluded on purpose.	Serious	
Fortmann, 2020	Moderate	Plausible other events could have affected outcomes but authors used time series design which can account for underlying long-term and/or seasonal trends. Sufficient number of time period pre- and post intervention. Also investigated various confounders.	Moderate	Electronic health records of 15 community health centres from 9 states in the US were used.	Low	Population and intervention clearly outlined.	Low	This is not expected in observational studies of individuals in routine care.	Low	Transparent about the amount of missing data.	Low	Outcome measure was the same pre- and post- the intervention. Electronic record of a patient's smoking status.	Moderate	Paper includes results of time series analysis but only graphically displays 2006 and 2013 time point data. Per patient characteristic, they display findings from their final multivariable model. No pre-specified analysis plan.	Moderate	
Miraldo, 2018	Low	"We exploit the expansion of insurance coverage that was rolled out within Massachusetts in 2006 but not in ONES. The difference-in-differences (DD) approach reduces bias in results due to common trends by comparing differences in outcomes between states without the policy (control states, ONES) and states with the policy (treated, Massachusetts) before and after the introduction of the policy." "The ONES are a natural comparison group as they were unexposed to the reform and have similarities to Massachusetts in demographics, geography, the economy, culture and politics." Triple differences model: "we further compare a treatment group and a control group within the treatment state with a treatment group and control group within control states. Adults above 300% of the FPL serve as a comparison group within states as they did not receive publicly subsidised health insurance." Study looks at outcomes four years post-intervention. Considered various confounding factors like socioeconomic status.	Moderate	Used state-based survey (Behavioural Risk Factor Surveillance System (BRFSS)) which was "shown to be a reliable data source for estimation of the prevalence of health-related factors and produces results consistent with other national surveys". But authors outline low response rates for some years and states. "Massachusetts had the lowest response rate from 2001 to 2007 and for 2010, ranging from 34.6% to 47.7%. In 2008 and 2009, Connecticut had the lowest response rate at 39.8% and 44.23% respectively. The highest response rate was for Vermont in 2001 and from 2003 to 2010, ranging from 52.1% to 60.5%. In 2002 Maine had the highest response rate at 59.4%." "landlines were used for sampling in the BRFSS means that the study cohort may not be representative of the population ... however, the sampling weights used in our analysis should account for this source of possible bias."	Low	Population and intervention clearly outlined.	Low	This is not expected in observational studies of individuals in routine care.	Moderate	No mention of missing or excluded data but assumed that the proportion of participants and reasons for missing data are similar between the groups compared.	Low	The outcome measure was self reported quit attempts: "Current smokers that tried to quit smoking in the past year". "data is self-reported so responses may be prone to recall bias" but "as the recall problem affects both the treatment and control states, our DD and DDD designs should not be affected by such bias". "Sample sizes differed for specific outcome variables, as some questions were not asked every year."	Moderate	No pre-specified analysis plan but results shown for various sub-group analyses. Also outline sensitivity analyses performed.	Moderate	
Donner-Banzhoff, 1996	Serious	Considered confounders (sociodemographic data, medication, diagnoses, risk factor concepts). Compare two interventions, no pre- or post- data. Also there may be other country-wide differences present.	Moderate	"15 family practitioners' surgeries in Germany and the UK that were matched for rural-urban location were included in a cross-sectional survey". Questionnaire plus interview of patients. Sampling/selection of clinics not outlined in detail. "German FPs are usually more reluctant to take part in research. We chose from practices involved in previous research by matching them according to urban-intermediate-rural location. The demograhic features of the samples were very similar to those in truly representative surveys from both these countries."	Serious	Intervention not clearly defined, nor the study time period under investigation.	Low	This is not expected in observational studies of individuals in routine care.	Moderate	Missing data outlined for smoking status question, but not clear whether the proportion of missing data was similar between the comparison groups.	Moderate	Questionnaire plus interview of patients. "Study interviews and examinations were performed by one researcher in both countries." "An intervention was assumed if the question "Has your family doctor ever talked to you about your smoking?" was answered by "yes" or if questions about possible interventions by doctor or nursing staff were answered in the affirmative." "The questionnaire was developed simultanously in German and English. It was then translated from English into German to correct linguistic ambiguities." Recall bias, but likely same rate in the two groups under comparison.	Moderate	No pre-specified analysis plan but no indication that any sub-group analyses were excluded on purpose. Results shown for the cohort of smoker patients as a whole.	Serious	

Frijling, 2003	Serious	Use of control practices is good, but the trial and control practices differed at baseline so the effect of the intervention is confounded by this. Recruitment of the 800 practices took place "between January 1998 and August 1998". "The intervention period lasted from September 1998 to September 2000." "We used postal questionnaires to gather both baseline information (October 1998) and post-intervention information (September 2000)." Slightly unclear about the baseline measure time period.	Moderate	800 practices (17% of all Dutch practices) participated in the intervention. Of these, this study compared 420 (randomly selected from the 800) practices with 600 (selected from 4000 practices which did not participate in the intervention). "The sampling was stratified by type of practice (single-handed versus group), and practice location (>50 000 inhabitants or not)." The original 800 practices which undertook the intervention were not randomly selected - "the trial groups differed at baseline with the intervention group showing relatively greater task delegation and a higher quality of care structure that may have facilitated greater change in turn."	Low	Population and intervention clearly outlined.	Low	This is not expected in observational studies of individuals in routine care.	Moderate	Missing data - practices not wanting to participate/not returning questionnaires is not discussed. Numbers are shows in a flow diagram - proportions between intervention group and control are different.	Moderate	"postal questionnaires to gather both baseline information (October 1998) and post-intervention information (September 2000). Reminders were sent to non-responders after 2 and 4 weeks. Those practices that responded at baseline but not after the post-intervention reminders were sent shortened versions of the questionnaire. The information was provided by one GP per practice and the same GP for both measurement points." "the use of self-administered questionnaires may have biased the results and increased the observed effects on the process of care in particular."	Moderate	No pre-specified analysis plan. Results shown for intervention and control cohorts as a whole.	Serious	
Bennett, 2008	Moderate	No control group, pre-, post- design. Plausible other events could have affected outcomes, but they did adjust for confounders. "Unlike a randomized controlled trial, Heartwatch has captured 'real-life' health care by a representative group of GP nationally. This makes it more generalizable to a wider population than a randomized controlled trial."	Moderate	"In total, 470 general practitioners (GPs) – 20% of all Irish GPs – were selected to participate in the programme. Demographic and clinical data were electronically transferred from GP practices to an Independent National Data Centre, where the data is centrally stored." State that the study involved a "representative group of GP nationally". Not stated how the 470 GPs were chosen.	Low	Population and intervention clearly outlined.	Low	This is not expected in observational studies of individuals in routine care.	Moderate	Attrition numbers outlined. "Attrition in the number of patients eligible for analysis was observed, because of the GP visits not being scheduled at the recommended frequency (four times per year), but this is unlikely to affect the overall conclusions from the programme. ... To be eligible for analysis, patients were required to have attended for a baseline, 1-year and 2-year visit. The attrition in numbers at 2 years is unlikely to have affected the results as the demographics at baseline were similar in the 1-year and 2-year cohorts." One-year follow-up cohort: "The total number was 7099, with the majority (84.4%) having four or five visits over the year." Two-year follow-up cohort: "The total number was 4011 and most (60.5%) had at least eight or nine visits over 2 years."	Low	Outcome measure was the same at baseline and in the follow-up consultations. Electronic record of a patient's smoking status. "the percentage smoking prevalence was calculated based on an individual having at least one of the following recorded: smoker of one or more cigarettes per day, cigar or pipe smoker."	Moderate	No pre-specified analysis plan but no indication that any sub-group analyses were excluded on purpose. Results shown for the cohort of patients as a whole.	Moderate	
Fitzpatrick, 2011	Moderate	No control group, pre-, post- design. Plausible other events could have affected outcomes, but they did adjust for confounders. "Unlike a randomized controlled trial, Heartwatch has captured 'real-life' health care by a representative group of GP nationally. This makes it more generalizable to a wider population than a randomized controlled trial."	Moderate	"In total, 470 general practitioners (GPs) – 20% of all Irish GPs – were selected to participate in the programme. Demographic and clinical data were electronically transferred from GP practices to an Independent National Data Centre, where the data is centrally stored." State that the study involved a "representative group of GP nationally". Not stated how the 470 GPs were chosen.	Low	Population and intervention clearly outlined.	Low	This is not expected in observational studies of individuals in routine care.	Serious	Attrition numbers outlined. "For the 1-year cohort, patients with no valid data at 1 year (4049) were excluded to give 8309 patients. The 2, 3 and 3.5-year cohorts consisted of 5431, 3470 and 2078 patients, respectively." High attrition rate not commented on in the paper.	Low	Outcome measure was the same at baseline and in the follow-up consultations. Electronic record of a patient's smoking status. "the percentage smoking prevalence was calculated based on an individual having at least one of the following recorded: smoker of one or more cigarettes per day, cigar or pipe smoker."	Moderate	No pre-specified analysis plan but no indication that any sub-group analyses were excluded on purpose. Results shown for the cohort of patients as a whole.	Serious	
Parnes, 2002	Moderate	Adjusted for confounders (sex, age, ethnicity, and race, duration of visit, whether the patient had been seen before in the practice, and whether the patient had at least 1 of the chronic conditions listed on the NAMCS form (hypertension, depression, obesity, or hypercholesterolemia)). 3 intervention groups were compared at the same study period.	Moderate	The study sample consisted of the "7 primary care practices in the Colorado Research Network (CaReNet)", it broadly reflect the demogrpahics of Colorado. "A modified version of the 1994 National Ambulatory Medical Care Survey (NAMCS) was administered in each CaReNet practice. The NAMCS instrument is a physician survey." "CaReNet providers completed NAMCS forms on 2773 patient encounters of 2800 eligible visits (99% completion rate)."	Low	Population and intervention clearly outlined.	Low	This is not expected in observational studies of individuals in routine care.	Low	Mentions that "CaReNet providers completed NAMCS forms on 2773 patient encounters of 2800 eligible visits (99% completion rate)." And that 90% of pateints had a known smoking status (yes, no unknown). But no further comment about missing data. We assume that the proportion of participants and reasons for missing data are similar between the groups compared.	Low	Physician survey, outcome measure is self-reported by the doctor, but outcome measure was the same across the 3 interventions investigated. "we examined the impact of patient insurance on 2 primary outcomes: (1) patient smoking cessation counseling. Each provider coded smoking status as "Yes," "No," or "Unknown." Only patients with a known smoking status (90% of sample) were included in the present analysis. For those patients coded as smokers, we determined whether providers checked the "Smoking Cessation" box."	Moderate	No pre-specified analysis plan but no indication that any sub-group analyses were excluded on purpose.	Moderate	
Shi, 2017	Moderate	Adjusted for various confounders (patient characteristics, provider characteristics, financial characteristics, and practice characteristics). Used control group (health centres without PCMH recognition). But is a cross-sectional study, "it is possible that high-performing health centres and those with greater resources already had PCMH features in place. These health centres may have been more likely to seek and gain PCMH recognition."	Low	Used comprehensive datasets containing data nationally from all health centres. "Health Resources and Services Administration [HRSA] 2012 Uniform Data System (UDS) and PCMH tracking data from HRSA's Patient-Centered Medical/Health Home Initiative". "1,087 health centres were included in the analysis, with a total of 539 health centres (46%) having achieved PCMH recognition." "The current study examined all HRSA-funded HCs providing services to more than 21 million patients." "nationally representative"	Low	Population and intervention clearly outlined.	Low	This is not expected in observational studies of individuals in routine care.	Moderate	No mention of missing or excluded data but assumed that the proportion of participants and reasons for missing data are similar between the groups compared.	Low	Clinical performance measures from datasets. Same outcomes for intervention and control groups. "percent of adults (18 years or older) assessed for tobacco use", "percent of adults (18 years or older) who were known tobacco users that received tobacco cessation counseling and/or pharmacologic intervention."	Moderate	No pre-specified analysis plan but no indication that any sub-group analyses were excluded on purpose. Adjusted and unadjusted analyses shown.	Moderate	
Bailey, 2020	Moderate	Used control group. Propensity Score matching was used to balance confounders between the intervention and control group (sex, age, race/ethnicity, household income as percent of FPL, location of patient's primary clinic (urban/rural), insurance status at the majority of visits, cessation medication order (y/n), and medical comorbidities associated with tobacco use). "After exclusions, 41% of remaining patients (n = 295 220) had a tobacco assessment in the 24-month pre-expansion period and of those 32% (n = 94 045) were current tobacco users in their final pre-expansion assessment. We propensity score matched these patients to tobacco users with the same inclusion criteria from non-expansion states." "We only had follow-up tobacco status for patients who had a return clinic visit, and therefore, we cannot determine the quit status of non-returning patients. ... Although we used Propensity Score matching to balance a range of baseline factors, there could be residual confoundingndue to unmeasured variables accounting for the observed differences."	Low	"We included primary care community health centres "live" on their EHR as of January 1, 2013 (n = 219 CHCs) in 10 states that had expanded Medicaid eligibility to ≤138% of the federal poverty level for all adults including those without dependent children as of January 1, 2014 (California, Hawaii, Maryland, Minnesota, New Mexico, Ohio, Oregon, Rhode Island, Washington, and Wisconsin) and 108 CHCs in six nonexpansion states (Florida, Kansas, Missouri, North Carolina, Texas, and Montana)."	Low	Population and intervention clearly outlined.	Low	This is not expected in observational studies of individuals in routine care.	Low	"We did observe a small to moderate amount of missing data on race/ethnicity and FPL, though enough to include these patients by creating missing data categories in our modeling."	Low	Outcome measure was the same for the intervention and control group. "The EHR presents a discrete data field for tobacco use status at each primary care encounter, which can be confirmed, updated, or not reviewed. If confirmed or updated, the date is saved. Our primary outcome was tobacco cessation ("quit") during the post-period, coded as a binary yes/no variable. Using methods similar to prior EHR-based studies, a person was identified as "quit" if the last recorded tobacco-use status during the pre-period indicated that the patient was a current user, and if there was at least one subsequent measurement documented in the postperiod that indicated the patient's status was a "nonuser" (eg, former user, not a current user). We extracted the following tobacco cessation medications from EHR medication orders: bupropion, varenicline, and all nicotine replacement products." "We were unable to assess if bupropion was prescribed for tobacco cessation or depression; however, all patients in the study sample were current tobacco users and our models controlled for depressive disorders."	Moderate	No pre-specified analysis plan but no indication that any sub-group analyses were excluded on purpose. Conducted sensitivity analyses.	Moderate	
Langley, 2012	Moderate	Plausible other events could have affected outcomes, it was not possible to separate out the effects of other events that occurred at the time of the intervention(s). Authors used a time series design and presented analyses including and excluding the effect of seasonality.	Low	Electronic health records GP clinics in the UK, representative of the UK population.	Low	Population and intervention clearly outlined.	Low	Intervention(s) run over a defined time period.	Moderate	No mention of missing or excluded data but assumed that the proportion of participants and reasons for missing data are similar pre- and post- intervention.	Low	Outcome measure was the same pre- and post- the intervention. Electronic record of NRT prescriptions issued is a robust, objective outcome measure.	Moderate	Only one effect estimate is reported (for the whole patient population). Authors present both the seasonally adjusted and unadjusted analyses. No pre-specified analysis plan.	Moderate	
Sutton, 2010	Moderate	Pre-, post- design. Compared risk factors in patient groups incentivised by the intervention versus risk factors in patient groups not incentivised by the intervention. Used dynamic panel probit models.	Moderate	"The practices that participate in SPICE-PC are volunteers and would be expected to be more interested in record-keeping. A range of practice characteristics for the 315 practices in the sample were compared with the 721 other Scottish practices. Participation in SPICE-PC was less likely in the most deprived areas and showed some geographical concentration. Compared with non-participants, participating practices had more patients in total (but fewer patients per GP), were more likely to also participate in other voluntary initiatives and achieved 1% more points on average on the 2005/6 QOF. This suggests some caution in extrapolating the results to all Scottish practices." "The analysis is restricted to patients aged 45 years and over. Although this is only 37.1% of the full data set, it represents 92.2% of the targeted disease groups and is the age cutoff for the requirement to record blood pressure for all patients under the QOF. We make this restriction to focus on the population for whom risk factor recording is most important."	Low	Population and intervention clearly outlined.	Low	This is not expected in observational studies of individuals in routine care.	Moderate	No mention of missing or excluded data but assumed that the proportion of participants and reasons for missing data are similar pre- and post- intervention.	Low	Outcome measure was the same pre- and post- the intervention. Electronic record of: a patient's smoking status.	Moderate	No pre-specified analysis plan but no indication that any sub-group analyses were excluded on purpose. The results of different models are displayed.	Moderate	
Forster, 2016	Low	"Control participants were selected who were eligible for NHS Health Checks but did not receive an NHS Health Check. Control participants might have been invited for a Health Check, but did not attend, or might not have been invited." Controlled for age, gender, general practice, socioeconomic status.	Low	Electronic health records of 334 GP clinics in the UK, representative of the UK population. "...91 618 participants who had a health check between 1 April 2010 and 31 March 2013, who had at least 12 months record, were never treated with antihypertensive drugs or statins, and were not diagnosed with diabetes, stroke or CHD before the check. For these 91 618 participants, eligible control participants were identified for 75 123 (82%) and these participants, and their matched controls, were included in analyses."	Low	Population and intervention clearly outlined.	Low	This is not expected in observational studies of individuals in routine care.	Low	No mention of missing data except at initial inclusion/exclusion stage "We also excluded patients (4859) who did not have a full year of up to standard record before the date of the check and one patient whose check was after 31 March 2013." Same criteria applied to intervention and control participants, proportion of exclusions are similar.	Low	Outcome measure was the same pre- and post- the intervention and the same for the intervention and matched control participants. Electronic record of: a patient's smoking status, proportion of patients recorded as current smokers.	Low	Study protocol included and no indication that any sub-group analyses were excluded.	Low	
Li, 2018	Serious	Various confounding factors were examined (age, sex, race/ethnicity, language preference, smoking status, smoking history, severity of major comorbidities (Charlson Comorbidity Index (CCI)), and visiting his/her own PCP at the visit in which the first LDCT-LCS order was made, frequency of office visits during the calendar year, and calendar year were controlled for. ... Provider-level factors include sex, physician (vs. non-physician clinician), and where the PCP received their medical degree (in the US or outside of US). Plausible other events could have affected outcomes, do not account for underlying trend.	Moderate	Only one 'healthcare system' was used for this study ("a large healthcare system in northern California"). Is not representative of the whole population of the US. "using data from a single healthcare organization with a generally highly-insured patient population may limit the generalizability of our findings."	Low	Population and intervention clearly outlined.	Low	This is not expected in observational studies of individuals in routine care.	Low	Not much mention of missing data. "In the first part of the study, a patient-year was the unit of analysis. Because the guidelines recommend annual screening, we examine each year separately, so a patient could appear in the denominator (and numerator) in multiple years. In each year, we examine a patient's smoking history information as represented in his or her EHR that year, without imputation of missing values using documented smoking history in the earlier or later years."	Low	Outcome measure was the same pre- and post- the intervention. Electronic record of: a patient's smoking status.	Moderate	No pre-specified analysis plan but no indication that any sub-group analyses were excluded on purpose.	Serious	
Marino, 2016	Low	Used control group. Adjusted for confounders (age, sex, race/ethnicity, household income, and baseline health status prior to each patient's selection date). Robust analysis: "intent-to-treat (ITT) analyses were conducted for each outcome, comparing preventive service receipt in the 36 month post-period among those randomly selected to apply versus not selected using generalized estimating equation models with a logit link and robust sandwich variance estimator to account for the clustering of patients within community health centres." "The randomization component of the Oregon Experiment enabled examination of both the effect of being selected to apply for Medicaid coverage on utilization of CHC services, and the isolated effect of actually gaining Medicaid coverage."	Low	"From a "reservation list" of >100,000 entries, approximately 30,000 people were randomly selected to apply, and approximately 10,000 gained coverage." In the study, the authors attempted to identify the people who gained coverage and patients who were on the reservation list but were not selected to gain coverage. "The final study population consisted of 10,643 patients: 4,049 selected to apply for coverage and 6,594 not selected." Robust selection of intervention and control groups outlined.	Low	Population and intervention clearly outlined.	Low	This is not expected in observational studies of individuals in routine care. Authors note that: "individuals randomly selected to apply for insurance did not always follow through, and thus remained uninsured." Both 'selected to apply for coverage' and 'gained coverage' results outlined.	Low	Intervention and control patients appear well matched. We assume that the proportion of participants and reasons for missing data are similar between the groups.	Low	"to limit bias inherent in self-reported measures, this study utilized electronic health record (EHR) data from 49 CHCs. It assessed participants' receipt of preventive services, as documented in the EHR, in the 36 months after the Oregon Experiment." Outcome measure was the same for the intervention and control group. Electronic record of: a patient's smoking status ("screening for smoking").	Low	Study was "registered as an observational study at clinicaltrials.gov". Both 'selected to apply for coverage' and 'gained coverage' results outlined. Adjusted and unadjusted analyses shown.	Low	
Simpson, 2006	Serious	Adequate study design: "March 31, 2004 (1 year before introduction of the new contract in April 2004, designated as the "precontract" period in this article; total population at risk 1 806 266 patients) and March 31, 2005 (1 year after introduction of the new contract in April 2004; designated as the "postcontract" period; 1 775 397 patients). All registered patients with a recording of stroke before the 2 time points were included in the analyses." Accounted for some confounding factors (sex, age, deprivation, practice, and comorbidity categories). "The nonexperimental design of the study means that we cannot directly attribute the changes observed to the new contract." Doesn't account for underlying trend.	Moderate	"Anonymous retrospective data from all 310 of the 850 Scottish practices that use the General Practice Administrative Software System and that participate in SPICE were obtained in November 2005. These 310 practices were self-selected; however, they have been shown to be representative of all Scottish practices."	Low	Population and intervention clearly outlined.	Low	This is not expected in observational studies of individuals in routine care.	Low	"Patients with missing data (eg, smoking status) were excluded from the analysis of that factor." "We excluded from the analysis anyone with a record of "exception codes," which indicate that the person has refused or been considered unsuitable to have any quality indicators measured. Patients were also excluded from particular analyses when an exception code pertaining to a quality indicator existed, eg, for aspirin treatment, if there was a recorded contraindication or allergy to this medicine." "By March 31, 2004 (precontract), 21 901 patients had a computer record of any stroke or TIA (1.2% of everyone registered with the practices; 95% CI, 1.1% to 1.2%). Forty-six patients had a computer record of an exception code. By March 31, 2005 (postcontract), the corresponding number of patients was 32 401 (1.8%; 95% CI, 1.8% to 1.8%), of whom 2565 had a computer record of an exception code."	Low	Outcome measure was the same pre- and post- the intervention. Electronic record of: a patient's smoking status and provision of smoking cessation advice. GPs enter data into electronic records, 'record of smoking cessation advice' could be subjective.	Moderate	No pre-specified analysis plan but no indication that any sub-group analyses were excluded on purpose.	Serious	
