# Supplementary material for: Implementation strategies to increase smoking cessation treatment provision in primary care: a systematic review of observational studies
Source: BMC Prim Care. 2023 Jan 25;24:32. doi: 10.1186/s12875-023-01981-2 (PMC9875430; doi:10.1186/s12875-023-01981-2)
Supplement: Supplementary file 8 — Additional file 8: Appendix 8. Supplementary table containing long-form quantitative outcome measures for RQ2 effectiveness. [file 12875_2023_1981_MOESM8_ESM.docx]

Appendix. Supplementary table containing long-form quantitative outcome measures for RQ2 effectiveness

| First author, year | Location | Implementation strategy category | Study design | Outcome measures |
| --- | --- | --- | --- | --- |
| Domain 5. Train and educate stakeholders | | | | |
| Mullins, 1999 (70) | Victoria, Australia | 40. Distribute educational materials | Repeated cross-sectional study.  Analytical. | **Recall of asking about smoking status** (GP "asked if smoked/no advice given"): 1990: 22.4%, 95% CI: 19.2 to 25.7. 1992: 21.3%, 95% CI: 18.0 to 24.6. 1994: 15.6%, 95% CI: 12.7 to 18.5. 1996: 19.2%, 95% CI: 15.9 to 22.4.  **Recall of receiving cessation advice** (GP "advised to stop smoking"): 1990: 34.8%, 95% CI: 31.0 to 38.5. 1992: 37.2%, 95% CI: 33.4 to 41.1. 1994: 37.4%, 95% CI: 33.6 to 41.3. 1996: 35.2%, 95% CI: 31.2 to 39.1.  **Recall of GP assisting to quit** (GP gave "information or help to stop"): 1990: 10.7%, 95% CI: 8.3 to 13.2. 1992: 13.1%, 95% CI: 10.4 to 15.8. 1994: 17.2%, 95% CI: 14.2 to 20.2. 1996: 20.6%, 95% CI: 17.3 to 23.9. Statistically significant increase "over time": X^2=17.58, p<0.001.  "In 1996, 9% of smokers said their doctor had advised them to contact Quit (this response was subsumed into the category “information or help to stop”).  **Recall of advise to cut down** (GP "advised to cut down"): 1990: 11.4%, 95% CI: 8.9 to 13.9. 1992: 10.2%, 95% CI: 7.8 to 12.7. 1994: 11.0%, 95% CI: 8.5 to 13.5. 1996: 9.2%, 95% CI: 6.8 to 11.6. |
| Vasankari, 2011 (74) | Finland | 42. Conduct educational meetings | Repeated cross-sectional study.  Analytical. | **Record of smoking status:** All patients with respiratory symptoms: 1997: 16.6% of all patients "had written information on smoking habits". (n = 178/1,072) 2002: 53.2%. (n = 875/1,645) Statistically significant increase: p<0.001.  In patients with COPD: 1997: 45.0% of all patients "had written information on smoking habits". (n = 45/100) 2002: 84.3%. (n = 182/216) Statistically significant increase: p<0.001. |
| Domain 7. Engage consumers | | | | |
| Szatkowski, 2011 (33) | England | 54. Prepare patients/consumers to be active participants | Repeated cross-sectional study.  Interrupted time series analysis (no control). | **Prescription for NRT:** 9 months before: 4.0% change, 95% CI: -1.3 to 9.3, p=0.135 6 months before: 6.2% change, 95% CI: 1.4 to 11.0, p=0.012 3 months before: 10.4% change, 95% CI: 5.0 to 15.7, p<0.001 2 months before: 13.6% change, 95% CI: 8.1 to 19.1, p<0.001 1 month before: 17.5% change, 95% CI: 11.1 to 24.0, p<0.001 1 month after: -1.1% change, 95% CI: -32.2 to 30.0, p=0.945 2 months after: -6.9% change, 95% CI: -0.3 to -13.4, p=0.040 3 months after: -9.0% change, 95% CI: -3.9 to -14.2, p=0.001 6 months after: -6.7% change, 95% CI: -2.1 to -11.2, p=0.004 9 months after: -5.5% change, 95% CI: -2.3 to -8.7, p=0.001 Permanent change: -1.7% change, 95% CI: -4.4 to 1.0, p=0.229  **Prescription for bupropion:** 9 months before: 5.2% change, 95% CI: -1.8 to 12.3, p=0.147 6 months before: 7.1% change, 95% CI: -0.4 to 14.5, p=0.062 3 months before: 13.2% change, 95% CI: 4.3 to 22.2, p=0.004 2 months before: 18.9% change, 95% CI: 9.2 to 28.6, p<0.001 1 month before: 44.7% change, 95% CI: 20.4 to 69.0, p<0.001 1 month after: -6.8% change, 95% CI: -40.1 to 26.6, p=0.691 2 months after: -25.3% change, 95% CI: -4.9 to -45.7, p=0.015 3 months after: -21.1% change, 95% CI: -2.1 to -40.1, p=0.029 6 months after: -19.7% change, 95% CI: -5.5 to -34.0, p=0.007 9 months after: -13.7% change, 95% CI: -4.6 to -22.8, p=0.003 Permanent change: -3.5% change, 95% CI: -8.8 to 1.9, p=0.206  **Prescription for all medications:** 9 months before: 6.4% change, 95% CI: 0.7 to 12.1, p=0.027 6 months before: 11.1% change, 95% CI: 5.5 to 16.7, p<0.001 3 months before: 9.9% change, 95% CI: 5.2 to 14.6, p<0.001 2 months before: 14.7% change, 95% CI: 10.4 to 19.1, p<0.001 1 month before: 22.3% change, 95% CI: 17.9 to 26.8, p<0.001 1 month after: 7.7% change, 95% CI: -13.0 to 28.4, p=0.468 2 months after: -5.3% change, 95% CI: -17.2 to 6.7, p=0.387 3 months after: -10.0% change, 95% CI: -0.2 to -19.9, p=0.046 6 months after: -7.4% change, 95% CI: -16.3 to 1.5, p=0.101 9 months after: -6.4% change, 95% CI: -1.1 to -11.7, p=0.019 Permanent change: -2.2% change, 95% CI: -5.6 to 1.2, p=0.209 |
| Langley, 2012 (46) | England (and Wales) | 56. Use mass media | Repeated cross-sectional study.  Interrupted time series analysis (no control). | **Prescription for NRT:**  Intervention: Tobacco control TVRs: In both the seasonally adjusted and unadjusted models, tobacco control campaign advertising had no statistically significant effect on NRT prescribing (January 2002 to June 2009) in the same month. Unadjusted model: 0.034 Orthogonalised Impulse Response Function (OIRF); 95% CI: -0.008 to 0.077, p=0.121. Seasonally adjusted model: 0.012 OIRF; 95% CI: -0.007 to 0.031, p=0.220.  Intervention: Pharmaceutical company TVRs: In both the seasonally adjusted and unadjusted models, pharmaceutical company advertising had no statistically significant effect on NRT prescribing (January 2005 to June 2009) in the same month. Unadjusted model: 0.028 Orthogonalised Impulse Response Function (OIRF); 95% CI: -0.023 to 0.080, p=0.285. Seasonally adjusted model: 0.020 OIRF; 95% CI: -0.004 to 0.044, p=0.121. |
| Domain 8. Utilize financial strategies | | | | |
| Alageel, 2019 (31) | England | 57. Fund and contract for the clinical innovation | Cohort study.  Interrupted time series analysis (with control). | **Record of referral to smoking cessation advisor or stop smoking clinic:**  Health check participants: 19,818 (90%); Controls: 48,900 (61%). Adjusted HR: 3.13; 95% CI: 3.07 to 3.20, p<0.001.  **Prescription for NRT:** Health check participants: 3,956 (18%); Controls: 8,630 (11%). Adjusted HR: 1.63; 95% CI: 1.57 to 1.69, p<0.001. **Record of all smoking cessation interventions:** Health check participants: 19,927 (91%); Controls: 42,282 (61%). Adjusted HR: 3.20; 95% CI: 3.13 to 3.27, p<0.001.   **Smoking prevalence:** 'Current smoking' OR:  Mean difference between cases and controls: 0.70, 95% CI: 0.69 to 0.71, p<0.001. Mean change per year for cases and controls: 0.97, 95% CI: 0.96 to 0.97, p<0.001. 1st year following the health check: 0.97, 95% CI: 0.96 to 0.98, p<0.001. 2nd year following the health check: 0.93, 95% CI: 0.92 to 0.94, p<0.001. 3rd year following the health check: 0.91, 95% CI: 0.89 to 0.93, p<0.001. 4th year following the health check: 0.91, 95% CI: 0.89 to 0.93, p<0.001. 5th year following the health check: 0.92, 95% CI: 0.90 to 0.94, p<0.001. 6th year following the health check: 0.90, 95% CI: 0.87 to 0.94, p<0.001. |
| Bennett, 2008 (65) | Ireland | 57. Fund and contract for the clinical innovation | Cohort study. | **Smoking prevalence:  1-year follow up cohort:** n (with data at both visits) = 7,097. Baseline: 14.8% smoking. 1-year: 12.0% smoking. Statistically significant difference between 1-year and baseline: -2.8%, p<0.0001.  **2-year follow up cohort:** n (with data at all visits) = 4,011. Baseline: 13.7% smoking. 1-year: 11.3% smoking. 2-year: 10.1% smoking. Statistically significant difference between 2-year and baseline: -3.6%, p<0.0001. |
| Fitzpatrick, 2011 (66) | Ireland | 57. Fund and contract for the clinical innovation | Cohort study. | **Smoking prevalence:**  **2-year follow up cohort:** n (with data at baseline and 2-years) = 5,430. Baseline: 13.9% smoking. 1-year: 11.2% smoking. 2-year: 10.4% smoking. Statistically significant difference between 2-year and baseline: -3.5%, p<0.0001.  **3.5-year follow up cohort:** n (with data at baseline and 3.5-years) = 2,078. Baseline: 12.8% smoking. 1-year: 11.7% smoking. 2-year: 9.8% smoking. 3-year: 10.0% smoking. 3.5-year: 9.9% smoking. Statistically significant difference between 3.5-year and baseline: -2.9%, p<0.0001.  **Proportions of 'nonsmokers':** Proportion (%) read off the graph, Figure 1a. Baseline: 85%. 1-year: 88%. 2-year: 90%. 3-year: 90%. 3.5-year: 90.1%. Relative increase of 22.7% in proportion of nonsmokers from baseline (to 90.1%, p<0.0001).  **Prescription for smoking cessation medication:** 3.5-year: "23.5% of smokers were prescribed smoking cessation medication." |
| Forster, 2016 (48) | England | 57. Fund and contract for the clinical innovation | Cohort study. | **Record of smoking status:** NHS health check: 0% of men (n=20) and 0% of women (n=10) did not have smoking status recorded. Controls: 7% of men (n=6,321) and 2% of women (n=1,473) did not have smoking status recorded. Net reduction in proportion with no smoking status: 7% in men and 2% in women (p<0.001). Reduction in deprivation inequality was greater for men (4% for smoking records), compared to 1% for women. **Smoking prevalence:** NHS health check: 21% of men (n=7,775) and 16% of women (n=6,300) had 'current smoking detected'. Controls: 26% of men (n=26,841) and 21% of women (n=19,071) had 'current smoking detected'. Deprivation inequality reduced by 1% in men and by 4% in women. |
| Frijling, 2003 (69) | Netherlands | 57. Fund and contract for the clinical innovation | Controlled before-and-after trial. | **Record of smoking status:** **Results not available for 'smoking habits' only.  **Record of cessation counselling:**  Intervention practices:  Baseline: 27.3% (n=84/308) Post-intervention: 37% (n=114/308) Difference: 9.7%, 95% CI: 3.2 to 16.3.  Control practices: Baseline: 23.2% (n = 69/297) Post-intervention: 28.3% (n = 84/297) Difference: 5.1%, 95% CI: -0.6 to 10.7.  Adjusted odds ratio: 1.45, 95% CI: 1.02 to 2.07. |
| Pajak, 2010 (73) | Poland | 57. Fund and contract for the clinical innovation | Cohort study. | **Record of smoking status:** Before screening period: 12.3% (95% CI: 7.2 to 20.1) of patients had this information available in their medical records before the PCVDP, at the active clinics (n=3,940). Before screening period: 8.0% (95% CI: 4.6 to 13.6) of patients had this information available in their medical records before the PCVDP, at the non-active clinics (n=3,162). Difference between the two groups: non-significant, p=0.82.  After screening period: 32.9% (95% CI: 22.8 to 45.0) of patients had this information available in their medical records after the PCVDP, at the active clinics (n=3,940). After screening period: 10.1% (95% CI: 6.3 to 15.8) of patients had this information available in their medical records after the PCVDP, at the non-active clinics (n=3,162). Difference between the two groups: significant, p<0.001.  **Recall of receiving any cessation intervention ("tobacco cessation"):** Percentage of participants who received advice to change lifestyle prior to final examination (adjusted for age, sex and design effects): Active clinics (n=2,314): 41.0% (95% CI: 31.0 to 51.7) Non-active clinics (n=2,107): 34.6% (95% CI: 25.5 to 44.9) Difference between the two groups: non-significant, p=0.35.  **Recall of verbal advice or receipt of leaflets:** Percentage of participants who received advice to change lifestyle prior to final examination (adjusted for age, sex and design effects): Active clinics (n=2,314): 37.7% (95% CI: 27.6 to 49.0) Non-active clinics (n=2,107): 29.8% (95% CI: 21.1 to 40.3) Difference between the two groups: non-significant, p=0.25.  **Recall of referral to specialist clinic:** Percentage of participants who received advice to change lifestyle prior to final examination (adjusted for age, sex and design effects): Active clinics (n=2,314): 2.5% (95% CI: 1.2 to 5.1) Non-active clinics (n=2,107): 4.4% (95% CI: 2.3 to 8.2) Difference between the two groups: non-significant, p=0.25.  **Recall of prescription for pharmacotherapy:** Percentage of participants who received advice to change lifestyle prior to final examination (adjusted for age, sex and design effects): Active clinics (n=2,314): 5.8% (95% CI: 3.4 to 9.7) Non-active clinics (n=2,107): 5.8% (95% CI: 3.4 to 9.8) Difference between the two groups: non-significant, p=0.97.  **Recall of discussion about "other methods":** Percentage of participants who received advice to change lifestyle prior to final examination (adjusted for age, sex and design effects): Active clinics (n=2,314): 3.8% (95% CI: 2.1 to 6.7) Non-active clinics (n=2,107): 3.5% (95% CI: 1.9 to 6.3) Difference between the two groups: non-significant, p=0.84.  **Non-smoking prevalence:** Percentage of participants who were "not smoking" at the final examination (adjusted for age, sex and design effects): Active clinics (n=2,314): 73.9% (95% CI: 65.7 to 80.7) Non-active clinics (n=2,107): 65.4% (95% CI: 56.6 to 73.2) Difference between the two groups: non-significant, p=0.29. |
| Wright, 2018 (71) | Australia | 57. Fund and contract for the clinical innovation | Cohort study. | **Record of smoking status:** TIS-funded services: 2014: 85% of clients asked about their tobacco use. 2016: 88% of clients asked about their tobacco use.  Non-TIS-funded services: 2014: 84% of clients asked about their tobacco use. 2016: 80% of clients asked about their tobacco use.  Among TIS-funded services, the tobacco use reporting ratio (RR) was 1.58-fold higher (95% CI: 1.30 to 1.91; p<0.001) after controlling for remoteness, year of funding, and interactions.  **Smoking prevalence:** 53% of all clients attending all services in 2014, 2015, 2016 were reported as current smokers. 14% in 2014 and 2015, 15% in 2016 were reported as ex-smokers. 33% in 2014, 32% in 2015 and 2016 were reported as non-smokers. TIS funding was not associated with any change in reporting of clients as current smokers, ex-smokers or non-smokers across the three reporting periods (2014, 2015, 2016). |
| Bailey, 2016 (54) | Oregon, USA | 59. Place innovation on fee for service lists/formularies | Cohort study. | **Prescription of cessation medication (NRT, varenicline, bupropion):** Gained Medicaid: 26.9% (n=1,115/4,140) of smokers had a cessation medication ordered. Uninsured: 11.5% (n=477/4,140) of smokers had a cessation medication ordered. Statistically significant difference, p<0.001. The odds of having medication ordered were almost 3 times higher for patients who gained Medicaid relative to the uninsured cohort (aOR: 2.94, 95% CI: 2.61 to 3.32).  **Cessation:**  Gained Medicaid: 16.6% (n=686/4,140) of smokers quit smoking during the study periods. Uninsured: 13.3% (n=550/4,140) of smokers quit smoking during the study periods. Statistically significant difference, p<0.001. The newly insured (gained Medicaid) had 40% increased odds of quitting compared to their uninsured counterparts (aOR: 1.40, 95% CI: 1.24 to 1.58).  Among patients without a smoking medication ordered, the gained Medicaid group had significantly higher odds of quitting compared to the group of uninsured smokers (aOR: 1.23, 95% CI: 1.06 to 1.41). Among patients with medication ordered, the odds of quitting was also higher for those who gained Medicaid, but the difference was not significant in this smaller group (aOR: 1.29, 95% CI: 0.99 to 1.67).  Among patients with more follow-up visits, the odds of quitting were 22% higher for those who gained Medicaid (aOR: 1.22, 95% CI: 1.05 to 1.42); there were no significant between-group differences in quit rates among patients with <6 follow-up visits.  Within-group analyses: Having a smoking cessation medication order resulted in higher odds of quitting smoking for both groups (newly insured: aOR: 2.00, 95% CI: 1.69 to 2.37; uninsured: aOR: 1.90, 95% CI: 1.50 to 2.41). Patients with more visits had higher odds of quitting than patients with fewer visits (newly insured: aOR: 2.86, 95% CI: 2.36 to 3.45; uninsured: aOR: 2.60, 95% CI: 2.16 to 3.12). |
| Bailey, 2020 (60) | United States (multi-state) | 59. Place innovation on fee for service lists/formularies | Cohort study. | **Prescription of cessation medication (NRT, varenicline, bupropion):** Adjusted odds ratio (over 24 months): Non-expansion: 1.00 (reference group). Expansion: 1.53, 95% CI: 1.44 to 1.62 (p<0.001).  **Cessation:**  Adjusted odds ratio (over 24 months): Non-expansion: 1.00 (reference group). Expansion: 1.35, 95% CI: 1.28 to 1.43 (p<0.001).  "Among patients with a cessation medication ordered, those from expansion states had 65% higher odds of quitting compared to those from non-expansion states (aOR: 1.65, 95% CI: 1.48 to 1.84); the odds of quitting among those without a cessation medication ordered were 29% higher for patients in expansion versus non-expansion states."  "For patients in expansion states, the odds of quitting were higher regardless of follow-up visit numbers or percent of federal poverty level (FPL) at baseline, compared to those in non-expansion states." Follow-up visits (<6): (aOR: 1.30, 95% CI: 1.21 to 1.40); Follow-up visits (6+): (aOR: 1.25, 95% CI: 1.16 to 1.34); FPL ≤138%: (aOR: 1.32, 95% CI: 1.23 to 1.41); FPL >138%: (aOR: 1.21, 95% CI: 1.07 to 1.37).  "Among patients who were uninsured at baseline, those in expansion states had 51% higher odds of quitting than those from states that did not expand (aOR: 1.51, 95% CI: 1.39 to 1.64); the odds of quitting among those that were insured at baseline were also higher for patients in expansion versus non-expansion states, although of lesser magnitude (aOR: 1.29, 95% CI: 1.21 to 1.37)." |
| Li, 2018 (61) | United States (multi-state) | 59. Place innovation on fee for service lists/formularies | Repeated cross-sectional study.  Descriptive. | Proportion (%) read off the graph, Figure 2.  **Record of smoking status:** 2010: 59.2% (of patients who had a documentation of smoking history). 2011: 64.3% 2012: 67.8% 2013: 70.3% 2014: 73.6% 2015: 76.5% 2016: 77.8% |
| Marino, 2016 (62) | Oregon, USA | 59. Place innovation on fee for service lists/formularies | Cohort study. | **Record of smoking status** ('screening for smoking'):  "Individuals randomly selected to apply for insurance did not always follow through, and thus remained uninsured." Both 'selected to apply for coverage' and 'gained coverage' results outlined.  Intervention: 'Selected to apply for coverage'. Selected: 59.2% (n=4,049) Not selected (ref): 56.9% (n=6,594) Difference: 2.3%. OR: 1.07, 95% CI: 1.04 to 1.10. AOR: 1.04, 95% CI: 1.02 to 1.06.  Intervention: 'Gained coverage', n=1,718 (44% of n=4,049). Mean value in control group: 56.4%, 95% CI: 53.0 to 59.7.  6.2% change with Medicaid coverage, 95% CI: 5.3 to 7.1, p<0.001. |
| Miraldo, 2018 (57) | Massachusetts, USA | 59. Place innovation on fee for service lists/formularies | Repeated cross-sectional study with control.  Difference-in-differences (DD) and triple differences (DDD) design. | **Quit attempt** ("attempted to quit smoking"): DD coefficient for whole sample: 0.001 (standard error: 0.018). Not statistically significant. DDD coefficient for whole sample vs adults above 300% FPL: 0.004 (standard error: 0.029). Not statistically significant. |
| Parnes, 2002 (58) | Colorado, USA | 59. Place innovation on fee for service lists/formularies | Cross-sectional study (with control group).  Analytical. | **Smoking prevalence:** Total number of smokers: n=351/1,443 (24%). OR for smoking. Uninsured: 1.00 (reference group). Medicaid: 1.01, 95% CI: 0.73 to 1.4 (p=0.937). Private/Health Maintenance Organisation (HMO): 0.55, 95% CI: 0.41 to 0.73 (p<0.001).  **Record of cessation counselling:** Total number of smokers who received smoking cessation counselling: n=129/351 (37%). OR of receiving cessation counselling. Uninsured: 1.00 (reference group). Medicaid: 2.1, 95% CI: 1.2 to 3.7 (p=0.011). Private/Health Maintenance Organisation (HMO): 3.0, 95% CI: 1.8 to 5.3 (p<0.001). |
| Tilson, 2004 (63) | Ireland | 59. Place innovation on fee for service lists/formularies | Repeated cross-sectional study.  Descriptive. | **Prescription for NRT (dispensed?):** May 2001 (free prescriptions for NRT were introduced in April 2001): 6 per 1,000 patients were prescribed NRT. 2002: n=47,147/49,826 (94.6%) patients who received smoking cessation products in 2002 were prescribed NRT. **More detailed outcome data is missing in the paper.  **Prescription for bupropion (dispensed?):** January 2001 (before introduction of free prescriptions for NRT in April 2001): 6 per 1,000 patients were prescribed bupropion. June 2001: 1 per 1,000 patients were prescribed bupropion. 2002: n=2,679/49,826 (5.4%) patients who received smoking cessation products in 2002 were prescribed bupropion. **More detailed outcome data is missing in the paper. |
| Williams, 2004 (64) | Ireland | 59. Place innovation on fee for service lists/formularies | Repeated cross-sectional study.  Descriptive. | Proportion read off the graph, Figure 1. **Prescription for bupropion (dispensed?):** Sept 2000: 3.1 per 1,000 patients. Oct 2000: 6.1 per 1,000 patients. Nov 2000: 4.8 per 1,000 patients. Dec 2000: 3 per 1,000 patients. Jan 2001: 6 per 1,000 patients. Feb 2001: 5 per 1,000 patients. Mar 2001: 2.5 per 1,000 patients. Apr 2001: 1.8 per 1,000 patients. May 2001: 1 per 1,000 patients. Jun to Dec 2001: <1 per 1,000 patients.  **Prescription for NRT (dispensed?)** (Introduction of NRT to GMS: Apr 2001): Sept 2000 to March 2001: 0 per 1,000 patients. Apr 2001: 6.1 per 1,000 patients. May 2001: 7.2 per 1,000 patients. Jun 2001: 7 per 1,000 patients. Jul 2001: 6.2 per 1,000 patients. Aug 2001: 5.9 per 1,000 patients. Sept 2001: 6.5 per 1,000 patients. Oct 2001: 7.5 per 1,000 patients. Nov 2001: 7.2 per 1,000 patients. Dec 2001: 7.2 per 1,000 patients. |
| Coleman, 2007 (32) | UK | 60. Alter incentive/allowance structures | Repeated cross-sectional study.  Analytical. | **Record of smoking status:** "Compared to the first quarter of 2003, there was an increase up to the first quarter of 2004 in recording of smoking status (RR 1.88, 95% CI 1.87–1.89), which was sustained until the first quarter of 2005."  **Record of cessation advice:** "Compared to the first quarter of 2003, there was an increase up to the first quarter of 2004 in brief advice to smokers (RR 3.03, 95% CI 2.98–3.09), which was sustained until the first quarter of 2005."  **Prescription for NRT/bupropion:** "The incidence of receiving nicotine addiction treatments increased after the year in which these became available on prescription from UK GPs (2000 for bupropion and 2001 for nicotine replacement therapy), but there was no consistent change in this index of smoking cessation activity in the period leading up to or following the introduction of the contract."  "for all patients, temporary increases in the recording of smoking status and (for smokers) brief advice between 1993 and 1995 and sustained increases from around the turn of the millennium with an acceleration in this trend from 2003."  "The absolute increase in the annual incidence of recording smoking status from the year 2000 was more marked in the disease-specific cohorts (those with a diagnosis of COPD, ischaemic heart disease or diabetes). This was slightly lower for those with a diagnosis of stroke/TIA, hypertension and asthma." "the absolute increase is seen to be much greater in those with one of these conditions than in all patients, but was nevertheless also increased in patients who did not have a diagnosis of the six conditions listed in the GP contract." "prescriptions for nicotine addiction treatments increased steadily in all six ‘diseased’ cohorts and also in the ‘healthy’ cohort from 2001, without any acceleration around the introduction of the new contract in any cohort." |
| Dhalwani, 2013 (38) | UK | 60. Alter incentive/allowance structures | Repeated cross-sectional study.  Descriptive. | Proportion (%) read off the graph, Figure 1.  **Record of smoking status** (imputing data based on QOF): 2000: ~11% of pregnancies with recording of smoking status during gestation.  2001: ~13% 2002: ~15% 2003: ~21% 2004: ~36% 2005: ~39% 2006: ~38% 2007: ~44% 2008: ~43% 2009: ~49% |
| Farley, 2017 (40) | UK | 60. Alter incentive/allowance structures | Cohort study. | **Updating of smoking status:** "Cancer patients were significantly less likely to have their smoking status updated during the first year after diagnosis than control patients (37% vs 78%)." (All cancer patients vs CHD controls, OR: 0.18, (95% CI: 0.17 to 0.19.) All cancers: Pre-QOF: 19% (n=398/2,057) of all cancer patients had a smoking status update within the first year after diagnosis. Post-QOF: 40% (n=4,143/10,336) of all cancer patients had a smoking status update within the first year after diagnosis. CHD control: Pre-QOF: 61% (n=1,282/2,116) of all CHD patients had a smoking status update within the first year after diagnosis. Post-QOF: 81% (n=8,345/10,277) of all CHD patients had a smoking status update within the first year after diagnosis. Adjusted OR for post-QOF/pre-QOF for cancer patients and CHD control patients combined: 2.71; 95% CI: 2.44 to 2.99.  No statistically significant difference between the change for cancer patients vs the change for CHD patients: p=0.86.  **Record of cessation advice:** "Cancer patients were significantly less likely to have a recording of advice to quit (all cancer patients vs CHD controls, OR: 0.38, (95% CI: 0.36 to 0.40))."  All cancers: Pre-QOF: 8% (n=166/2,057) of all cancer patients had a record of cessation advice within the first year after diagnosis. Post-QOF: 25% (n=2,628/10,336) of all cancer patients had a record of cessation advice within the first year after diagnosis. CHD control: Pre-QOF: 24% (n=509/2,116) of all CHD patients had a record of cessation advice within the first year after diagnosis. Post-QOF: 49% (n=5,092/10,277) of all CHD patients had a record of cessation advice within the first year after diagnosis. Adjusted OR for post-QOF/pre-QOF for cancer patients and CHD control patients combined: 3.04; 95% CI: 2.73 to 3.38.  Statistically significant difference between the change for cancer patients vs the change for CHD patients: p=0.02.  **Prescription of smoking cessation medications:** "Cancer patients were significantly less likely to be prescribed smoking cessation medications (all cancer patients vs CHD controls, OR: 0.67, (95% CI: 0.63 to 0.73)." All cancers: Pre-QOF: 13% (n=285/2,116) of all cancer patients received a prescription for smoking cessation medication within the first year after diagnosis. Post-QOF: 22% (n=2,275/10,277) of all cancer patients received a prescription for smoking cessation medication within the first year after diagnosis. CHD control: Pre-QOF: 8% (n=165/2,057) of all CHD patients received a prescription for smoking cessation medication within the first year after diagnosis. Post-QOF: 13% (n=1,339/10,336) of all CHD patients received a prescription for smoking cessation medication within the first year after diagnosis. Adjusted OR for post-QOF/pre-QOF for cancer patients and CHD control patients combined: 1.79; 95% CI: 1.56 to 2.05.  No statistically significant difference between the change for cancer patients vs the change for CHD patients: p=0.89.  **Smoking cessation 1 year-post cancer/CHD diagnosis:** "Of the 3,706 cancer and CHD patients who smoked at diagnosis and had at least 1 smoking status update in the year following diagnosis, 1,359 (36.7%) of patients with cancer and 1,645 (44.4%) of patients with CHD stopped smoking (OR: 0.76; 95% CI: 0.69 to 0.84). Among 2,253 pairs, both of whom had smoking status updated and survived at least 1 year, 863 (38.3%) with cancer and 1,004 (44.6%) with CHD stopped smoking (OR: 0.82; 95% CI: 0.72 to 0.93)." All cancers: Pre-QOF: 33.95% (n=110/324) of all cancer patients quit smoking within the first year after diagnosis. Post-QOF: 36.9% (n=1,249/3,382) of all cancer patients quit smoking within the first year after diagnosis. CHD control: Pre-QOF: 40.9% (n=139/340) of all CHD patients quit smoking within the first year after diagnosis. Post-QOF: 44.7% (n=1,506/3,366) of all CHD patients quit smoking within the first year after diagnosis. Adjusted OR for post-QOF/pre-QOF for cancer patients and CHD control patients combined: 1.18; 95% CI: 0.94 to 1.49.  No statistically significant difference between the change for cancer patients vs the change for CHD patients: p=0.95. |
| Fichera, 2016 (45) | England | 60. Alter incentive/allowance structures | Repeated cross-sectional study.   Regression discontinuity design (with control). | **Number of cigarettes smoked per day** (n=10,924): Before: 3.80 After: 3.20 Statistically significant difference, p=0.004  Local linear regression, optimal bandwidth (3.4 years): coefficient: -0.70 (SD: 0.29), p<0.01. (n=19,663).  **Recall of receiving cessation advice:** Polynomial regression, Model 1 (with best polynomial order): coefficient 0.02 (SD: 0.02), not statistically significant. (n=21,418)  **Recall of receiving prescription for cessation medication:** Polynomial regression, Model 1 (with best polynomial order): coefficient -0.04 (SD: 0.04), not statistically significant. (n=23,346) |
| Hardy, 2014 (39) | UK | 60. Alter incentive/allowance structures | Repeated cross-sectional study.  Descriptive. | Proportion (%) read off the graph, Figure 1.  **Record of cessation advice:** 2000: ~7% of pregnant smokers recorded to be given smoking cessation advice.  2001: ~8% 2002: ~11% 2003: ~15% 2004: ~33% 2005: ~37% 2006: ~26% 2007: ~29% 2008: ~26% 2009: ~29% |
| McGovern, 2008 (43) | Scotland | 60. Alter incentive/allowance structures | Repeated cross-sectional study.  Analytical. | **Record of smoking status:** Pre-contract (March 2004): 69.5% (n=35,095) of patients had smoking status recorded. Post-contract (March 2005): 95.7% (n=71,747) of patients had smoking status recorded. Statistically significant increase, p<0.05. **Patients with missing data (e.g. smoking status) were excluded from the analysis of that factor.  **Record of cessation advice:** Pre-contract (March 2004): 81.0% (n=9,904) of smokers given advice. Post-contract (March 2005): 96.2% (n=31,881) of smokers given advice. Statistically significant increase, p<0.05. **Patients with missing data (e.g. smoking status) were excluded from the analysis of that factor. |
| Millett, 2007 (44) | UK | 60. Alter incentive/allowance structures | Cohort study. | **Record of smoking status:** 2003: 90.0% of patients with diabetes had ever had smoking status recorded. 2005: 98.8% of patients with diabetes had ever had smoking status recorded. Statistically significant increase, p<0.001.  2003: 67.6% of patients with diabetes whose smoking status was recorded in the 15 months before the 2003 study period. 2005: 86.7% of patients with diabetes whose smoking status was recorded in the 15 months before the 2005 study period. Statistically significant increase, p<0.001.  **Record of cessation advice:** 2003: 48.0% of patients with diabetes who were given smoking cessation advice in the 15 months before the 2003 study period. 2005: 83.5% of patients with diabetes who were given smoking cessation advice in the 15 months before the 2005 study period. Statistically significant increase, p<0.001.  **Smoking prevalence:** 2003: 20.0% of patients with diabetes who were smokers during the 2003 study period. 2005: 16.2% of patients with diabetes who were smokers during the 2005 study period. Statistically significant decrease, p<0.001. |
| Simpson, 2006 (49) | Scotland | 60. Alter incentive/allowance structures | Repeated cross-sectional study.  Analytical. | **Record of smoking status:** Precontract: 41.1% (n=8,990) of patients with a history of stroke/TIA who had a recording of smoking status. Postcontract: 90.6% (n=27,019) of patients with a history of stroke/TIA who had a recording of smoking status. Difference: 49.4%, 95% CI: 48.7 to 50.2.  **Record of cessation advice:** Precontract: 79.0% (n=3,081) of patients with a history of stroke/TIA who smoke who had a record of cessation advice. Postcontract: 95.9% (n=13,021) of patients with a history of stroke/TIA who smoke who had a record of cessation advice. Difference: 17.0%, 95% CI: 15.7 to 18.3. |
| Sutton, 2010 (47) | Scotland | 60. Alter incentive/allowance structures | Repeated cross-sectional study.  Analytical. | **Record of smoking status:** "Rates of recording are higher than the reference risk factor (alcohol consumption) for blood pressure, smoking status and BMI." Model 1: Coefficient of 'smoking status' is 0.480 (z: 202.2), where the reference category is 'alcohol status' (which was not incentivised by the QOF scheme).  However: The coefficient on the dummy variable indicating that a disease-factor is incentivised is reduced when a variable is introduced to reflect the higher rates of recording of the incentivised disease-factor combinations prior to the introduction of the QOF (Model (2)) (Smoking status coefficient becomes -0.132 (z: -32.4)). The effect is further reduced when the variables capturing the dynamic process are introduced (Model (3)) (Smoking status coefficient becomes -0.138 (z: -33.8)). |
| Szatkowski, 2010 (29) | UK | 60. Alter incentive/allowance structures | Repeated cross-sectional study.  Descriptive. | **Record of smoking status:** The proportion of new patients annually who have their smoking status recorded within 90 days of registration has steadily increased between 1990 and 2006. 1990: 25.8% of patients had their smoking status recorded at registration, but 63.1% of patients lacked a recording of smoking status 1 year after registration. 2006: 73.3% of new patients had their smoking status recorded within 90 days of registering, but 16.6% patients (19.4% men and 14.1% women) lacked a recording of smoking status 1 year after registration.  "In all years, there was considerable variation between practices in the recording of recently registered patients’ smoking status; e.g. in 2006, while one practice recorded the smoking status of all its new patients, the worst performer did so in just 7.8% of cases (IQR: 62.5% to 88.2%)." |
| Szatkowski, 2011 (28) | England | 60. Alter incentive/allowance structures | Repeated cross-sectional study.  Descriptive. | Proportion (%) read off the graph, Figure 1.  THIN: **Record of cessation advice:**  2000-2003: <3% of patients had a record of cessation advice.  2004: ~7% of patients had a record of cessation advice. 2005-2009: ~10% of patients had a record of cessation advice. (10.9% in 2009.) "Majority of increase occurred between 2003 and 2005."  PCT Patient Survey: **Recall of receiving cessation advice:** 2004: 6.6% of patients had recalled receiving cessation advice. 2005: ~7% of patients had recalled receiving cessation advice. 2008: 8.3% of patients had recalled receiving cessation advice. |
| Szatkowski, 2016 (27) | England | 60. Alter incentive/allowance structures | Repeated cross-sectional study.  Interrupted time series analysis (no control). | Two analyses reported:  Pre: Apr 2004 to Mar 2012, Post: Apr 2012 to Apr 2013.  **Record of cessation advice:** 19.6% change, 95% CI: 7.9 to 31.4, p<0.001. **Referral to NHS Stop Smoking Service:** 38.8% change, 95% CI: 15.2 to 62.4, p<0.001. **Prescription for pharmacotherapy (NRT/bupropion/varenicline):** -7.7% change, 95% CI: -21.6 to 6.2, p=0.280.  Pre: Apr 2010 to Mar 2012, Post: Apr 2012 to Apr 2013. **Record of cessation advice:** 18.9% change, 95% CI: 9.9 to 27.9, p<0.001. **Referral to NHS Stop Smoking Service:** 38.1% change, 95% CI: 19.3 to 57.0, p<0.001. **Prescription for pharmacotherapy (NRT/bupropion/varenicline):** -13.8% change, 95% CI: -21.0 to -6.5, p<0.001. |
| Taggar, 2012 (30) | UK | 60. Alter incentive/allowance structures | Repeated cross-sectional study.  Descriptive. | Proportion (%) read off the graph, Figure 1. **Record of smoking status:** 2000: ~19% of patients had a record of smoking status.  2001: ~20% 2002: ~26% 2003: ~31% 2004: ~45% 2005: ~57% 2006: ~59% 2007: ~63% 2008: 64.5%  **Record of cessation advice:** 2000: ~6% of current smoker patients had a record of cessation advice.  2001: ~9% 2002: ~11% 2003: ~12% 2004: ~32% 2005: ~45% 2006: ~42% 2007: ~49% 2008: 50.5%  "A substantial acceleration in recording of both smoking status and cessation advice was observed between 2003 and 2005, although rates of increase plateaued after 2006. " |
| Tahrani, 2007 (42) | England | 60. Alter incentive/allowance structures | Repeated cross-sectional study.  Analytical. | **Record of smoking status:** April 2004 (pre-intervention): mean 44% (SD: 14). March 2005 (post-intervention): mean 96% (SD: 4). March 2006 (post-intervention): mean 95% (SD: 4). Difference in means between (April 2004) and (March 2006): 95% CI: -54.7 to -47.3, p<0.001.  **Record of cessation advice:** April 2004 (pre-intervention): N/A. March 2005 (post-intervention): mean 95% (SD: 7). March 2006 (post-intervention): mean 96% (SD: 5). Difference in means between (October 2004) and (March 2006), as data for April 2004 was not available: 95% CI: -15.2 to -9.2, p<0.001. **Missing: October 2004 value. |
| Donner-Banzhoff, 1996 (75) | Germany vs UK | 65. Use capitated payments | Cross-sectional study (comparing two groups).  Analytical. | **Recall receiving "any cessation intervention":** Germany (Fee-For-Service): 55.7% of current and ex-smokers (n=103/185). UK (Capitation): 51.7% of current and ex-smokers (n=108/209). Not statistically significant difference, OR: 1.17, 95% CI: 0.79 to 1.75.  Germany (Fee-For-Service): 64.8% of current smokers (n=68/105). UK (Capitation): 64.7% of current smokers (n=77/119). Not statistically significant difference, OR: 1.0, 95% CI: 0.58 to 1.74.  **Recall of receiving cessation "advice once":** OR: 1.9, 95% CI: 1.2 to 3.1. (n=386 smokers and ex-smokers in Germany and the UK).  **Recall of receiving cessation "advice several times":** OR: 0.7, 95% CI: 0.4 to 1.0. (n=386 smokers and ex-smokers in Germany and the UK).  **Recall of receiving "nicotine patch/gum":** OR: 1.0, 95% CI: 0.4 to 2.6. (n=386 smokers and ex-smokers in Germany and the UK). |
| Domain 9. Change infrastructure | | | | |
| Szatkowski, 2021 (36) | England | 66. Mandate change | Repeated cross-sectional study.  Segmented regression analysis, no control. | **Prescription for any NRT:** Absolute annual percentage change in prescribing 2005 to 2012: -0.25, 95% CI: -0.36 to -0.15, p<0.001. Percentage change in trend from 2012 to 2013: -1.125, 95% CI: -1.35 to -0.88, p<0.001. Absolute annual percentage change in prescribing 2013 to 2017 (annual change 2005 to 2012 + change in trend 2012 to 2013): -1.37, 95% CI: -1.52 to -1.21, p<0.001.  **Prescription for dual NRT:** Absolute annual percentage change in prescribing 2005 to 2012: 0.34, 95% CI: 0.26 to 0.42, p<0.001. Percentage change in trend from 2012 to 2013: -0.76, 95% CI: -0.93 to -0.60, p<0.001. Absolute annual percentage change in prescribing 2013 to 2017 (annual change 2005 to 2012 + change in trend 2012 to 2013): -0.42, 95% CI: -0.53 to -0.31, p<0.001. |
| Dhalwani, 2014 (41) | UK | 69. Create or change credentialing and/or licensure standards | Repeated cross-sectional study.  Descriptive. | Proportion (%) read off the graph, Figure 2.  **Prescription for NRT in all pregnancies, during pregnancy:** 2001: ~0% (prescribing prevalence of NRT in all pregnancies) 2002: ~0.5% 2003: ~1% 2004: ~1.8% 2005: ~2.6% 2006: ~2.7% 2007: ~2.6% 2008: ~2.4% 2009: ~2.5% 2010: ~2.3% 2011: ~2.5% 2012: ~2.3%  **Prescription for NRT in all pregnant smokers, during pregnancy:** 2001: ~0.7% (prescribing prevalence of NRT in pregnant smokers) 2002: ~7% 2003: ~10% 2004: ~11% 2005: ~11.4% 2006: ~12% 2007: ~11.4% 2008: ~10% 2009: ~11.3% 2010: ~10% 2011: ~10.5% 2012: ~10% |
| Langley, 2011 (34) | England | 69. Create or change credentialing and/or licensure standards | Repeated cross-sectional study.  Segmented regression analysis (no control). | Baseline trend (monthly change in number of prescriptions per 100,000 adolescents before licensing change); level change (step change in the monthly level of prescribing immediately after licensing change); trend change (absolute change in trend in monthly numbers of prescriptions per 100,000 adolescents after licensing change, compared with baseline trend). **Prescription for NRT:** All: Baseline trend: 1.36, 95% CI: 1.16 to 1.55, p<0.001. Level change: N/A. Trend change: -1.16, 95% CI: -1.52 to -0.79, p<0.001. 12-13 year olds: Baseline trend: 0.09, 95% CI: 0.07 to 0.12, p<0.001. Level change: N/A. Trend change: N/A. 14-15 year olds: Baseline trend: 1.08, 95% CI: 0.82 to 1.34, p<0.001. Level change: 19.29, 95% CI: 9.02 to 29.59, p<0.001. Trend change: -1.13, 95% CI: -1.51 to -0.74, p<0.001. 16-17 year olds: Baseline trend: 2.62, 95% CI: 2.16 to 3.08, p<0.001. Level change: N/A. Trend change: -2.73, 95% CI: -3.59 to -1.88, p<0.001. Females: Baseline trend: 1.66, 95% CI: 1.42 to 1.91, p<0.001. Level change: N/A. Trend change: -1.65, 95% CI: -2.10 to -1.20, p<0.001. Males: Baseline trend: 0.87, 95% CI: 0.59 to 1.16, p<0.001. Level change: 13.37, 95% CI: 2.21 to 24.52, p=0.02. Trend change: -0.76, 95% CI: -1.19 to -0.33, p<0.001. |
| Langley, 2012 (35) | England | 69. Create or change credentialing and/or licensure standards | Repeated cross-sectional study.  Segmented regression analysis (no control). | Baseline trend (monthly change in number of prescriptions per 100,000 patients before licensing change); level change (step change in the monthly level of prescribing immediately after licensing change); trend change (absolute change in trend in monthly numbers of prescriptions per 100,000 patients after licensing change, compared with baseline trend). **Prescription for NRT:** CHD: Baseline trend: 3.18, 95% CI: 2.15 to 4.21, p<0.0001. Level change: N/A. Trend change: -6.45, 95% CI: -8.36 to -4.53, p<0.0001. Stroke: Baseline trend: 3.37, 95% CI: 2.31 to 4.43, p<0.0001. Level change: N/A. Trend change: -5.99, 95% CI: -7.96 to -4.01, p<0.0001.  **Prescription for all licensed smoking cessation medications (NRT, varenicline, bupropion):** CHD: Baseline trend: 2.73, 95% CI: 1.23 to 4.25, p<0.001. Level change: N/A. Trend change: -3.07, 95% CI: -5.87 to -0.26, p=0.035. Stroke: Baseline trend: 3.29, 95% CI: 1.81 to 4.76, p<0.0001. Level change: N/A. Trend change: -3.76, 95% CI: -6 to -1.02, p=0.009. |
| Li, 2020 (55) | United States (multi-state) | 69. Create or change credentialing and/or licensure standards | Repeated cross-sectional study.  Analytical. | **Record of assist to quit (1)** ("referrals to smoking cessation programs" or "informal smoking cessation counselling"): **these outcome measures were grouped by the authors. Pre-guideline (2010-2013): 27.1% (n=1,513/5,580). Post-guideline (2014-2017): 27.0% (n=1,916/7,098). Not statistically significant change, p=0.87881. Model A (after controlling for age, sex, race/ethnicity, and level of smoking), post- versus pre-guideline OR: 1.29, 95% CI: 1.15 to 1.46, p<0.05.  **Record of assist to quit (2)** ("formal in-visit smoking cessation counselling"): Pre-guideline (2010-2013): 0.9% (n=49/5,580). Post-guideline (2014-2017): 2.7% (n=194/7,098). Statistically significant change, p<0.0001. Model A (after controlling for age, sex, race/ethnicity, and level of smoking), post- versus pre-guideline OR: 5.03, 95% CI: 3.05 to 8.30, p<0.05.  **Prescription for smoking cessation pharmacotherapy (bupropion, varenicline, NRT)** ("medication orders for pharmacotherapy"): Pre-guideline (2010-2013): 4.3% (n=238/5,580). Post-guideline (2014-2017): 5.2% (n=371/7,098). Statistically significant change, p=0.01196. Model A (after controlling for age, sex, race/ethnicity, and level of smoking), post- versus pre-guideline OR: 1.24, 95% CI: 1.02 to 1.50, p<0.05.  **Receipt of any smoking cessation intervention(s):** Pre-guideline (2010-2013): 30.6% (n=1,708/5,580). Post-guideline (2014-2017): 32.7% (n=2,323/7,098). Statistically significant change, p=0.01101. Model A (after controlling for age, sex, race/ethnicity, and level of smoking), post- versus pre-guideline OR: 1.44, 95% CI: 1.28 to 1.61, p<0.05.  **Cigarettes smoked per day:** Pre-guideline (2010-2013): 15.2 (SD: 10.5) (n=5,580). Post-guideline (2014-2017): 14.2 (SD: 9.9) (n=7,098). Statistically significant change, p<0.0001. |
| Thorndike, 2007 (53) | United States (multi-state) | 69. Create or change credentialing and/or licensure standards | Repeated cross-sectional study.  Analytical. | **Record of smoking status:** 1994-1996: 68% of primary care physicians identified patients' smoking status at all visits (weighted to reflect national estimates). 2001-2003: 70% of primary care physicians identified patients' smoking status at all visits (weighted to reflect national estimates). Adjusted OR: 1.10, 95% CI: 0.94 to 1.32. (Adjusted for patient demographics, physician specialty, and diagnosis.)  **Record of cessation counselling:** 1994-1996: Primary care physicians recorded smoking counselling at 30% of smokers' visits (weighted to reflect national estimates). 2001-2003: Primary care physicians recorded smoking counselling at 26% of smokers' visits (weighted to reflect national estimates). Adjusted OR: 0.81, 95% CI: 0.65 to 1.00. (Adjusted for patient demographics, physician specialty, and diagnosis.)  **Prescription for pharmacotherapy (NRT and bupropion):** **Results not available for primary care physicians. |
| Peterson, 2016 (52) | United States (multi-state) | 71. Change accreditation or membership requirements | Repeated cross-sectional study.  Analytical. | **Record of cessation counselling** (physician-reported): **Raw data missing. "the rate of physician-reported counseling for ... smoking cessation was above 90% post-intervention." **Recall of receiving cessation counselling** (patient questionnaire): (n=7,319) Pre: 92.5% of patients who smoke report that their "doctor talked to you about quitting". Post: 96.1% of patients who smoke report that their "doctor talked to you about quitting". Statistically significant difference: p<0.05. "Small increases were seen for quality measures that were already at high levels prior to the intervention with the percentage of patients who reported receiving smoking cessation counseling increasing from 92.5% to 96.1%." |
| Shi, 2017 (59) | United States (multi-state) | 71. Change accreditation or membership requirements | Cross-sectional study (with control group).  Analytical. | **Record of smoking status:** PCMH recognition in 2012 (n=539 practices): 87.64% (0.69) of adults assessed for tobacco use. No PCMH recognition in 2012 (n=548 practices): 83.85% (0.81) of adults assessed for tobacco use. Statistically significant difference: p<0.001. Regression coefficient: 3.0079 (SE: 1.3256), p<0.05. (n=1,193). PCMH recognition status was positively associated with being assessed for tobacco use.  **Record of cessation intervention:** PCMH recognition in 2012 (n=539 practices): 59.9% (1.05) of adults who were known tobacco users that received tobacco cessation counselling and/or pharmacologic intervention. No PCMH recognition in 2012 (n=548 practices): 55.51% (1.14) of adults who were known tobacco users that received tobacco cessation counselling and/or pharmacologic intervention. Statistically significant difference: p<0.01. Regression coefficient: 3.7993 (SE: 1.7852), p<0.05. (n=1,175). PCMH recognition status was positively associated with receiving tobacco cessation intervention. |
| Van Doorn-Klomberg, 2014 (68) | Netherlands | 71. Change accreditation or membership requirements | Cohort study. | **Smoking prevalence:** 1st cohort, 2006-2008: 36.6% (SD: 22.9) of patients with COPD smoked. 1st cohort, 2009-2011: 31.8% (SD: 16.1) of patients with COPD smoked. Difference: -4.9% (95% CI: -11.5 to 1.8), p=0.15.  2nd cohort, 2009-2011: 32.2% (SD: 20.7) of patients with COPD smoked. Difference between 1st cohort 2009-2011 and 2nd cohort 2009-2011: -0.4% (95% CI: -6.9 to 6.2), p=0.92.  1st cohort, 2006-2008: not available. 1st cohort, 2009-2011: 12.6% (SD: 8.5) of patients with CVD smoked. Difference: not available. **"The inclusion criteria for patients with risk for cardiovascular disease changed towards the inclusion of patients with known cardiovascular disease only, which made a within-group comparison of the first cohort not justifiable."  2nd cohort, 2009-2011: 10.5% (SD: 7.8) of patients with CVD smoked. Difference between 1st cohort 2009-2011 and 2nd cohort 2009-2011: 1.9% (95% CI: -1.1 to 4.9), p=0.20.  **Record of smoking status:** 1st cohort, 2006-2008: 76.0% (SD: 21.4) of patients with COPD had known smoking status. 1st cohort, 2009-2011: 75.4% (SD: 23.2) of patients with COPD had known smoking status. Difference: -1.3% (95% CI: -8.1 to 5.6), p=0.71.  2nd cohort, 2009-2011: 69.4% (SD: 27.0) of patients with COPD had known smoking status. Difference between 1st cohort 2009-2011 and 2nd cohort 2009-2011: 6.1% (95% CI: -2.8 to 15.0), p=0.18.  1st cohort, 2006-2008: not available. 1st cohort, 2009-2011: 51.7% (SD: 26.6) of patients with CVD had known smoking status. Difference: not available. **"The inclusion criteria for patients with risk for cardiovascular disease changed towards the inclusion of patients with known cardiovascular disease only, which made a within-group comparison of the first cohort not justifiable."  2nd cohort, 2009-2011: 39.8% (SD: 25.5) of patients with CVD had known smoking status. Difference between 1st cohort 2009-2011 and 2nd cohort 2009-2011: 11.3% (95% CI: 1.9 to 20.8), p=0.02.  **Record of cessation advice:** 1st cohort, 2006-2008: 47.0% (SD: 33.9) of patients with COPD who smoked had a record of stop smoking advice. 1st cohort, 2009-2011: 69.8% (SD: 30.8) of patients with COPD who smoked had a record of stop smoking advice. Difference: 21.9% (95% CI: 8.7 to 34.9), p=0.002.  2nd cohort, 2009-2011: 65.2% (SD: 32.6) of patients with COPD who smoked had a record of stop smoking advice. Difference between 1st cohort 2009-2011 and 2nd cohort 2009-2011: 5.1% (95% CI: -10.1 to 20.2), p=0.51.  1st cohort, 2006-2008: not available. 1st cohort, 2009-2011: 66.7% (SD: 34.2) of patients with CVD had known smoking status. Difference: not available. **"The inclusion criteria for patients with risk for cardiovascular disease changed towards the inclusion of patients with known cardiovascular disease only, which made a within-group comparison of the first cohort not justifiable."  2nd cohort, 2009-2011: 51.1% (SD: 34.0) of patients with CVD had known smoking status. Difference between 1st cohort 2009-2011 and 2nd cohort 2009-2011: 13.2% (95% CI: -4.6 to 30.9), p=0.14. |
| Multiple domains | | | | |
| Akman, 2017 (72) | Turkey | Domain 8. 65. Use capitated payments  AND  Domain 9.  66. Mandate change, 67. Change record systems, 71. Change accreditation or membership requirements | Repeated cross-sectional study.  Analytical. | **Rate of cessation counselling:** Proportion of primary care doctors who report being "usually or almost always involved in smoking counselling during outpatient clinic" (From Supplementary Table 2): 1993: n=146 (82.0%) 2012: n=253 (84.6%) **Denominators are not included in the paper. The total number of respondents were n=199 in 1993 and n=299 in 2012 but it is not stated how many participants responded to the individual survey questions. Percentage change: +3.1 (p>0.05), not statistically significant. |
| Bailey, 2017 (50) | Oregon, USA | Domain 8. 60. Alter incentive/allowance structures  AND  Domain 9.  67. Change record systems | Repeated cross-sectional study.  Analytical. | **Record of smoking status:** 2010: 93.90% (n=52,019/55,398) of non-pregnant patients have smoking status assessed. 2012: 96.16% (n=58,282/60,610) of non-pregnant patients have smoking status assessed. 2014: 97.41% (n=64,981/66,712) of non-pregnant patients have smoking status assessed.  2014 vs 2010 (ref), adjusted OR: 2.52, 95% CI: 2.37 to 2.69, p<0.0001. 2012 vs 2010 (ref), adjusted OR: 1.54, 95% CI: 1.46 to 1.63, p<0.0001. 2014 vs 2012 (ref), adjusted OR: 1.58, 95% CI: 1.47 to 1.69, p<0.0001.  **Smoking prevalence:** 2010: 30.33% (n=16,802/55,398) of non-pregnant patients were current smokers. 2012: 29.09% (n=17,631/60,610) of non-pregnant patients were current smokers. 2014: 27.15% (n=18,111/66,712) of non-pregnant patients were current smokers.  2014 vs 2010 (ref), adjusted OR: 0.81, 95% CI: 0.79 to 0.83, p<0.0001. 2012 vs 2010 (ref), adjusted OR: 0.93, 95% CI: 0.90 to 0.96, p<0.0001. 2014 vs 2012 (ref), adjusted OR: 0.85, 95% CI: 0.79 to 0.82, p<0.0001.  **Record of cessation counselling:** 2010: 29.75% (n=4,998/16,802) of non-pregnant smoker patients were given counselling. 2012: 53.88% (n=9,500/17,631) of non-pregnant smoker patients were given counselling. 2014: 69.49% (n=12,585/18,110) of non-pregnant smoker patients were given counselling.  2014 vs 2010 (ref), adjusted OR: 7.76, 95% CI: 7.35 to 8.20, p<0.0001. 2012 vs 2010 (ref), adjusted OR: 3.66, 95% CI: 3.48 to 3.85, p<0.0001. 2014 vs 2012 (ref), adjusted OR: 2.24, 95% CI: 2.13 to 2.35, p<0.0001.  **Prescription for cessation medications (NRT, varenicline, bupropion):** 2010: 12.09% (n=2,032/16,802) of non-pregnant smoker patients were ordered cessation medication. 2012: 13.26% (n=2,338/17,631) of non-pregnant smoker patients were ordered cessation medication. 2014: 15.68% (n=2,839/18,110) of non-pregnant smoker patients were ordered cessation medication.  2014 vs 2010 (ref), adjusted OR: 1.15, 95% CI: 1.07 to 1.23, p<0.0001. 2012 vs 2010 (ref), adjusted OR: 1.00, 95% CI: 0.93 to 1.07, not statistically significant. 2014 vs 2012 (ref), adjusted OR: 1.1, 95% CI: 1.03 to 1.17, p<0.01.  **Record of cessation medication "ordered and/or discussed":** 2010: 27.60% (n=4,638/16,802) of non-pregnant smoker patients were ordered cessation medication and/or discussed cessation medication. 2012: 39.63% (n=6,987/17,631) of non-pregnant smoker patients were ordered cessation medication and/or discussed cessation medication. 2014: 48.30% (n=8,747/18,110) of non-pregnant smoker patients were ordered cessation medication and/or discussed cessation medication.  2014 vs 2010 (ref), adjusted OR: 2.25, 95% CI: 2.14 to 2.37, p<0.0001. 2012 vs 2010 (ref), adjusted OR: 1.65, 95% CI: 1.57 to 1.73, p<0.0001. 2014 vs 2012 (ref), adjusted OR: 1.38, 95% CI: 1.32 to 1.45, p<0.0001. |
| Fortmann, 2020 (56) | United States (multi-state) | Domain 8. 60. Alter incentive/allowance structures  AND  Domain 9.  71. Change accreditation or membership requirements | Cohort study.   Interrupted time series analysis (no control). | Proportion (%) read off the graph, Figure 1.  **Record of smoking status:** Smoking status documentation for all CHCs combined. 2006: ~30%. 2007: ~42%. 2008: ~45%. 2009: ~50%. 2010: ~50%. 2011: ~50%. 2012: ~80%. 2013: ~90%.  "The interrupted time series analysis showed that the increase in documentation rate between 2011 and 2012 of 21.3% (95% CI: 8.2% to 34.4%) from the current trend was statistically significant, p=0.011." |
| Langley, 2011 (37) | England | Domain 8. 59. Place innovation on fee for service lists/formularies  AND  Domain 9.  69. Create or change credentialing and/or licensure standards | Repeated cross-sectional study.  Interrupted time series analysis (no control). | **Prescription for NRT, varenicline or bupropion (all):** Change in prescribing (%) after the introduction of varenicline: -0.42, 95% CI: -3.10 to 2.27, p=0.760. Change in prescribing (%) after the NICE guidance on varenicline: -1.72, 95% CI: -3.96 to 0.53, p=0.134.  **Prescription for NRT:** Change in prescribing (%) after the introduction of varenicline: -0.31, 95% CI: -3.11 to 2.49, p=0.828. Change in prescribing (%) after the NICE guidance on varenicline: -1.78, 95% CI: -4.26 to 0.69, p=0.159.  **Prescription for bupropion:** Change in prescribing (%) after the introduction of varenicline: -1.17, 95% CI: -3.90 to 1.56, p=0.401. Change in prescribing (%) after the NICE guidance on varenicline: -2.80, 95% CI: -6.22 to 0.61, p=0.108. |
| Mullins, 2009 (51) | Delaware, USA | Domain 5. 40. Distribute educational materials. 42. Conduct educational meetings  AND  Domain 7.  54. Prepare patients/consumers to be active participants | Repeated cross-sectional study.  Analytical. | **Smoking prevalence:** Pre-intervention group: 221 out of 922 patients (24.0%) had 'current smoking status' in their electronic health record. Post-intervention group: 547 out of 3,125 patients (17.3%) had 'current smoking status' in their electronic health record. Statistically significant difference, p=0.001.  **Record of cessation advice:** Pre-intervention group: 155 out of 221 current smokers (70.1%) had 'advised to quit', 'yes' in their electronic health record. Post-intervention group: 538 out of 547 current smokers (98.3%) had 'advised to quit', 'yes' in their electronic health record. Statistically significant difference, p=0.001. |
| Verbiest, 2013 (67) | Netherlands | Domain 8. 59. Place innovation on fee for service lists/formularies  AND  Domain 9.  69. Create or change credentialing and/or licensure standards | Repeated cross-sectional study.  Interrupted time series analysis (no control) (of three nation-wide representative databases). | Interventions: (i): introduction of GP guideline (ii): introduction of health insurance coverage for smoking cessation treatment (iii): abolition of health insurance coverage for smoking cessation treatment  **Prescription for all cessation medications (NRT/varenicline/bupropion):** Pre- (i) and (ii) and (iii): 0.02 quarterly change in the number of prescriptions per 1,000 smokers, 95% CI: -0.09 to -1.14, p=0.676. Post- (i): 0.84 change in the quarterly level of prescriptions per 1,000 smokers, 95% CI: -2.04 to -3.71, p=0.560. (Immediate, level change) Post- (i): -0.10 change in the trend in quarterly number of prescriptions per 1,000 smokers, 95% CI: -0.40 to -0.20, p=0.499. (Trend change) Post- (ii): 6.31 change in the quarterly level of prescriptions per 1,000 smokers, 95% CI: 2.86 to 9.76, p=0.001. (Immediate, level change)  **Prescription for bupropion:** Pre- (i) and (ii) and (iii): 0.02 quarterly change in the number of prescriptions per 1,000 smokers, 95% CI: -0.03 to -0.07, p=0.374. Post- (i): 0.12 change in the quarterly level of prescriptions per 1,000 smokers, 95% CI: -1.13 to -1.37, p=0.845. (Immediate, level change) Post- (i): -0.05 change in the trend in quarterly number of prescriptions per 1,000 smokers, 95% CI: -0.18 to -0.08, p=0.475. (Trend change) Post- (ii): 0.91 change in the quarterly level of prescriptions per 1,000 smokers, 95% CI:-0.59 to 2.41, p=0.227. (Immediate, level change)  **Prescription for NRT:** Pre- (i) and (ii) and (iii): -0.00 quarterly change in the number of prescriptions per 1,000 smokers, 95% CI: -0.03 to -0.02, p=0.832. Post- (i): 0.11 change in the quarterly level of prescriptions per 1,000 smokers, 95% CI: -0.52 to -0.73, p=0.735. (Immediate, level change) Post- (i): -0.00 change in the trend in quarterly number of prescriptions per 1,000 smokers, 95% CI: -0.07 to -0.07, p=0.986. (Trend change) Post- (ii): 1.97 change in the quarterly level of prescriptions per 1,000 smokers, 95% CI: 1.23 to 2.72, p<0.000. (Immediate, level change)  **Prescription for varenicline:** Pre- (i) and (ii) and (iii): 0.01 quarterly change in the number of prescriptions per 1,000 smokers, 95% CI: -0.03 to -0.05, p=0.644. Post- (i): did not assess. Post- (i): did not assess. Post- (ii): 2.97 change in the quarterly level of prescriptions per 1,000 smokers, 95% CI: 1.30 to 4.64, p=0.0001. (Immediate, level change)  **Dispensed prescription for all cessation medications (NRT/varenicline/bupropion):** Pre- (i) and (ii) and (iii): -0.01 quarterly change in the number of (dispensed) prescriptions per 1,000 smokers, 95% CI: -0.16 to -0.15, p=0.924. Post- (i): 2.68 change in the quarterly level of (dispensed) prescriptions per 1,000 smokers, 95% CI: -1.23 to -6.59, p=0.173. (Immediate, level change) Post- (i): 0.39 change in the trend in quarterly number of (dispensed) prescriptions per 1,000 smokers, 95% CI: -0.02 to -0.79, p=0.060. (Trend change) Post- (ii): 17.26 change in the quarterly level of (dispensed) prescriptions per 1,000 smokers, 95% CI: 12.53 to 21.98, p<0.000. (Immediate, level change) Post- (iii): -21.56 change in quarterly level of (dispensed) prescriptions per 1,000 smokers, 95% CI: -25.93 to -17.19, p<0.000. (Immediate, level change)  **Dispensed prescription for bupropion:** Pre- (i) and (ii) and (iii): 0.03 quarterly change in the number of (dispensed) prescriptions per 1,000 smokers, 95% CI: -0.03 to -0.08, p=0.292. Post- (i): -0.31 change in the quarterly level of (dispensed) prescriptions per 1,000 smokers, 95% CI: -1.64 to -1.03, p=0.645. (Immediate, level change) Post- (i): -0.02 change in the trend in quarterly number of (dispensed) prescriptions per 1,000 smokers, 95% CI: -1.28 to 1.92, p=0.688. (Trend change) Post- (ii): 0.32 change in the quarterly level of (dispensed) prescriptions per 1,000 smokers, 95% CI: -1.28 to 1.92, p=0.688. (Immediate, level change) Post- (iii): -0.79 change in quarterly level of (dispensed) prescriptions per 1,000 smokers, 95% CI: -2.27 to 0.69, p=0.288. (Immediate, level change)  **Dispensed prescription for NRT:** Pre- (i) and (ii) and (iii): 0.06 quarterly change in the number of (dispensed) prescriptions per 1,000 smokers, 95% CI: 0.01 to 0.11, p=0.026. Post- (i): 0.20 change in the quarterly level of (dispensed) prescriptions per 1,000 smokers, 95% CI: -1.15 to -1.55, p=0.768. (Immediate, level change) Post- (i): 0.01 change in the trend in quarterly number of (dispensed) prescriptions per 1,000 smokers, 95% CI: -0.13 to 0.15, p=0.929. (Trend change) Post- (ii): 5.45 change in the quarterly level of (dispensed) prescriptions per 1,000 smokers, 95% CI: 3.82 to 7.08, p<0.000. (Immediate, level change) Post- (iii): -5.86 change in quarterly level of (dispensed) prescriptions per 1,000 smokers, 95% CI: -7.37 to -4.35, p<0.000. (Immediate, level change)  **Dispensed prescription for varenicline**: Pre- (i) and (ii) and (iii): 0.02 quarterly change in the number of (dispensed) prescriptions per 1,000 smokers, 95% CI: -0.07 to -0.12, p=0.618. Post- (i): did not assess. Post- (i): did not assess. Post- (ii): 4.72 change in the quarterly level of (dispensed) prescriptions per 1,000 smokers, 95% CI: 0.65 to 8.79, p=0.024. (Immediate, level change) Post- (iii): -11.30 change in quarterly level of (dispensed) prescriptions per 1,000 smokers, 95% CI: -16.05 to -6.55, p<0.000. (Immediate, level change)  **Smoking prevalence:** Pre- (i) and (ii) and (iii): -0.14 quarterly change in smoking prevalence (%), 95% CI: -0.20 to -0.09, p<0.000. Post- (i): -0.15 change in the quarterly level of smoking prevalence (%), 95% CI: -1.60 to -1.30, p=0.835. (Immediate, level change) Post- (i): 0.17 change in the trend in quarterly smoking prevalence (%), 95% CI: 0.02 to 0.32, p=0.028. (Trend change) Post- (ii): -2.90 change in the quarterly level of smoking prevalence (%), 95% CI: 4.61 to -1.11, p=0.002. (Immediate, level change) Post- (iii): 1.16 change in quarterly level of smoking prevalence (%), 95% CI: 0.50 to 2.8, p=0.156. (Immediate, level change) |

The included studies are ordered by implementation strategy domain (5, 7, 8 and 9 and ‘Multiple domains’). Within the domains, the studies are ordered by implementation strategy category then alphabetically by first author surname.

(Wright, 2018) was excluded from narrative synthesis as it was at critical risk, but it is included in this table.
